# Alkyl *N*-Benzylthiocarbamates, the First Copper(II) Ion-Chelating Tyrosinase Inhibitors with a Thiocarbamate Group and ROS-Scavenging Activity, Exhibit Different Inhibitory Activities Depending on the Origin of Tyrosinase

**DOI:** 10.3390/antiox15010039

**Published:** 2025-12-26

**Authors:** Hee Jin Jung, Hyeon Seo Park, Yeonsoo Jeong, Ga Young Kim, Hyunju Lee, Hye Soo Park, Hye Jin Kim, Hyunhee Ju, Hyejin Kang, Yujin Park, Hae Young Chung, Hyung Ryong Moon

**Affiliations:** 1 College of Pharmacy and Research Institute for Drug Development, Pusan National University, Busan 46241, Republic of Korea; hjjung2046@pusan.ac.kr (H.J.J.); gustj6956@pusan.ac.kr (H.S.P.); jysoo627@pusan.ac.kr (Y.J.); kgy9905@pusan.ac.kr (G.Y.K.); lhj6384770@pusan.ac.kr (H.L.); hyesoo0713@pusan.ac.kr (H.S.P.); khj3358@pusan.ac.kr (H.J.K.); hyunh@pusan.ac.kr (H.J.); dirgowls22@pusan.ac.kr (H.K.); hyjung@pusan.ac.kr (H.Y.C.); 2 Department of Medicinal Chemistry, New Drug Development Center, Daegu-Gyeongbuk Medical Innovation Foundation, Daegu 41061, Republic of Korea; pyj1016@kmedihub.re.kr

**Keywords:** tyrosinase, thiocarbamate, melanin, Cu^2+^ ion, ROS, PTU, zebrafish, browning

## Abstract

*N*-Benzylthiocarbamate (NBTC) analogs **1**–**10** were synthesized as potential Cu^2+^-chelating tyrosinase inhibitors. Most analogs exhibited strong Cu^2+^-chelating activity, but none inhibited mushroom tyrosinase (mTYR) activity better than kojic acid (KA). However, owing to their potent cellular TYR inhibitory activity, all analogs, except **8**, inhibited melanin formation in B16F10 cells more than KA. Analogs **3**, **4**, and **9** exhibited stronger antimelanogenic properties than *N*-phenylthiourea. The TYR inhibitory activity of the analogs of mTYR and B16F10 TYR was probably different because mTYR and mammalian TYR have different structural characteristics. All analogs had a potency to significantly scavenge reactive oxygen species (ROS). Analog **1** was very effective in reducing browning in potato juice. Furthermore, analog **3** inhibited zebrafish larval pigmentation 2000 times more potently than KA and was more effective than *N*-phenylthiourea. It is believed that their capacity to scavenge ROS amplifies their antimelanogenic effects. Exogenously added CuSO_4_ attenuated the inhibitions of cellular TYR activity and melanin formation in B16F10 cells caused by analog **9**. This result might have been due to the externally added Cu^2+^ ions forming chelates with **9**. The differential TYR inhibitory activity of NBTC analogs appeared to be due to their high sensitivity to interactions with TYRs of different origins.

## 1. Introduction

Tyrosinase (TYR), which is involved in melanin synthesis, is found in several organisms, including fish, bacteria, plants, fungi, animals, and humans. It is a type-3 Cu^2+^-containing metalloenzyme with two Cu^2+^ ions at its active site [1]. Monophenol and diphenol compounds are oxidized to diphenol and quinone compounds, respectively, by the oxidase TYR. TYR utilizes l-tyrosine as a substrate during melanogenesis, hydroxylates it to l-dopa via monophenolase activity, and then oxidizes l-dopa to dopaquinone via diphenolase activity. Dopaquinone activates the eumelanin and pheomelanin pathways for melanin biosynthesis [2]. In the absence of thiols such as cysteine and glutathione, dopaquinone participates in the eumelanin synthesis pathway, which involves TYR-related protein (TRP)-1, TRP-2, and TYR. In contrast, dopaquinone enters the pheomelanin synthesis pathway, which does not require enzymes such as TRP-1, TRP-2, and TYR, when thiols are present. The color of pheomelanin is red to yellow, while that of eumelanin is black to brown [3,4]. Melanin is the main pigment responsible for the color of hair, feathers, and pupils. Additionally, the ratio of eumelanin to pheomelanin is the main determinant of human skin color. Moreover, melanin causes fruits and vegetables to brown, which lowers their marketability [5]. Melanin production is primarily induced by reactive oxygen species (ROS) and ultraviolet radiation [6]. Overproduction of melanin in the body or a particular skin area can lead to disorders related to hyperpigmentation or aesthetic issues [7,8]. Arbutin, kojic acid (KA), and hydroquinone are antimelanogenic drugs currently used in clinical settings; however, their clinical efficacy is insufficient, and they have several adverse effects, including vitiligo, skin irritation, contact dermatitis, and carcinogenesis [9,10,11]. Therefore, antimelanogenic drugs with stronger therapeutic effects and fewer adverse effects should be developed.

The inhibition of TYR glycosylation and maturation, inhibition of melanosome transfer to keratinocytes, promotion of TYR degradation, inhibition of TYR mRNA transcription, inhibition of TYR activity, and promotion of skin turnover are some of the methods used to treat hyperpigmentation. Of these strategies, inhibition of TYR activity is considered the most effective way to treat hyperpigmentation-related disorders. The central Cu^2+^-binding domain of TYR, an enzyme that limits melanogenesis, is highly conserved [12]. Each of the two Cu^2+^ ions that constitute the active site of TYR is ligated to three histidine amino acids [13]. The tetramers that make up mushroom TYR (mTYR) differ from the monomers that make up mammalian TYR. Additionally, mTYR is found in the cytosol, whereas mammalian TYR is attached to the melanosome membrane in melanocytes [14]. Furthermore, mammalian TYR differs from mTYR in that it is a glycosylated protein [15]. Some TYR inhibitors seem to have varying TYR inhibitory activities because of the distinctions between mTYR and mammalian TYR [16,17].

Excessive ROS generation significantly contributes to skin issues, including excessive melanin production, aging, and wrinkles [18,19]. Since melanin plays a role in protecting genetic material, excessive melanin biosynthesis in response to excessive ROS production is a type of cell protection mechanism [20]. Given that ROS stimulate the production of melanin, antioxidants that scavenge ROS can inhibit melanin production [21,22,23].

**Figure 1:**
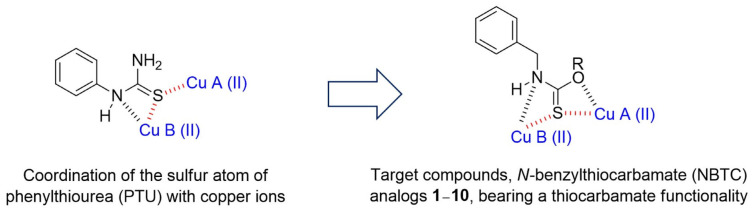
Coordination picture between the sulfur atom of phenylthiourea and the Cu^2+^ ions in the catechol oxidase active site and a plausible illustration of *N*-benzylthiocarbamate analogs **1**–**10** chelating two Cu^2+^ ions within the TYR active site.

**Figure 2:**
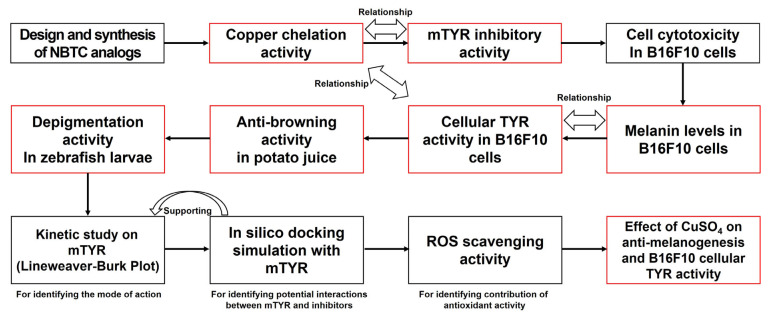
Diagram illustrating the experimental framework and rationale. Experiments involving Cu^2+^ ion chelation, TYR activity, and melanin production are shown in red boxes.

Several biological effects, including acetylcholinesterase inhibition [24], histone deacetylase inhibition [25], anticancer [26], antifungal [27,28], and antimicrobial [29] activities, have been demonstrated for compounds with thiocarbamate (-NC(=S)O-; carbamothioate) functionalities. *N*-Phenylthiourea (PTU), one of the most powerful TYR inhibitors, chelates Cu^2+^ ions using its sulfur and nitrogen atoms at the active site of catechol oxidase, an enzyme structurally and functionally related to TYR (Figure 1) [30,31]. Sulfur atoms can efficiently coordinate with Cu^2+^ ions [32]. Considering these discoveries, we designed and synthesized several *N*-benzylthiocarbamate (NBTC) analogs that were anticipated to function as TYR inhibitors by chelating Cu^2+^ ions. Similar to PTU, it was believed that one of the two Cu^2+^ ions in TYR was chelated by the nitrogen and sulfur atoms of the NBTC analogs, as shown in Figure 1. Additionally, the oxygen and sulfur atoms of the thiocarbamate functionality are believed to chelate the residual Cu^2+^ ion in TYR. Although TYR inhibitors with thiocarbamate moieties embedded in oxazole-type heterocycles have been reported [33,34], to the best of our knowledge, no substance with an alkyl thiocarbamate functionality has been identified as a TYR inhibitor. The objective of the following experiments was to confirm whether NBTC analogs could inhibit TYR activity by chelating with Cu^2+^ ions within TYR, thereby halting melanin production. Therefore, all aspects of NBTC analogs were investigated, including Cu^2+^ ion-chelating capability, inhibition of mTYR activity, inhibition of cellular TYR activity and melanin production in B16F10 cells, anti-browning effect in potato juice, in vivo depigmentation effect in zebrafish larvae, and the effect of exogenously added Cu^2+^ ions on cellular TYR activity and melanin production of NBTC analogs in B16F10 cells (Figure 2).

## 2. Materials and Methods

### 2.1. General Chemical Methods

The reaction progress was tracked using Silica Gel 60F_254_ thin-layer chromatography (Merck, Darmstadt, Germany). For compound separation, silica gel (60 Å; MP Silica 40–63) was utilized as a packing material in flash column chromatography. Anhydrous toluene was prepared by distillation over CaH_2_, and the chemicals were supplied by Thermo Fisher Scientific (Waltham, MA, USA) and SEJIN CI Co. (Seoul, Republic of Korea). The NBTC analogs were structurally characterized by ^13^C and ^1^H nuclear magnetic resonance (NMR) spectroscopy. Using a JEOL ECZ400S instrument (JEOL Ltd., Tokyo, Japan), ^13^C and ^1^H NMR data were obtained. Peaks corresponding to the compound structure are expressed in coupling constant (Hz; *J*) and chemical shift (ppm; *δ*). The multiplicity of ^1^H NMR data is expressed as follows: t = triplet, q = quartet, s = singlet, d = doublet, m = multiplet, and brs = broad singlet. Using the residual solvent peak as an internal standard (^1^H and ^13^C NMR: 7.26 and 77.0 ppm for CDCl_3_ [deuterated chloroform], respectively), the NMR spectra were obtained. The NMR spectra of **1**–**10** are also included in the Supplementary Materials. ^13^C and ^1^H NMR spectra of all compounds were measured in CDCl_3_ at 100 and 400 MHz, respectively. The high resolution (HR) liquid chromatography tandem mass spectrometer (Sciex’s ZenoTOF 7600 model; Toronto, ON, Canada) using the ESI(+)-TOF method was used to collect HR mass spectroscopy (MS) data.

### 2.2. Preparation of NBTC Analogs ***1***–***10***

#### 2.2.1. General Protocol for the Preparation of Alkyl Benzylcarbamothioates **1**–**4** and **10** [35,36]

Benzyl isothiocyanate (100 mg, 0.67 mmol) was refluxed for 2–3 day in alcohol (2 mL; methanol for **1**, ethanol for **2**, 1-propanol for **3**, 1-butanol for **4**, and 2-propanol for **10**). Hexane:ethyl acetate (8:1–15:1) was used as the eluent in silica gel chromatography to purify the residue resulting from evaporation under reduced pressure.

##### O-Methyl Benzylcarbamothioate (**1**) [35]

^1^H NMR (chloroform-*d*) *δ* 7.37–7.23 (m, 5H), 6.92 (brs, 0.35H), 6.49 (brs, 0.65H), 4.75 (d, 1.3H, *J* = 5.6 Hz, NCH_2_), 4.45 (d, 0.7H, *J* = 5.6 Hz, NCH_2_), 4.08 (s, 1.05H, OCH_3_), 4.01 (s, 1.95H, OCH_3_); ^13^C NMR *δ* 191.6, 190.8, 136.9, 136.5, 129.0, 128.9, 128.0, 128.0, 128.0, 127.7, 58.6, 57.4, 49.5, 47.4; yield, 99%.

##### O-Ethyl Benzylcarbamothioate (**2**) [35]

^1^H NMR (chloroform-*d*) *δ* 7.37–7.24 (m, 5H), 6.88 (brs, 0.36H), 6.44 (brs, 0.64H), 4.75 (d, 1.28H, *J* = 5.6 Hz, NCH_2_), 4.57 (q, 0.72H, *J* = 7.2 Hz, CH_2_CH_3_), 4.51 (q, 1.28H, *J* = 7.2 Hz, CH_2_CH_3_), 4.44 (d, 0.72H, *J* = 6.0 Hz, NCH_2_), 1.35 (t, 1.08H, *J* = 7.2 Hz, Me), 1.32 (t, 1.92H, *J* = 7.2 Hz, Me); ^13^C NMR *δ* 190.8, 190.0, 137.0, 136.6, 128.9, 128.9, 128.0, 128.0, 128.0, 127.8, 68.2, 66.7, 49.3, 47.3, 14.3, 14.3; yield, 99%.

##### O-Propyl Benzylcarbamothioate (**3**) [35]

^1^H NMR (chloroform-*d*) *δ* 7.41–7.24 (m, 5H), 6.90 (brs, 0.35H), 6.45 (brs, 0.65H), 4.75 (d, 1.3H, *J* = 5.6Hz, NCH_2_), 4.46 (t, 0.7H, *J* = 6.4 Hz, OCH_2_), 4.45 (d, 0.7H, *J* = 5.6 Hz, NCH_2_), 4.41 (t, 1.3H, *J* = 6.8Hz, OCH_2_), 1.79–1.68 (m, 2H, CH_2_CH_2_CH_3_), 1.00–0.92 (m, 3H, Me); ^13^C NMR *δ* 190.9, 190.1, 137.0, 136.6, 128.9, 128.9, 128.0, 128.0, 128.0, 127.7, 73.8, 72.4, 49.3, 47.3, 22.1, 22.1, 10.4, 10.4; yield, 80%.

##### O-Butyl Benzylcarbamothioate (**4**)

^1^H NMR (chloroform-*d*) *δ* 7.37–7.23 (m, 5H), 6.93 (brs, 0.4H), 6.44 (brs, 0.6H), 4.75 (d, 1.2H, *J* = 5.6 Hz, NCH_2_), 4.50 (t, 0.8H, *J* = 6.8 Hz, OCH_2_), 4.46 (t, 1.2H, *J* = 6.8 Hz, OCH_2_), 4.44 (d, 0.8H, *J* = 5.6 Hz, NCH_2_), 1.74–1.65 (m, 2H, OCH_2_CH_2_), 1.45–1.32 (m, 2H, OCH_2_CH_2_CH_2_), 0.94 (t, 1.8H, *J* = 7.2 Hz, Me), 0.92 (t, 1.2H, *J* = 7.2 Hz, Me); ^13^C NMR *δ* 190.9, 190.1, 137.0, 136.7, 128.9, 128.9, 128.0, 128.0, 128.0, 127.7, 72.1, 70.7, 49.3, 47.3, 30.8, 30.7, 19.1, 19.1, 13.8, 13.7; yield, 83%; HRMS (ESI+) *m*/*z* C_12_H_18_NOS (M+H)^+^ calcd 224.1104, obsd 224.1108.

##### O-Isopropyl Benzylcarbamothioate (**10**) [37]

^1^H NMR (chloroform-*d*) *δ* 7.37–7.23 (m, 5H), 6.83 (brs, 0.4H), 6.37 (brs, 0.6H), 5.63–5.53 (m, 1H, OCH), 4.75 (d, 1.2H, *J* = 5.6 Hz, NCH_2_), 4.42 (d, 0.8H, *J* = 6.0 Hz, NCH_2_), 1.32 (d, 2.4H, *J* = 5.6 Hz, 2×CH_3_), 1.30 (d, 3.6H, *J* = 5.6 Hz, Me×2); ^13^C NMR *δ* 190.0, 189.3, 137.0, 136.8, 128.9, 128.9, 128.1, 127.9, 127.9, 127.7, 76.2, 74.1, 49.2, 47.2, 21.9, 21.8; yield, 39%.

#### 2.2.2. General Protocol for the Preparation of Alkyl Benzylcarbamothioates **5**–**9** [36]

Benzyl isothiocyanate (100 mg, 0.67 mmol) was reacted with the appropriate alcohol (1.0 equiv.; 1-pentanol for **5**, isoamyl alcohol for **6**, 4-phenyl-1-butanol for **7**, cyclopentanol for **8**, and cyclohexanol for **9**) by heating toluene (1 mL) at 100 °C for 2–3 days. Hexane:ethyl acetate (14:1–17:1) was used as the eluent in silica gel chromatography to purify the residue resulting from evaporation under reduced pressure.

##### O-Pentyl Benzylcarbamothioate (**5**)

^1^H NMR (chloroform-*d*) *δ* 7.37–7.24 (m, 5H), 6.91 (brs, 0.4H), 6.44 (brs, 0.6H), 4.75 (d, 1.2H, *J* = 5.6 Hz, NCH_2_), 4.49 (t, 0.8H, *J* = 6.8 Hz, OCH_2_), 4.46–4.43 (m, 2H, NCH_2_, OCH_2_), 1.75–1.67 (m, 2H, OCH_2_CH_2_), 1.37–1.31 (m, 4H, OCH_2_CH_2_CH_2_CH_2_), 0.92–0.87 (m, 3H, Me); ^13^C NMR *δ* 190.9, 190.2, 137.0, 136.7, 128.9, 128.9, 128.0, 128.0, 127.9, 127.6, 72.4, 71.0, 49.3, 47.3, 28.5, 28.4, 28.1, 28.1, 22.4, 22.4, 14.0, 14.0; yield, 92%; HRMS (ESI+) *m*/*z* C_13_H_20_NOS (M+H)^+^ calcd 238.1260, obsd 238.1262.

##### O-Isopentyl Benzylcarbamothioate (**6**)

^1^H NMR (chloroform-*d*) *δ* 7.37–7.23 (m, 5H), 6.89 (brs, 0.4H), 6.43 (brs, 0.6H), 4.75 (d, 1.2H, *J* = 5.6 Hz, NCH_2_), 4.53 (t, 0.8H, *J* = 6.8 Hz, OCH_2_), 4.49 (t, 1.2H, *J* = 6.8Hz, OCH_2_), 1.74–1.56 (m, 3H, CH_2_CH), 0.93 (d, 3.6H, *J* = 6.4 Hz, Me×2), 0.92 (d, 2.4H, *J* = 6.0 Hz, Me×2); ^13^C NMR *δ* 190.9, 190.2, 137.0, 136.6, 128.9, 128.9, 128.0, 128.0, 128.0, 127.6, 71.0, 69.5, 49.4, 47.3, 37.5, 37.4, 25.1, 25.1, 22.6, 22.6; yield, 45%; HRMS (ESI+) *m*/*z* C_13_H_20_NOS (M+H)^+^ calcd 238.1260, obsd 238.1263.

##### O-(4-Phenylbutyl) Benzylcarbamothioate (**7**)

^1^H NMR (chloroform-*d*) *δ* 7.36–7.14 (m, 10H), 6.91 (brs, 0.4H), 6.43 (brs, 0.6H), 4.75 (d, 1.2H, *J* = 5.6 Hz, NCH_2_), 4.53 (t, 0.8H, *J* = 6.4 Hz, OCH_2_), 4.49 (t, 1.2H, *J* = 6.4 Hz, OCH_2_), 4.43 (d, 0.8H, *J* = 6.0 Hz, NCH_2_), 2.68–2.62 (m, 2H, CH_2_Ph), 1.80–1.63 (m, 4H, OCH_2_CH_2_CH_2_); ^13^C NMR *δ* 190.8, 190.1, 142.1, 142.0, 136.9, 136.6, 128.9, 128.9, 128.5, 128.5, 128.4, 128.4, 128.1, 128.0, 128.0, 127.6, 126.0, 125.9, 72.1, 70.6, 49.4, 42.3, 35.6, 35.5, 28.4, 28.3, 27.8, 27.6; yield, 75%; HRMS (ESI+) *m*/*z* C_18_H_22_NOS (M+H)^+^ calcd 300.1417, obsd 300.1421.

##### O-Cyclopentyl Benzylcarbamothioate (**8**)

^1^H NMR (chloroform-*d*) *δ* 7.37–7.22 (m, 5.42H, Ph, NH), 6.39 (brs, 0.58H, NH), 5.74–5.70 (m, 1H, OCH), 4.74 (d, 1.16H, *J* = 5.6 Hz, NCH_2_), 4.39 (d, 0.84H, *J* = 6.0 Hz, NCH_2_), 1.94–1.55 (m, 8H, CH_2_CH_2_CH_2_CH_2_); ^13^C NMR *δ* 190.3, 189.5, 137.0, 136.9, 128.9, 128.9, 128.1, 127.9, 127.9, 127.5, 85.4, 83.5, 49.3, 47.2, 32.9, 32.9, 24.0, 23.8; yield, 77%; HRMS (ESI+) *m*/*z* C_13_H_18_NOS (M+H)^+^ calcd 236.1104, obsd 236.1108.

##### O-Cyclohexyl Benzylcarbamothioate (**9**)

^1^H NMR (chloroform-*d*) *δ* 7.37–7.24 (m, 5H), 6.88 (brs, 0.4H), 6.39 (brs, 0.6H), 5.39–5.31 (m, 1H, OCH), 4.75 (d, 1.2H, *J* = 5.6 Hz, NCH_2_), 4.44 (d, 0.8H, *J* = 6.0 Hz, NCH_2_), 2.02–1.89 (m, 2H, cyclohexyl), 1.76–1.21 (m, 8H, cyclohexyl); ^13^C NMR *δ* 190.0, 189.2, 137.0, 136.8, 128.9, 128.9, 128.1, 127.9, 127.9, 127.6, 80.5, 78.9, 49.2, 47.2, 31.7, 31.4, 25.4, 25.4, 23.8, 23.5; yield, 45%; HRMS (ESI+) *m*/*z* C_14_H_20_NOS (M+H)^+^ calcd 250.1260, obsd 250.1260.

### 2.3. Assay for Cu^2+^ Ion Chelation [38]

CuSO_4_ aqueous solution (6.27 mM, 10 μL) was added to each well of a 96-well plate that contained pyrocatechol violet (PCV) (4 mM, 6 μL; Tokyo Chemical Industry, Tokyo, Japan), NBTC analogs (10 μL, 3.06 mM; final concentration: 100 μM), and AcOH-AcONa buffer (pH 6.0, 50 mM; 280 μL). The plate was cultivated for 20 min at 18 °C. To ascertain whether NBTC analogs could chelate Cu^2+^ ions, OD at 632 nm was measured using a VersaMax^®^ microplate (VM) reader (Molecular Devices, Sunnyvale, CA, USA). The Cu^2+^ chelation activity (%) is equal to 100 × [(OD_CNTL_ − OD_ANLG_)/OD_CNTL_], where OD_CNTL_ and OD_ANLG_ represent the ODs of the control and analogs.

### 2.4. Assay for mTYR Activity [39]

A solution of mTYR (10 μL; 750 units/mL; Sigma-Aldrich, St. Louis, MO, USA) dissolved in phosphate buffer was added to each well of a 96-well plate containing test samples (20 μL in dimethyl sulfoxide [DMSO]; analogs **1**–**10** and KA [reference material]) and substrate solution (170 μL) comprising phosphate buffer (17.2 mM; pH 6.5) and l-DOPA or l-tyrosine (345 μM). Analogs **1**–**10** were tested at 4, 20, and 100 μM for l-DOPA and 2, 10, and 50 μM for l-tyrosine, and KA was tested at 2, 10, and 50 μM for l-tyrosine and l-DOPA. A VM reader was used to measure the absorbance of each well at 475 nm after cultivating the mixture for 30 min for l-tyrosine and for 20 min for l-DOPA at 37 °C to ascertain the TYR activity.

### 2.5. Cell Culture

Human foreskin fibroblasts (Hs27), human keratinocytes (HaCaT), and mouse melanoma cells (B16F10) were procured from the American Type Culture Collection (Manassas, VA, USA). These cells were raised at 37 °C using a mixture of 10% fetal bovine serum and Dulbecco’s modified Eagle’s medium, which included 100 units/mL of streptomycin–penicillin solution, in humidified air with 5% CO_2_. All cell culture reagents, including Dulbecco’s phosphate-buffered saline (DPBS), were acquired from Gibco Invitrogen Corporation (Waltham, MA, USA).

### 2.6. Assay for Cell Viability [40]

NBTC analogs **1**–**10** were tested for viability in HaCaT, B16F10, and Hs27 cells using an EZ-Cytox colorimetric kit (EZ-500, DoGenBio, Seoul, Republic of Korea). Every cell in every well of a 96-well plate (1 × 10^3^ HaCaT and Hs27 cells/well or 5 × 10^2^ B16F10 cells/well) was cultivated for 24 h at 37 °C with 5% CO_2_ to stabilize the cells. Analogs (0, 5, 10, and 20 μM) were administered to the cultivated cells, which were then incubated for 24 h for HaCaT and Hs27 cells and 72 h for B16F10 cells. After adding a 10 μL aliquot of EZ-Cytox solution to each well, the plate was incubated for an additional 2 h. A VM reader was used to measure the well absorbance at 450 nm.

### 2.7. Assay for Melanin Contents in B16F10 Cells [40]

A 6-well plate was used for the experiment. After inoculation at a density of 5 × 10^3^ cells/well, B16F10 cells were cultivated for 24 h in an environment that was the same as the conditions used for cell culture. The cultivated cells were treated with samples (**1**–**10** [20 μM] and KA [reference material; 20 μM]) for 1 h in a preliminary experiment. In a main experiment, analogs **3**, **4**, and **9** [20, 10, and 5 μM], selected from the preliminary experimental results, and positive control substances (KA [20 μM] and PTU [20, 10, and 5 μM]) were exposed to cultivated cells for 1 h. Subsequently, they were exposed to 3-isobutyl-1-methylxanthine (IBMX) and alpha-melanocyte-stimulating hormone (α-MSH) (200 and 1 μM, respectively). Following a 72 h incubation, the incubated cells were rinsed with DPBS and then detached from the plate well using trypsin-EDTA. After the separated cells were lysed with 1N NaOH solution (0.1 mL), they were centrifuged at 4 °C and 10,000× *g* for 10 min. The OD of each well at 405 nm was obtained using a VM reader to ascertain the intracellular melanin contents after a 1 h incubation at 60 °C. The total protein concentration was determined using a Pierce BCA Protein Assay Kit from Thermo Scientific (Waltham, MA, USA), which was used to normalize the results. The following formula was used to determine melanin levels: melanin (%) = (OD_sample_/OD_control_) × 100.

### 2.8. Assay for B16F10 Cellular TYR Activity [41]

Within a 6-well plate, B16F10 cells (5 × 10^3^ cells/well) were raised for 24 h before being exposed to the test sample (analogs **3**, **4**, and **9** [5, 10, and 20 μM]) and KA [reference material; 20 μM] for 1 h. After 72 h of treatment with IBMX and α-MSH (200 and 1 μM, respectively), the cells were rinsed with DPBS. A lysis buffer solution (0.1 mL) composed of Triton X-100 (20%), phenylmethylsulfonyl fluoride (2 mM), and phosphate buffer (pH 6.5; 50 mM) in a 5:5:90 ratio was added, kept for 30 min at −80 °C, and then defrosted. The cell lysates were centrifuged for 30 min at 4 °C and 10,000× *g*. l-DOPA (10 mM; 20 µL) was combined with the lysate supernatant (80 µL) in each well of a 96-well plate. To calculate the B16F10 cellular TYR activity, the OD of each well at 475 nm was measured at 10 min intervals for 1 h using a VM reader at 37 °C.

### 2.9. Assay for In Situ Cellular TYR Activity in B16F10 Cells [42]

Each well of a 24-well plate holding 1 × 10^3^ B16F10 cells was cultivated with 5% CO_2_ at 37 °C for 23 h. After 1 h of exposure to KA (20 μM; positive material) or NBTC analogs (**3**, **4**, and **9**; 20, 10, and 5 μM), the cultivated cells were treated with IBMX (200 μM) and α-MSH (1 μM) for 72 h. Following paraformaldehyde solution (4%) fixation and DPBS (pH 7.4) washing, the cells were permeabilized for 3 min using 0.1% Triton X-100. l-DOPA (500 μL; 2 mM) was added after the DPBS rinse, and the mixture was cultivated at 37 °C for 2 h. Melanin-stained cells were imaged using a Moticam 5MP camera (Hong Kong) mounted on a Nikon Eclipse Ts2 inverted microscope (Tokyo, Japan).

### 2.10. Assay for Browning of Potato Juice [43,44]

In April 2025, the potatoes (*Solanum tuberosum*) used in this study were obtained from a local market in Busan, Republic of Korea. After the potatoes (145 g) were peeled and cut, they were ground using a blender (Tefal, Blend Force glass BL317166, Rumilly, France). To remove the precipitates, the whole potato ground extract was placed into 15 mL conical tubes and centrifuged at 18 °C at 4000× *g* (Labogene, Seoul, Republic of Korea, 416) for 5 min. Fresh potato juice was obtained by collecting the supernatants in 2 mL centrifuge tubes and centrifuging them at 10,000× *g* (Labogene, Seoul, Republic of Korea, 1730R) for 10 min at 18 °C to eliminate any remaining precipitates. In each well of a 96-well plate, an aliquot (100 µL) of potato extract juice was combined with 100 µL of a test sample (0.2 mM of analogs **1**–**10** and KA [positive control] for a preliminary experiment and 0.2, 0.6, and 2 mM of analog **1** and three positive controls [KA, ascorbic acid, and Trolox] for a main experiment). A VM reader set to 405 nm was used to measure the browning intensity of the potato juice while it was kept for 24 h at 37 °C. The rate of increase in browning intensity was calculated as follows: browning intensity (%) = [(Abs_SPL′_/Abs_SPL_)] × 100, where Abs_SPL′_ and Abs_SPL_ are the ODs of the tested samples at each observed hour and 0 h, respectively.

### 2.11. Assay for Depigmentation of Zebrafish Larvae [45,46]

Using an E3-MB solution that contained methylene blue (0.001%) and an E3 solution made by combining NaCl (5 mM), MgSO_4_ (0.33 mM), KCl (0.17 mM), and CaCl_2_ (0.33 mM), zebrafish embryos were raised prior to use. Pronase^®^ (Sigma-Aldrich, St. Louis, MO, USA) was utilized to dechorionate zebrafish embryos at 24 h post-fertilization (hpf). Following a 4 h incubation period, for a preliminary experiment, NBTC analogs **1**–**10** (0.1 mM) were exposed to six zebrafish embryos (*n* = 1) in each well of a 48-well plate. KA (20 mM) was utilized as a positive material. The SMZ745T stereomicroscope (Nikon, Tokyo, Japan) was used to capture images of the zebrafish larvae after they had been incubated for 44 h at 28 °C to assess their pigmentation level. For a main experiment (*n* = 3), NBTC analogs **1**–**3** and **10** were selected from the above-mentioned preliminary experiment and used at three concentrations (0.01, 0.03, and 0.1 mM). KA (20 mM) and PTU (0.01, 0.03, and 0.1 mM) were utilized as positive materials in this experiment. The main experiment was conducted in the same manner as the preliminary experiment. In the main experiment, photographs of the pigmented regions of the zebrafish taken from the dorsal and lateral views were transformed into values using an ATTO CS analyzer (ATTO, Tokyo, Japan).

### 2.12. Kinetic Studies [47,48]

mTYR (150 units/mL; 20 μL) was combined with analog (**1**, **5**, **7**, and **9**; final concentrations: 80, 40, 20, and 0 μM; 10 μL) in DMSO and an aqueous substrate solution (170 μL) consisting of sodium phosphate buffer (final concentration: 14.7 mM; pH 6.5) and l-DOPA (final concentrations: 16, 8, 4, 2, 1, and 0.5 mM or 16, 8, 4, 2, and 1 mM) in each well of a 96-well plate. The increase in the OD at 475 nm was recorded using a VM reader. L-B plots and Dixon plots for each analog were obtained using the initial rate of dopachrome formation obtained from various combinations of l-DOPA concentration and the analog concentration.

### 2.13. In Silico Docking Simulation with mTYR [49,50]

NBTC analogs **1**, **5**, **7**, and **9** were subjected to docking simulations using AutoDock Vina 1.2.0 (The Scripps Research Institute, La Jolla, San Diego, CA, USA). PDB (ID: 2Y9X) provided the 3D X-ray co-crystal structure of mTYR (*Agaricus bisporus*) [14]. Chem3D Pro 12.0 was utilized to produce 3D structures of the ligands (KA [reference substance] and analogs **1**, **5**, **7**, and **9**) through energy minimization. The binding affinities between the ligands and mTYR were supplied by Chimera 1.19 and AutoDock Vina, while the possible chemical interactions between mTYR and the ligands were supplied by LigandScout 4.4.8 (http://www.inteligand.com/ligandscout/download.shtml, accessed on 3 February 2025) (Cambridge, UK). Prior to running the docking simulation between the NBTC analogs and mTYR, tropolone, an original ligand of 2Y9X, was redocked into mTYR for validation. According to redocking results, the tropolone had a binding energy of −5.5 kcal/mol and could replicate the binding posture (Supplementary Materials S57). The co-crystallized and experimental poses had a root mean square deviation of 0.61 Å, suggesting that the docking simulation could be conducted correctly.

### 2.14. In Vitro ROS Scavenging Assay [51,52]

Esterase (1.4 units/mL; Sigma-Aldrich, St. Louis, MO, USA) in 50 mM phosphate buffer (pH 7.4) was mixed with 2.5 mM 2′,7′-dichlorodihydrofluorescein diacetate (DCFH-DA) (Molecular Probes, Eugene, OR, USA), incubated at 37 °C for 0.5 h, and kept on ice in the dark until use. Phosphate buffer (pH 7.4; 180 μL) and the test sample’s DMSO solution (final concentration: 40 μM; 10 μL; analogs **1**–**10** and Trolox [positive control]) were dispensed into each well of a black 96-well plate containing SIN-1 (final concentration: 10 μM; 10 μL, Sigma-Aldrich, St. Louis, MO, USA) in DMSO, and the plate was kept in the dark for 20 min. The DCFH-DA-esterase mixture (50 μL) was added to each well. Fluorescence of each well was measured using a fluorescence microplate reader (Berthold Advances GmbH & Co., Bad Wildbad, Germany) at an excitation wavelength of 485 nm and an emission wavelength of 535 nm. Significance was confirmed in three independent experiments.

### 2.15. Melanin Formation and Cellular TYR Activity Assay with or Without Cu^2+^ Ions

In a 6-well plate, B16F10 cells (5 × 10^3^ cells/well) were cultured for 24 h. The cultured cells were treated with test samples (analogs **9** [1.25, 2.5, and 5 μM]) with or without CuSO_4_ (Sigma-Aldrich, St. Louis, MO, USA) solution (5 μM in distilled water) for 1 h and then exposed to stimulators (IBMX plus α-MSH; 200 and 1 μM, respectively). The subsequent experimental procedures were identical to those used for the cellular TYR activity and melanin content assays in B16F10 cells.

### 2.16. Statistical Analysis

The statistical analyses were carried out with GraphPad Prism version 5 software (La Jolla, CA, USA). The mean ± standard errors of the mean (SEM) was used to represent all results. The significance of intergroup differences was assessed using the Newman-Keuls test and a one-way analysis of variance. *p*-values < 0.05 were considered statistically significant.

## 3. Results and Discussion

### 3.1. Synthesis

**Scheme 1:**
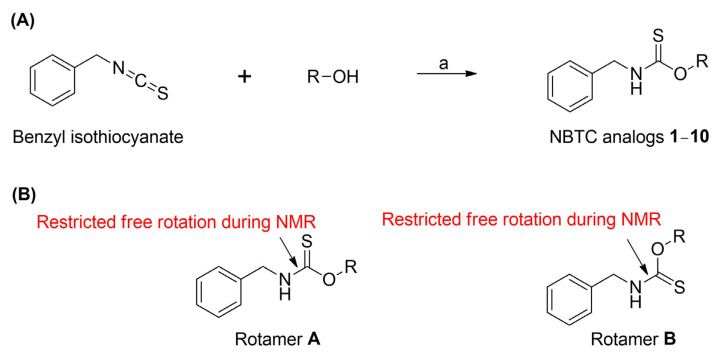
(**A**) Synthetic scheme for *N*-benzylthiocarbamate (NBTC) analogs **1**–**10**. Reagents and conditions: (a) ROH used as a solvent and reagent: methanol for **1**, ethanol for **2**, 1-propanol for **3**, 2-propanol for **4**, and 1-butanol for **5**, reflux, 2–3 days, 80–99%, or ROH (1.0 equiv.) as a reagent: 1-pentanol for **6**, isoamyl alcohol for **7**, 4-phenyl-1-butanol for **8**, cyclopentanol for **9**, and cyclohexanol for **10**, toluene, 100 °C, 2–3 days, 39–77%. (**B**) Structures of two rotamers, **A** and **B**.

The target compounds, NBTC analogs **1**–**10**, were prepared by reacting benzyl isothiocyanate with appropriate alcohols without a catalyst (Scheme 1A). Using ^13^C and ^1^H nuclear magnetic resonance (NMR) spectroscopy, the structures of **1**–**10** were identified, and the structures of **1**–**3** and **10** were reaffirmed by comparing them with the NMR data published in the literature [35,37]. ^1^H and ^13^C NMR spectra of all NBTC analogs showed that they exist as two rotamers, **A** and **B** (Scheme 1B). This is consistent with the literature [35] report by Breme et al. that thiocarbamates present two rotamer forms in NMR. These results seemed to be caused by limited free rotation around the N-C bond of the thiocarbamate functional group during NMR. The ratio between the major and minor rotamers ranged from 1.86:1 to 1.38:1 [35].

### 3.2. Pyrocatechol Violet (PCV) Reagent-Based Cu^2+^ Chelation Activity

Reagents that chelate with Cu^2+^ ions include bathocuproinedisulfonic acid (BCDS) [53,54], *N*,*N*′-disalicylidene-2,3-diaminopyridine [55], and PCV [38]. BCDS and *N*,*N*′-disalicylidene-2,3-diaminopyridine exhibit maximum absorbance at 490 and 414 nm, respectively, after binding to Cu^2+^ ions. However, because of the high selectivity of PCV for Cu^2+^ ions and the advantage of being able to directly measure the concentration of Cu^2+^ ion without a complex pretreatment process, PCV was used to screen the Cu^2+^ ion chelation of analogs. A PCV reagent and CuSO_4_ were used to examine the Cu^2+^ chelation activity of NBTC analogs **1**–**10**. The PCV reagent contains two components that can chelate Cu^2+^ ions: 2-hydroxycyclohexa-2,5-dienone (which is structurally similar to KA, a known Cu^2+^ chelator [56]) and catechol. When the PCV reagent solution chelated Cu^2+^ ions, it strongly absorbed the maximum absorption wavelength of 632 nm, resulting in a bluish-violet color [57]. KA was used as a positive control. CuSO_4_ (209 μM) and PCV (80 μM) were used with all test samples at 100 μM.

**Figure 3:**
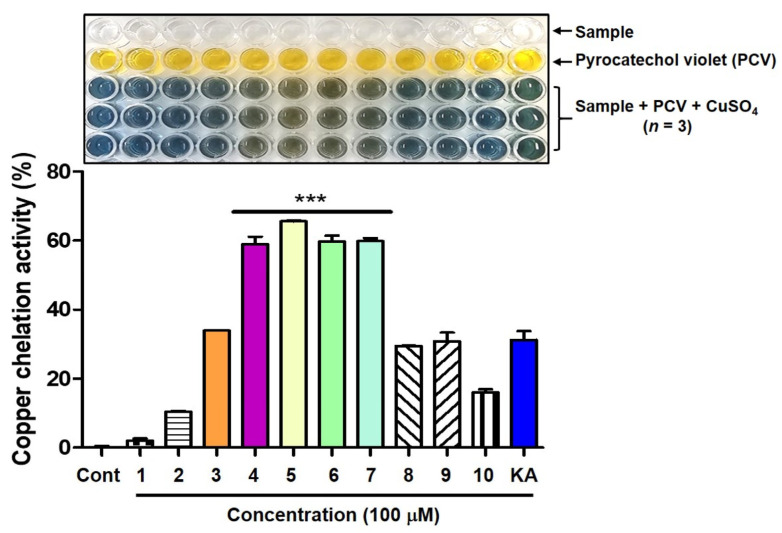
Cu^2+^ chelation activity of NBTC analogs **1**–**10** determined using copper sulfate (209 μM) and pyrocatechol violet (PCV; 80 μM). The activities of their Cu^2+^ chelation were contrasted with those of kojic acid (20 μM; KA). All test samples were tested at 100 μM. *** *p* < 0.001 vs. KA-treated group.

The Cu^2+^ chelation activity of KA was 31% (Figure 3). While the Cu^2+^ chelation activity of analogs **1**, **2**, and **10** was lower than that of KA, ranging from 2 to 16%, analogs **3**, **8**, and **9** showed Cu^2+^ chelation activity comparable to that of KA, ranging from 30 to 34%. Within the range of 59–66%, four NBTC analogs, **4**–**7**, demonstrated significantly higher Cu^2+^ chelation activity than KA. The number of carbons in the *O*-alkyl moiety of the thiocarbamates and the shape (ring or linear) of the *O*-alkyls affected the Cu^2+^ chelation activity of the NBTC analogs. Reduced Cu^2+^ chelation activity was observed in analogs with fewer carbons in the *O*-alkyl moiety of thiocarbamate than in those with more carbons: analogs **1**, **2**, and **10** bearing 1–3 carbons in the *O*-alkyl moiety compared with analogs **4**–**9** bearing 4 or more carbons in the *O*-alkyl moiety. Compared with analogs with an *O*-alkyl that consisted of a ring, those with a linear *O*-alkyl moiety in thiocarbamates demonstrated greater Cu^2+^ chelation activity: analogs **4**–**7** compared with analogs **8** and **9**. PTU also exhibits strong Cu^2+^ chelation activity. According to published literature [58], KA and PTU exhibited Cu^2+^ chelation activities of 30% and 40%, respectively, in experiments using PCV. Compared with these results, analogs **4**–**7** have Cu^2+^ chelation activities similar to or greater than PTU.

### 3.3. mTYR Activity Inhibition

With the expectation of positive results, the inhibitory potency of NBTC analogs on mTYR activity was assessed using KA as a positive control because most of them demonstrated strong Cu^2+^ chelation activity. With l-tyrosine and l-DOPA as the substrates, TYR inhibition activity was assessed at three distinct concentrations of analogs **1**–**10** (100, 20, and 4 μM for l-DOPA and 50, 10, and 2 μM for l-tyrosine). KA was used at concentrations of 50, 10, and 2 μM for both substrates.

Table 1 shows the percentage inhibition and IC_50_ values of analogs **1**–**10** and KA. The percentage inhibitions were obtained at 50 μM for l-tyrosine and 100 μM for l-DOPA. In the presence of l-tyrosine, the strongest mTYR inhibitory activity was demonstrated by NBTC analogs **1**, **3**, and **7**, which contained *O*-methyl, *O*-propyl, and *O*-4-phenylbutyl groups, respectively. However, their percentage inhibitions (24–29% inhibition) were much lower than those of KA (92.4 ± 0.1% inhibition; IC_50_ value: 22.1 ± 0.6 μM). With inhibitions of less than 21%, the remaining NBTC analogs suppressed mTYR activity. KA demonstrated the best mTYR inhibitory activity, even when l-DOPA was used as the substrate. At 50 μM, it inhibited mTYR activity by 85%, with an IC_50_ value of 20.3 ± 0.2 μM. The most potent mTYR inhibitory activity was demonstrated by analogs **1**, **5**, **7**, and **9**, which contained *O*-methyl, *O*-pentyl, *O*-4-phenylbutyl, and *O*-cyclohexyl groups, respectively. These analogs had IC_50_ values of 65 ± 2, 78 ± 3, 70 ± 5, and 80 ± 2 μM (Table 1 and Supplementary Materials S28–S31), and at 100 μM, they inhibited mTYR activity by 67, 63, 68, and 60%, respectively. At 100 μM, the remaining NBTC analogs exhibited weak mTYR inhibitory activity, with inhibition rates below 35%. No discernible relationship between structural alterations in the *O*-alkyl moiety and mTYR inhibitory activity was found, regardless of the type of substrate employed. Although most NBTC analogs demonstrated Cu^2+^ chelation activity that was on par with or even stronger than that of KA, these results indicated that the NBTC analogs did not inhibit mTYR activity as effectively as KA. Similar results have been reported for 5,6,7,8-tetrahydro-4*H*-furo [3,2-*c*]azepine-4-thione (T4FAT), which exhibits significantly superior Cu^2+^ chelation activity compared to KA in experiments using PCV, but exhibits similar mTYR inhibitory activity [59]. To determine whether this discrepancy also holds true for other TYRs, the intracellular TYR inhibitory activity of the NBTC analogs was evaluated in B16F10 cells.

### 3.4. Cytotoxicity of NBTC Analogs in B16F10 Cells

**Figure 4:**
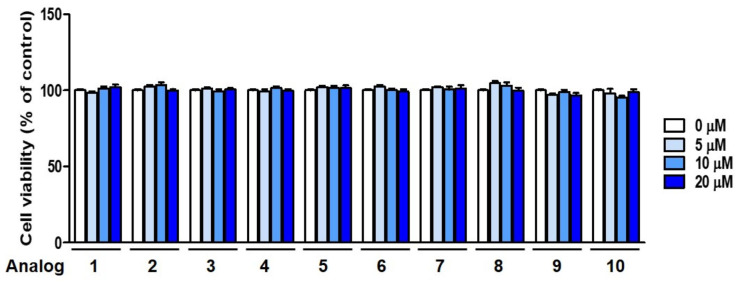
Seventy-two-hour cell viability of **1**–**10** on B16F10 cells. At 20, 10, 5, and 0 μM, analogs were employed. Cell viability was determined using an EZ-Cytox kit.

NBTC analogs were tested for cell viability in B16F10 cells before their effects on TYR activity in B16F10 cells were examined. After B16F10 cells were treated with NBTC analogs (20, 10, and 5 μM) for 72 h, cytotoxicity was assessed using an EZ-Cytox kit. Figure 4 depicts the outcomes of the cell viability tests. At all the concentrations tested, none of the NBTC analogs exhibited cytotoxicity. Thus, NBTC analogs were tested at concentrations (≤20 μM) that did not cause cytotoxicity to investigate their effects on melanin contents and cellular TYR activity in B16F10 cells.

### 3.5. Effect of NBTC Analogs on Melanin Content Level in B16F10 Cells

Prior to assessing the effect of NBTC analogs on cellular TYR activity, the effect on melanin content production in B16F10 cells was investigated. For the experiments on melanin production in cells, ten analogs, **1**–**10**, were used at 20 μM. Prior to being exposed to stimulators comprising 3-isobutyl-1-methylxanthine (IBMX; 200 μM) and alpha-melanocyte-stimulating hormone (α-MSH; 1 μM), B16F10 cells were exposed to test samples (KA [20 μM; positive material] and **1**–**10**) for 1 h. The optical density (OD) was assessed at 405 nm to determine melanin content following a 72 h incubation period.

**Figure 5:**
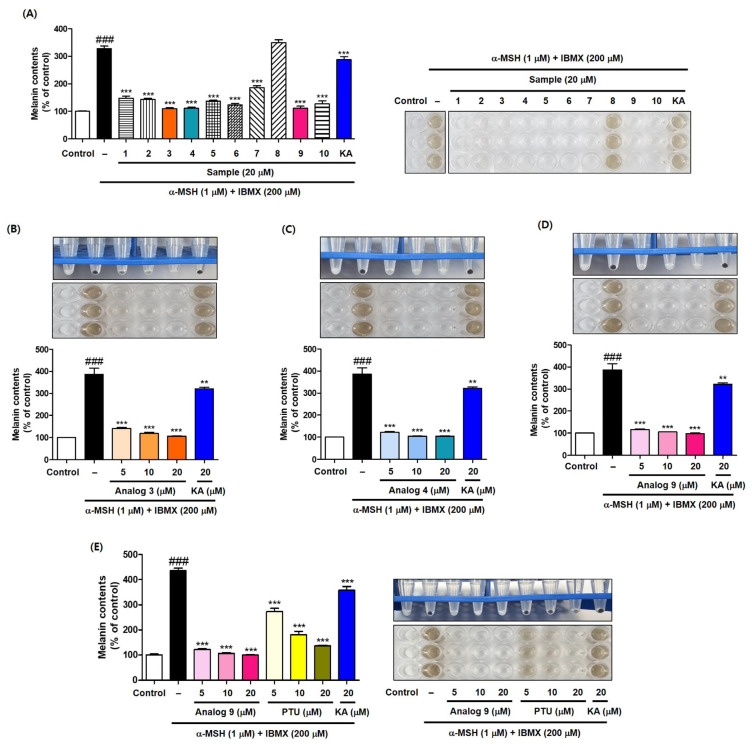
Impact of **1**–**10** on B16F10 cell melanin production. (**A**) Results of melanin production at 20 μM of **1**–**10**. (**B**–**E**) Results of melanin production at three concentrations (20, 10, and 5 μM) of analogs **3** (**B**), **4** (**C**), and **9** (**D**,**E**). Phenylthiourea (PTU; 20, 10, and 5 μM) and kojic acid (KA; 20 μM) were used for comparing anti-melanogenic activity. One hour before the B16F10 cells were exposed to stimulators (IBMX [200 μM] plus α-MSH [1 μM]), test samples were administered to the cells. The antimelanogenic effects of the test samples were assessed by measuring their absorbances at 405 nm following a 72 h incubation period. α-MSH, α-melanocyte-stimulating hormone; IBMX, 3-isobutyl-1-methylxanthine. *** *p* < 0.001 and ** *p* < 0.01 vs. stimulator-treated group; ### *p* < 0.001 vs. control.

Compared with the untreated control group, the stimulators significantly increased melanin content (Figure 5A). The increased melanin content induced by the stimulators was significantly reduced by treatment with KA (TYR inhibitor). The degree to which the NBTC analogs reduced stimulator-induced melanin content was greater than that of KA, except for **8** with *O*-cyclopentyl. Most of the analogs diminished the melanin content to nearly the control level. These results are in contrast to those in T4FAT [59]. T4FAT, which has stronger Cu^2+^ chelating activity than KA, has been reported to have a 20-fold lower melanin production inhibitory activity in B16F10 cells. For concentration-dependent melanin production experiments, the three analogs, **3**, **4**, and **9**, that most potently inhibited melanin production were selected. Prior to being exposed to the stimulators (α-MSH [1 μM] plus IBMX [200 μM]) for 72 h, B16F10 cells were exposed to three concentrations (5, 10, and 20 μM) of these three analogs for 1 h. PTU (20, 10, and 5 μM) and KA (20 μM) were employed as positive controls.

Compared with the untreated control, B16F10 cells exposed to the stimulators produced a significant amount of melanin; however, the stimulator-induced melanin content was significantly reduced when KA was administered (Figure 5B–E). Figure 5B–D shows that the melanin content levels increased by stimulator treatment were significantly and concentration-dependently decreased by NBTC analogs **3**, **4**, and **9**. Their inhibitory efficacy of melanin production at a concentration of 5 μM was also much higher than that of 20 μM KA. All analogs exerted highly potent anti-melanogenesis effects, and at 5 μM, their melanin content levels dropped to the control group’s levels. At each of the three tested concentrations, analog **9** demonstrated noticeably greater inhibition of melanin production than PTU (Figure 5E). In B16F10 cells, the NBTC analogs demonstrated strong melanogenesis-inhibitory potency, although they did not strongly inhibit mTYR activity. Therefore, we aimed to ascertain whether this strong antimelanogenic effect was due to the inhibition of B16F10 cellular TYR activity.

### 3.6. Assessment of the Impact of NBTC Analogs on TYR Inhibitory Activity in B16F10 Cells

Three distinct concentrations of 20, 10, and 5 μM of NBTC analogs **3**, **4**, and **9**, which demonstrated potent antimelanogenic effects, were employed to assess the inhibition of cellular TYR activity in B16F10 cells. B16F10 cells were pre-treated with test samples 1 h prior to stimulator treatment (α-MSH plus IBMX; 1 and 200 μM, respectively), similar to that in the melanin production experiments. The absorbances at 475 nm were used to measure the cellular TYR inhibitory activity after 72 h of incubation, and 20 μM KA was utilized as a positive material.

**Figure 6:**
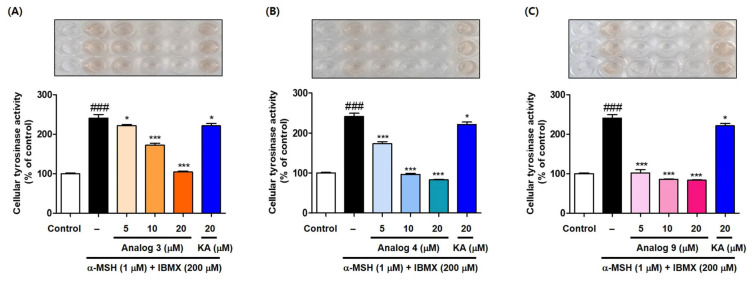
Effect of NBTC analogs **3** (**A**), **4** (**B**), and **9** (**C**) on B16F10 cellular TYR activity. TYR activity was compared using 20 μM kojic acid (KA). Tests were conducted on NBTC analogs at 20, 10, and 5 μM. One hour before the B16F10 cells were exposed to stimulators (IBMX [200 μM] plus α-MSH [1 μM]), test samples were administered to the cells. The cellular TYR activities of the analogs were assessed based their absorbances at 475 nm following a 72 h incubation period. *** *p* < 0.001 and * *p* < 0.05 vs. stimulator-treated group, ### *p* < 0.001 vs. control.

All the tested analogs had stronger TYR inhibitory activity than KA and significantly decreased cellular TYR activity (Figure 6). Additionally, these analogs concentration-dependently inhibited TYR activity. The strongest inhibitory potency on cellular TYR activity was shown by analog **9**, which at 5 μM was significantly more effective than 20 μM KA as a B16F10 cellular TYR inhibitor. To the best of our knowledge, these NBTC analogs are the first thiocarbamate-functionalized TYR inhibitors. These findings are consistent with those of the melanogenesis experiment, suggesting that the anti-TYR activity of the NBTC analogs is primarily responsible for their melanogenesis-inhibiting effects.

Melanosomes, organelles found in melanocytes, contain a glycosylated monomeric enzyme known as mammalian TYR. mTYR is a non-glycosylated tetrameric enzyme found in the cytosol [60,61,62]. Additionally, mammalian TYR is anchored to the melanosome membrane. Moreover, mammalian TYR and mTYR share only 22–24% identity, with an amino acid sequence of 48–49% [63]. The distinct TYR inhibitory activity of the NBTC analogs of mTYR and B16F10 TYR was most likely caused by structural and locational differences between mTYR and mammalian TYR. The log *p* values of the NBTC analogs ranged from 2.43 to 5.27, whereas those of KA and PTU were −2.45 and 0.73, respectively, based on measurements made using ChemDraw Ultra 12.0 software (CambridgeSoft Corporation, Cambridge, MA, USA). These findings suggest that NBTC analogs have greater lipophilicity than KA and PTU. According to the report by Masuri et al. [64], lipophilicity also appears to be an important factor in regulating anti-TYR activity. Thus, we hypothesized that the superior lipophilicity of the NBTC analogs over KA and PTU was at least partially responsible for their high activity in suppressing cellular TYR and melanin synthesis in B16F10 cells.

Analogs **4**–**7** were more potent Cu^2+^-chelating agents than analog **9**, according to the Cu^2+^ chelation results shown in Figure 3. However, the experimental findings on cellular TYR activity and melanin content in B16F10 cells, as displayed in Figure 5 and Figure 6, demonstrated that **9** was an equipotent or more potent TYR inhibitor in B16F10 cells than **4**–**7**. This discrepancy may have been because the amino acid residues surrounding the active site affect the Cu^2+^-chelating capacity of the TYR active site. It is believed that analog **9** possesses a molecular form that can chelate Cu^2+^ ions of B16F10 cell TYR more effectively than analogs **4**–**7** within the active site of B16F10 cell TYR, even though analogs **4**–**7** were found to have better Cu^2+^-chelating ability than analog **9**. Collectively, these findings suggest that Cu^2+^-chelation results may not always reflect TYR activity inhibition.

Many current studies rely on mTYR to identify TYR inhibitors. Because of the structural differences (glycosylation, subunit, and their localization) between mTYR and mammalian TYR, developing mammalian TYR inhibitors based on mTYR inhibition results is problematic: Compounds that inhibit mTYR activity may not inhibit mammalian TYR activity, and vice versa.

### 3.7. Impact of NBTC Analogs on the In Situ Cellular TYR Activity in B16F10 Cells

To verify that the NBTC analogs suppress B16F10 cellular TYR activity, an in situ cellular TYR activity method was used to test the capacity of analogs **3**, **4**, and **9** to suppress cellular TYR activity in B16F10 cells. Analogs were given at 20, 10, and 5 μM, and in situ cellular TYR activity was compared using KA (20 μM). Before B16F10 cells were exposed to stimulators (IBMX [200 μM] and α-MSH [1 μM]), test samples (**3**, **4**, and **9** and KA) were given to the cells 1 h beforehand. After a 72 h incubation period, l-DOPA was administered to B16F10 cells for 2 h, and images were captured using a camera. CS analyzer software was used to quantify the pigmentation areas in the cell photographs.

**Figure 7:**
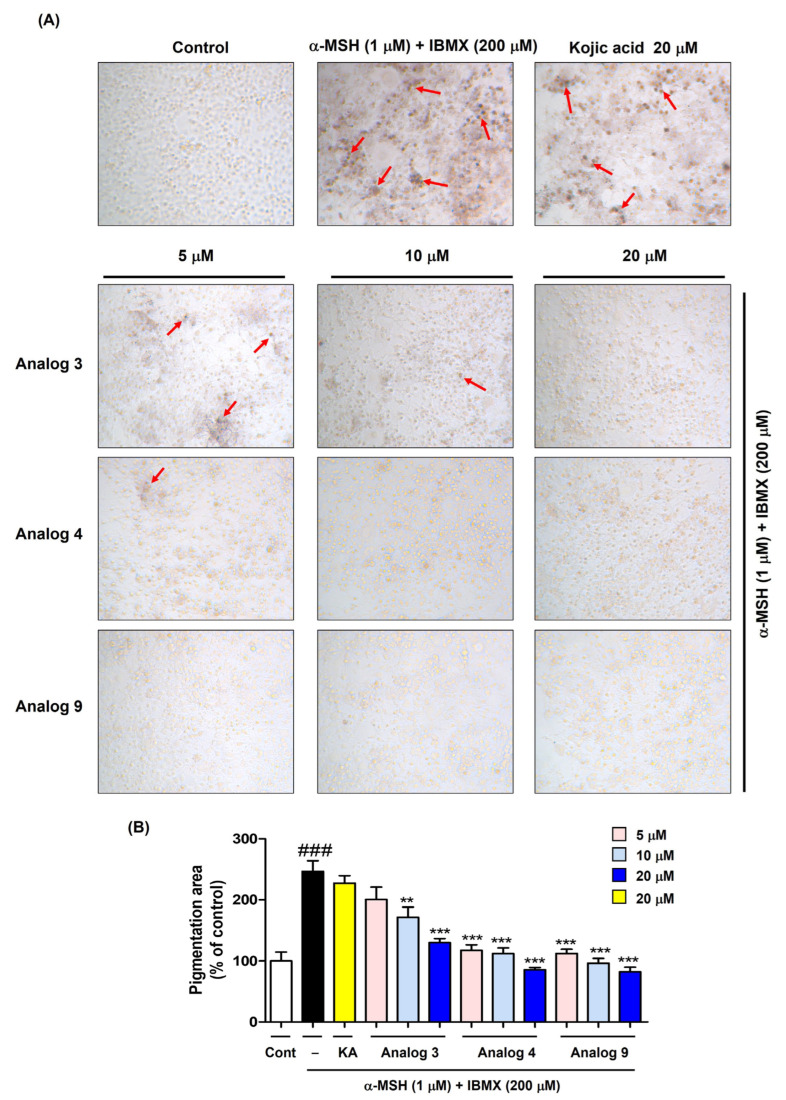
Analogs **3**, **4**, and **9** and kojic acid (KA) in situ B16F10 cellular TYR activity results. (**A**) Images and (**B**) pigmentation areas of the stained cells. KA and analogs were administered at 20 and 20, 10, and 5 μM, respectively. One hour before the B16F10 cells were treated with stimulators (IBMX [200 μM] plus α-MSH [1 μM]), test samples were administered to the cells. After 72 h of incubation, l-DOPA staining was applied to B16F10 cells for 2 h. Melanin-stained cells were quantified using CS Analyzer image analysis software after photographing with a camera. Arrows indicate cells heavily stained with melanin. ### *p* < 0.001 vs. control, ** *p* < 0.01 and *** *p* < 0.001 vs. group treated with stimulators.

The in situ cellular TYR activity outcomes are shown in Figure 7. The number of cells that were strongly stained with melanin rose in response to stimulator exposure, while the number of cells that were strongly stained decreased in response to KA treatment. When administered at three different concentrations, NBTC analogs **3**, **4**, and **9** significantly and dose-dependently decreased the number of strongly stained cells. In situ cellular TYR activity was more strongly inhibited by analogs **4** and **9** than by analog **3**. Despite having the lowest in situ cellular TYR activity among the tested analogs, analog **3** inhibited in situ cellular TYR activity more effectively than KA (20 μM), even at 5 μM. The in situ cellular TYR activity levels of analogs **4** and **9** at 20 μM were 86% and 82% lower, respectively, than the controls (100%). These findings suggest that, in contrast to mTYR activity, NBTC analogs strongly inhibit B16F10 mammalian TYR activity.

### 3.8. Impact of NBTC Analogs on Potato Juice Browning

**Figure 8:**
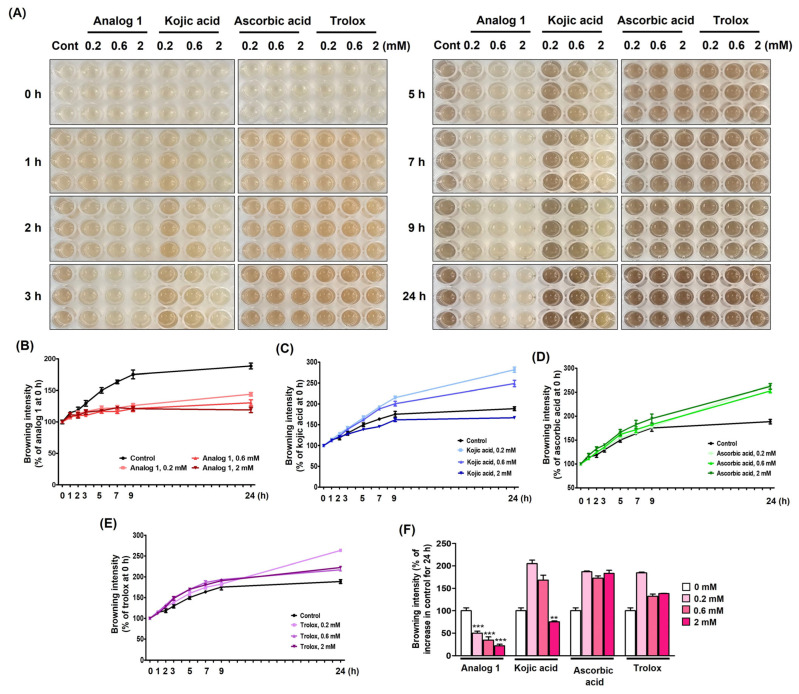
Effect of NBTC analog **1** on the browning of potato juice. Trolox, ascorbic acid, and kojic acid were employed for comparing activity. Every test sample was examined for 24 h at 0.2, 0.6, and 2 mM. (**A**) Photographs of the potato juice browning phenomenon were obtained at 0, 1, 2, 3, 5, 7, 9, and 24 h. Changes in browning intensity of potato juice over time are shown when analog **1** (**B**), kojic acid (**C**), ascorbic acid (**D**), and Trolox (**E**) were added. (**F**) The degree of increase in browning for analog **1**, kojic acid, ascorbic acid, and Trolox at 24 h was compared with that of browning increase in the control group. *** *p* < 0.001 and ** *p* < 0.01 vs. control.

Browning is a type of oxidation process. Since TYR is an important enzyme involved in the browning process of crops such as fruits and vegetables, we evaluated the effect of NBTC analogs on the browning of potato juice. Analog **1**, which demonstrated the strongest anti-browning effect in the preliminary experiment with 0.2 mM NBTC analogs (Supplementary Materials; S49), was chosen for the main studies on the browning of potato juice. Over 24 h, the effect of analog **1** on potato juice browning was investigated at 0.2, 0.6, and 2.0 mM. Ascorbic acid, Trolox, and KA were used to compare the activities. Regardless of the presence of the test samples, the browning of potato juice progressively increased over time (Figure 8A–E). The 24 h browning of potato juice by the test compounds (analog **1**, KA, ascorbic acid, and Trolox) was compared with that of the control (100%) (Figure 8F). Ascorbic acid and Trolox did not reduce but rather increased the browning of potato juice at all concentrations tested. In contrast, KA enhanced the browning in potato juice at low concentrations (0.2 and 0.6 mM) but decreased the browning of potato juice to 75% at high concentration (2 mM). A potent concentration-dependent anti-browning effect was demonstrated by NBTC analog **1**. At 0.2, 0.6, and 2 mM, analog **1** decreased the browning of potato juice by 50, 34, and 21%, respectively. These findings suggest that analog **1** has a much greater effect on preventing the browning of potato juice than the tested positive controls and that analog **1** exhibits strong anti-browning effect even at low concentrations where the positive controls do not exhibit anti-browning effects.

*p*-Coumaric acid, a common secondary metabolite of plants, inhibits browning when semi-purified enzymes are used, but in fresh potato juice, it enhances browning [65]. The literature suggests that the increased browning is likely due to its role as an alternative substrate for TYR and the involvement of non-substrate components in potato juice in the post-TYR reaction sequences. In contrast, NBTC analog **1** probably does not act as a substrate for TYR and thus effectively inhibits browning in fresh potato juice.

### 3.9. Depigmentation on Zebrafish Larvae

In B16F10 cells, NBTC analogs demonstrated strong antimelanogenic activity and strong inhibition of mammalian TYR activity, despite their weak inhibition of mTYR activity. Furthermore, some of these analogs successfully prevented the browning of potato juice. The zebrafish larval depigmentation effects of NBTC analogs were evaluated using zebrafish embryos to examine whether these compounds could inhibit melanogenesis in vivo [66,67]. Following the initial zebrafish larval depigmentation tests with NBTC analogs at 0.1 mM, four analogs (**1**–**3**, and **10**) were selected for primary zebrafish larval depigmentation tests at three distinct concentrations (0.01, 0.03, and 0.1 mM). For activity comparison, 0.01, 0.03, and 0.1 mM of PTU and 20 mM of KA were used as positive controls. PRONASE^®^ was utilized to remove the chorion of zebrafish embryos at 24 h post-fertilization (hpf). At 28 hpf, the test samples (**1**–**3** and **10**, PTU, and KA) were applied for 44 h. The pigment product of zebrafish larvae was assessed at 72 hpf by capturing images of the larvae in both dorsal and lateral views using an SMZ745T stereomicroscope. Colored areas were calculated utilizing the CS analyzer 3.0.

**Figure 9:**
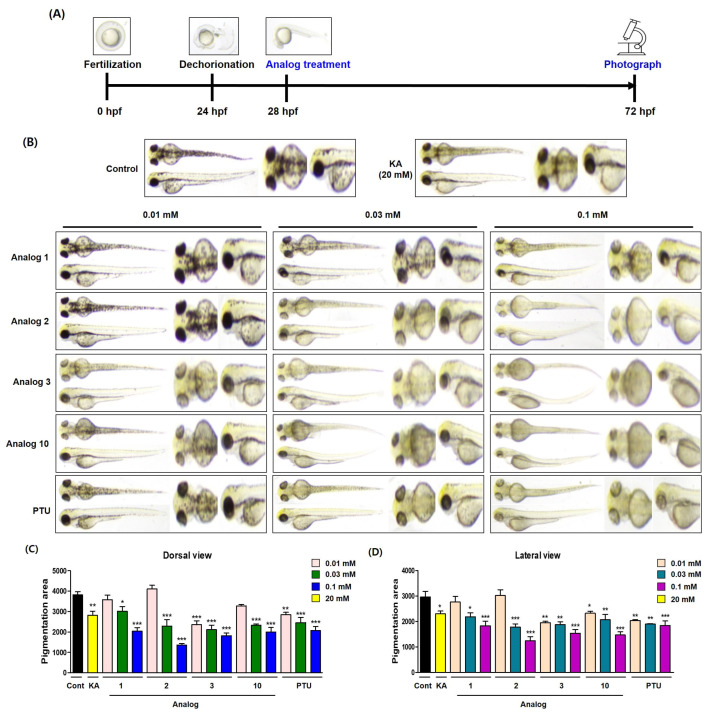
Depigmenting effect of NBTC analogs on zebrafish larvae. (**A**) A timeline of experimental treatments. (**B**) Images obtained 44 h following the administration of test samples (kojic acid [KA; 20 mM], phenylthiourea [PTU; 0.01, 0.03, and 0.1 mM], and analogs **1**, **2**, **3**, and **10** [0.01, 0.03, and 0.1 mM]). Dorsal (**C**) and lateral (**D**) views are used to measure the pigmentation areas. *** *p* < 0.001, ** *p* < 0.01, and * *p* < 0.05 vs. control.

At 72 hpf, zebrafish larvae exposed to KA exhibited comparatively weak depigmentation, whereas those in the control group exhibited strong pigmentation (Figure 9). All tested samples, including PTU, demonstrated a concentration-dependent inhibition mechanism and were more effective inhibitors of zebrafish larval pigmentation than KA (Figure 9B–D). Among the NBTC analogs, analog **3** exhibited the best pigment removal efficacy at 0.01 mM, whereas analog **2** exhibited the best efficacy at the highest measured concentration (0.1 mM). Furthermore, at 0.01 mM, analog **3** was more effective than PTU at inhibiting zebrafish larval pigmentation (Figure 9C), and at 0.1 mM, all three analogs (**2**, **3**, and **10**) were more effective than PTU at doing so (Figure 9C,D). Particularly, analog **2** demonstrated depigmentation potency that was considerably greater than that of PTU at 0.1 mM, and analog **3** demonstrated depigmentation efficacy that was considerably greater than that of KA, even at a concentration of 0.01 mM, which is 2000 times lower than that of KA (Figure 9C,D). All tested NBTC analogs demonstrated depigmentation potency considerably better than that of KA in the zebrafish larvae depigmentation experiment, with some analogs demonstrating depigmentation potency even stronger than that of PTU. The depigmentation potency of these NBTC analogs was superior to that of PFRMY [68], a recently reported Cu^2+^ chelating peptide, and ellagic acid [69], a Cu^2+^ ion chelator. Although NBTC analogs only slightly reduced the activity of mTYR (Table 1), they significantly reduced the mammalian TYR activity in B16F10 cells (Figure 6), demonstrating a strong inhibitory effect on the ability of the cells to produce melanin. Additionally, in contrast to the mTYR inhibition results, NBTC analogs showed potent depigmentation results in experiments using zebrafish embryos. These outcomes were probably attributed to structural variations among various TYRs.

### 3.10. Inhibitory Mechanism of NBTC Analogs **1**, **5**, **7**, and **9** Against mTYR

The mechanism of TYR inhibition by the NBTC analogs was investigated using mTYR. To create Lineweaver–Burk (L–B) plots for kinetic studies, the four analogs (**1**, **5**, **7**, and **9**) that showed the best mTYR inhibitory activity in the presence of l-DOPA were utilized. Three distinct concentrations of each analog (80, 40, and 20 μM) and five to six distinct concentrations of the substrate (l-DOPA) (16, 8, 4, 2, and 1 mM or 16, 8, 4, 2, 1, and 0.5 mM) were used.

**Figure 10:**
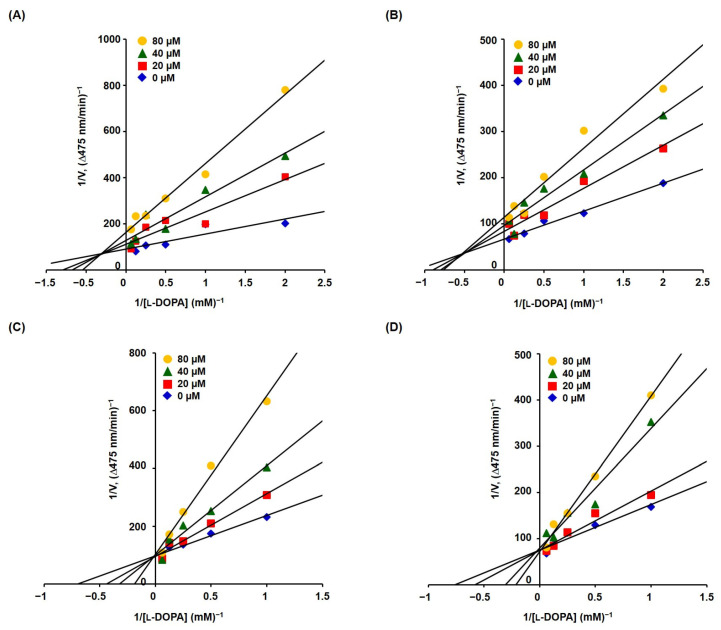
Plots of the Lineweaver–Burk obtained by measuring the initial dopachrome production velocity using mTYR with **1** (**A**), **5** (**B**), **7** (**C**), and **9** (**D**). The substrate (l-DOPA) was used at 16, 8, 4, 2, and 1 mM or 16, 8, 4, 2, 1, and 0.5 mM, and each NBTC analog was used at 80, 40, 20, and 0 μM.

Figure 10 shows L–B plots produced by graphing the reciprocal of the substrate concentration versus the reciprocal of the initial rate of dopachrome generation in the existence of each NBTC analog. Four distinct lines merged at a point in the second quadrant of the L–B plots for analogs **1** and **5**, whereas four distinct lines merged at a point on the y-axis of the L–B plots for analogs **7** and **9**. These characteristics suggested that analogs **7** and **9** were competitive mTYR inhibitors with a constant maximum enzyme reaction rate independent of the inhibitor concentration, whereas analogs **1** and **5** were mixed-type mTYR inhibitors.

**Figure 11:**
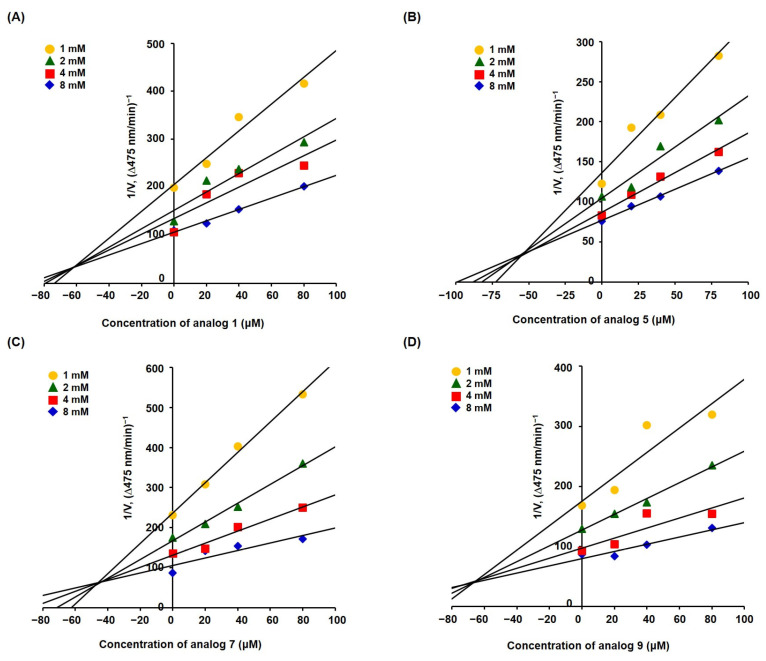
Dixon plots produced by measuring the initial dopachrome production velocity using mTYR with **1** (**A**), **5** (**B**), **7** (**C**), and **9** (**D**). The substrate (l-DOPA) was used at 8, 4, 2, and 1 mM, and each NBTC analog was used at 80, 40, 20, and 0 μM.

Dixon plots were created using the kinetic data used for the L–B plots (Figure 11) to calculate the inhibition constant (K_i_), which shows how firmly a compound binds to a target protein, an enzyme, or a receptor. Four distinct straight lines were generated in each Dixon plot and joined at a point in the second quadrant. The reciprocal of the substrate concentration corresponding to the x-axis of the L–B plot was changed to the inhibitor concentration and replotted to obtain Dixon plots. Four distinct straight lines were generated in each Dixon plot and joined at a point in the second quadrant. The K_i_ value of each analog is represented by the absolute value of the x-coordinate of the converged point. The K_i_ values of four analogs, **1**, **5**, **7**, and **9**, ranged from 4.5 × 10^−5^ to 6.7 × 10^−5^ M, indicating that the K_i_ values of NBTC analogs were much higher than those (from 1.7 × 10^−8^ to 2.7 × 10^−8^ M) of 2-thiobenzothiazole (2-TBT) derivatives, which were reported as Cu^2+^ chelators and strongly inhibited mTYR activity [70]. These results suggest that, unlike 2-TBT derivatives, NBTC analogs do not bind strongly to the Cu^2+^ ions of mTYR and therefore do not exhibit mTYR inhibitory activity as strong as that of 2-TBT derivatives.

### 3.11. mTYR-Based In Silico Docking Simulation of NBTC Analogs

To support the kinetic results obtained, NBTC analogs **1**, **5**, **7**, and **9** were used in docking simulations with mTYR, as they demonstrated the strongest inhibitory activity against mTYR. The three-dimensional (3D) X-ray co-crystal structure of the complex (PDB ID: 2Y9X) obtained from tropolone (a ligand) and mTYR (*Agaricus bisporus*) was used to ascertain the binding energy between mTYR and these NBTC analogs. ChemDraw Ultra 12.0 was used to draw the two-dimensional (2D) structures of the analogs, and Chem3D Pro 12.0 was used to convert the 2D analog structures into their corresponding 3D structures. After the water molecules and tropolone were removed from the X-ray co-crystal structure of mTYR, AutoDock Vina 1.2.0 was utilized to dock the X-ray mTYR structure with the 3D analog structures. For comparison of binding affinity, KA was utilized.

**Figure 12:**
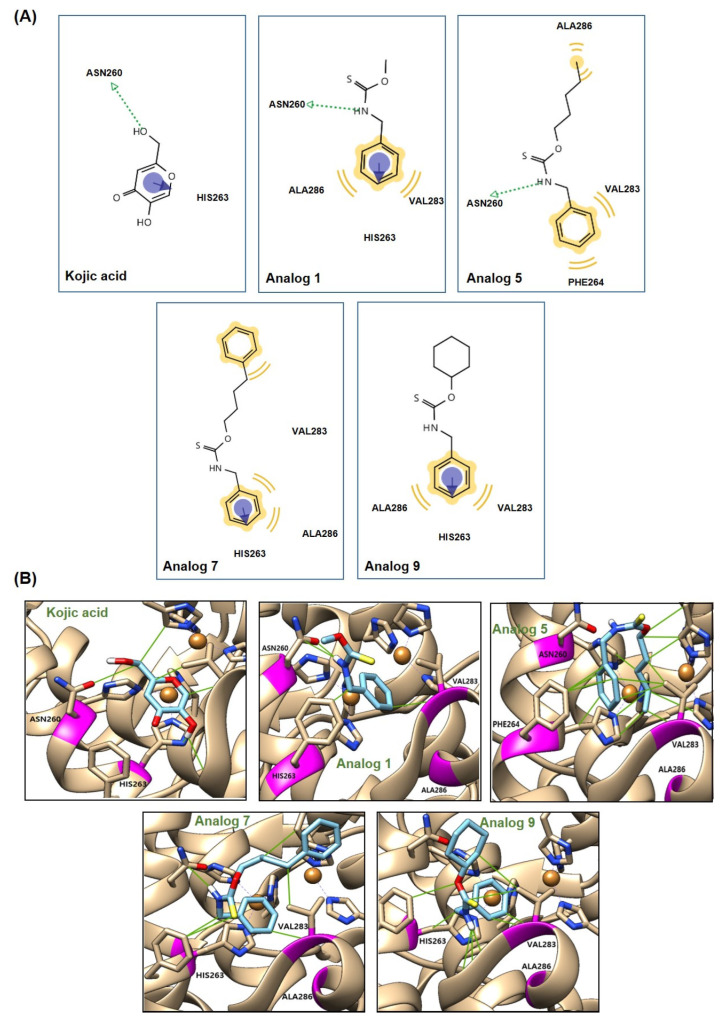
Potential chemical interactions between mTYR (PDB ID: 2Y9X) and NBTC analogs **1**, **5**, **7**, and **9** and kojic acid, which were bound to the mTYR active site. AutoDock Vina and LigandScout were utilized to obtain the results of docking simulations and potential chemical interactions, respectively. Two-dimensional (**A**) and three-dimensional (**B**) images. The yellow wave, green arrow, and blue arrow in (**A**) stand for hydrophobic interaction, a hydrogen bond, and pi-pi stacking, respectively.

The mTYR active site was strongly bound by KA and analogs **1**, **5**, **7**, and **9**, which had binding energies of −5.4, −5.6, −5.7, −6.7, and −6.4 kcal/mol, respectively. All the binding energies of the analogs were lower than those of KA. In the active site pocket, KA formed a hydrogen bond with Asn260 and a pi-pi stacking interaction with His263 (Figure 12). Analog **1** interacted with the same amino acids as KA, generating a hydrogen bond and pi-pi stacking interaction. Furthermore, the phenyl ring of analog **1** and two amino acids, Ala286 and Val283, formed two hydrophobic interactions with each other. Analog **5** formed hydrophobic interactions with Ala286, Val283, and Phe264 and a hydrogen bond with Asn260. In analog **5**, the phenyl ring and pentyl moiety participated in hydrophobic interactions. Hydrophobic and pi-pi stacking interactions were observed in analog **7** with two phenyl rings. Of the two phenyl rings, only the phenyl ring of the benzyl group showed pi-pi stacking interaction with His263. Ala286 exhibited only hydrophobic interactions with the phenyl ring of the benzyl group, whereas Val283 formed hydrophobic interactions with both phenyl rings. In analog **9**, the phenyl ring of the benzyl moiety engaged in hydrophobic interactions with Ala286 and Val283 and a pi-pi stacking interaction with His263. Because they participated in the hydrophobic interactions that led to their binding to the mTYR active site of all NBTC analogs, Val283 and Ala286 were believed to be crucial for the inhibition of the mTYR activity of NBTC analogs. Asn260 was involved in forming hydrogen bonds in analogs **1** and **5**, while His263 was involved in pi-pi stacking interactions in analogs **1**, **7**, and **9**. These findings suggest that His263 and Asn260 are also important for inhibiting the mTYR activity of NBTC analogs. Two amino acids, His263 and Asn260, have been reported to line the mTYR active site, and several in silico studies have shown that these amino acids interact with inhibitors in a similar manner to that mentioned above [71,72,73].

The binding energies of analogs **1**–**10** and the pictures of possible chemical interactions between analogs **2**–**4**, **6**, **8**, and **10** and mTYR amino acid residues are shown in Supplementary Materials S58. There was no strong correlation between the docking scores and the observed mTYR inhibitory activity. However, docking results of analogs **2**–**4**, **6**, **8**, and **10** also showed that the key interactions are the pi-pi stacking interaction between the benzyl ring of the NBTC analogs and His263, as well as the hydrophobic interactions with Ala286 and Val283.

**Figure 13:**
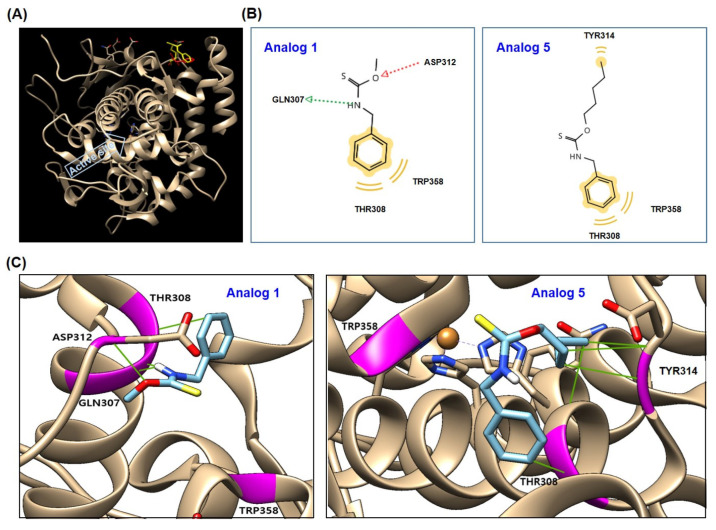
Potential chemical interactions between mTYR (PDB ID: 2Y9X) and NBTC analogs **1** and **5** found in the mTYR allosteric site. LigandScout and AutoDock Vina were used to obtain the results of the possible chemical interactions and the in silico docking simulation, respectively. A representation of a rounded ribbon and two- and three-dimensional images are shown in (**A**–**C**). Analogs **1** and **5** are displayed in red and yellow, respectively, in (**A**). In (**B**), the red and green arrows and yellow wave represent hydrogen bond acceptors, donors, and hydrophobic interactions, respectively.

Consistent with the kinetic results, analogs **1** and **5** were also found to bind to the allosteric site of mTYR. Having binding energies of −6.1 and −5.2 kcal/mol, respectively, these analogs bound to the same allosteric site. As illustrated in Figure 13, analog **1** displayed hydrophobic interactions with Thr308 and Trp358 and two hydrogen bonds with Gln307 and Asp312. The oxygen atom and amino (NH) group of the thiocarbamate functional group served as hydrogen bond acceptors and donors, respectively. Only the hydrophobic interactions between the pentyl moiety and Tyr314 and between the phenyl ring and Thr308 and Trp358 were generated by analog **5**. Thr308 and Trp358 are considered crucial for mTYR allosteric site binding, as they are involved in the binding of analogs **1** and **5**.

Results from docking simulations at the mTYR active and allosteric sites supported that **1** and **5** are mixed-type inhibitors, while **7** and **9** are competitive inhibitors.

### 3.12. ROS Scavenging Activity

Because ROS play a certain role in melanogenesis, scavenging ROS through antioxidant activity may reduce melanogenesis [21,23,74,75]. Therefore, the effect of NBTC analogs on ROS scavenging was investigated using an in vitro ROS scavenging assay. The principle of measuring the ROS scavenging ability of the compounds is as follows. 2′,7′-Dichlorodihydrofluorescein diacetate (DCFH-DA) is a fluorescent dye used to measure ROS levels. DCFH-DA is hydrolyzed by esterase to produce 2′,7′-dichlorodihydrofluorescein (DCFH). When treated with 3-morpholinosydnonimine (SIN-1), which generates ROS, DCFH is oxidized by the generated ROS to the fluorescent substance 2′,7′-dichlorofluorescein (DCF). Therefore, ROS levels were calculated by measuring the fluorescence of DCF at 530 nm, and the ROS-scavenging ability of the test samples (NBTC analogs and Trolox [positive material]) was determined.

**Figure 14:**
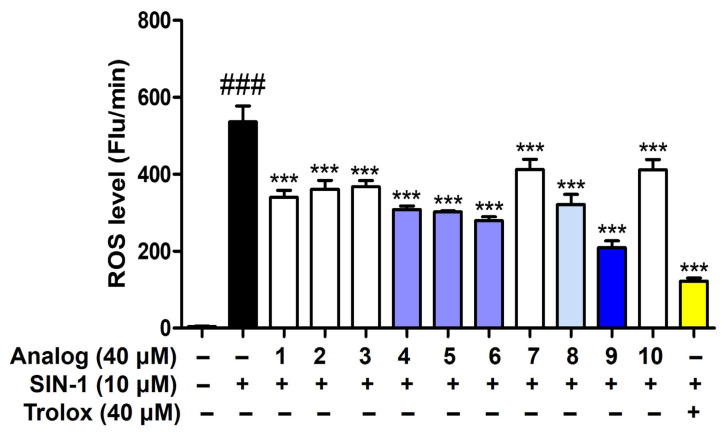
Effects of NBTC analogs **1**–**10** on ROS scavenging. Trolox (TR; positive control) and NBTC analogs were tested at a concentration of 40 μM, and SIN-1, used as a ROS generator, was used at a concentration of 10 μM. *** *p* < 0.001 vs. SIN-1-treated group; ### *p* < 0.001 vs. control.

The SIN-1-treated group showed high ROS levels (Figure 14). Although Trolox exhibited the highest ROS-scavenging activity, all groups exposed to NBTC analogs significantly reduced SIN-1-induced ROS levels. In particular, analog **9**, which demonstrated the most potent inhibitory efficacy against cellular tyrosinase and melanin production in B16F10 cells, exhibited the most potent ROS-scavenging effect among the tested analogs. These results suggest that the ROS-scavenging activity of NBTC analogs contributed, at least in part, to their inhibitory effects on melanin production.

### 3.13. Cytotoxicity in Hs27 and HaCaT Cells

An EZ-Cytox kit was used to assess how the NBTC analogs affect the cytotoxicity of Hs27 (fibroblasts) and HaCaT (keratinocytes) cells. For 24 h, Hs27 and HaCaT cells were treated with analogs **1**–**10** (20, 10, and 5 μM). Cell viability was assessed following a 2 h exposure to EZ-Cytox solution.

**Figure 15:**
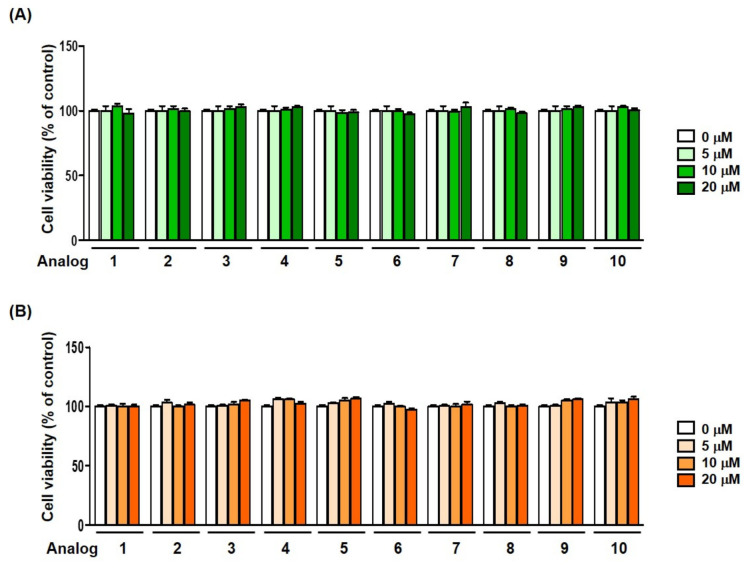
Cell viabilities of NBTC analogs **1**–**10** on HaCaT (keratinocyte cell line) (**A**) and Hs27 (fibroblast cell line) (**B**) cells. Both cells were exposed to an EZ-Cytox solution after being exposed to **1**–**10** at 20, 10, and 5 μM for 24 h. Absorbance was measured at 450 nm to determine cell viability after 2 h of incubation.

The viability results for each cell type are shown in Figure 15. Up to 20 μM, none of the NBTC analogs exhibited any discernible cytotoxicity in either cell line. Given that the predominant cell types in the dermis and epidermis are fibroblasts and keratinocytes, these findings imply that the NBTC analogs may be safe for the skin.

### 3.14. Effect of Cu^2+^ Ions on the Ability of Analog **9** to Inhibit Cellular TYR Activity and Melanin Production in B16F10 Cells

The strongest inhibitory effects on B16F10 cell TYR activity and melanogenesis were observed for analog **9**. The effects of Cu^2+^ ions on melanin synthesis and TYR activity in B16F10 cells were examined with analog **9**. The effect of CuSO_4_ on B16F10 cell viability was assessed before the experiment. Since the results showed that CuSO_4_ did not affect B16F10 cell viability up to 5 μM for 72 h (Supplementary Materials: S54), B16F10 cells were treated with 1.25, 2.5, and 5 μM of **9** with or without CuSO_4_ (5 μM) 1 h prior to the treatment with stimulators (IBMX and α-MSH; 200 and 1 μM, respectively) and then cultivated for 72 h. Melanin levels and cellular TYR activity were measured.

**Figure 16:**
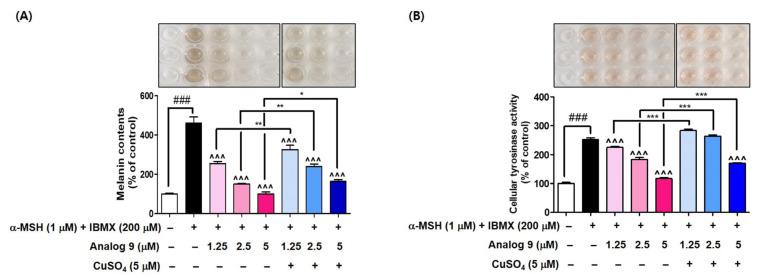
Impact of CuSO_4_ on the capacity of NBTC analog **9** to suppress melanin synthesis (**A**) and B16F10 cellular TYR activity (**B**). Analog **9** (1.25, 2.5, and 5 μM) with or without CuSO_4_ (5 μM) was administered to B16F10 cells 1 h prior to the stimulator treatment (IBMX [200 μM] plus α-MSH [1 μM]). Melanin levels and cellular TYR activity were assessed following a 72 h incubation period. ^^^ *p* < 0.001 vs. group treated with stimulators; *** *p* < 0.001, ** *p* < 0.01, and * *p* < 0.05 vs. analog **9**-treated group without added CuSO_4_; ### *p* < 0.001 vs. control.

Melanin levels and cellular TYR activity were significantly increased by the stimulator exposure (Figure 16). Regardless of whether CuSO_4_ was present, treatment with analog **9** dose-dependently and significantly diminished the stimulator-induced melanin levels. In the presence of CuSO_4_, however, the effect of analog **9** to suppress melanin biosynthesis was significantly reduced at all concentrations. In the 5 μM group without CuSO_4_, the melanin level was reduced to the control group level; however, in the 5 μM group with CuSO_4_, it increased to 1.6 times the level of the control group. Similar outcomes were observed in the cellular TYR activity experiment, as in the melanin content experiment; CuSO_4_ significantly attenuated the cellular TYR inhibition potency of **9** at all concentrations tested. These findings imply that exogenously supplied CuSO_4_ contributes to chelation with NBTC analog **9** in B16F10 cells, thereby decreasing cellular TYR inhibitory activity and, in turn, melanin production, which supports the idea that analog **9** serves as a Cu^2+^ chelator in B16F10 cells.

The difference between the inhibition of mTYR and B16F10 cellular TYR by NBTC analogs likely stems from differences in interactions with amino acid residues in the active site of each TYR, in addition to the Cu^2+^ chelation effect. Furthermore, the ROS-inhibiting activity of NBTC analogs may also contribute in part to the inhibition of B16F10 cellular TYR.

### 3.15. Study Limitations

Based on the positive results above, we plan to address the following issues in future follow-up studies: (1) expanding the limited exploration of SAR through structural diversification of analogs, (2) studying inhibitory activity in purified human TYR, and (3) studying the correlation between the inhibition of potato juice browning of analogs and potato TYR activity by purifying potato TYR in our laboratory.

## 4. Conclusions

Alkyl NBTC analogs **1**–**10** were synthesized as a new class of potent TYR inhibitors that chelate Cu^2+^ ions. Analogs **3**–**7** were more effective Cu^2+^ chelators than KA. Most of the analogs showed stronger inhibitory effects on melanin formation in B16F10 cells than KA, although they did not strongly inhibit mTYR activity. The antimelanogenic effect in B16F10 cells of **3**, **4**, and **9** resulted from their cellular TYR inhibitory activity. In the browning experiment with potato juice, analog **1** demonstrated strong inhibitory activity, and in the depigmentation experiment with zebrafish larvae, analogs **1**–**3** and **10** also demonstrated strong inhibitory activity. Experiments comparing the inhibitory effects of analog **9** on TYR activity and melanin production in B16F10 cells with and without the exogenous addition of Cu^2+^ ions demonstrated that analog **9** functions as a Cu^2+^ chelator in B16F10 cells. Additionally, their ROS scavenging ability is thought to have contributed at least partially to their antimelanogenic effect.

## Figures and Tables

**Table 1:** Inhibition percentages and IC_50_ values of alkyl *N*-benzylthiocarbamate (NBTC) analogs **1**–**10** against mushroom tyrosinase in the presence of l-tyrosine and l-DOPA.

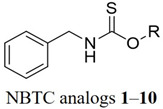			
Analog	R	% Inhibition	
		l-Tyrosine	l-DOPA
**1**	Methyl	26 ± 1 ***	66.7 ± 0.6 *** (65 ± 2 ***)
**2**	Ethyl	19 ± 3 ***	35 ± 3 ***
**3**	Propyl	24.1 ± 0.8 ***	22 ± 2 ***
**4**	Butyl	10.7 ± 0.9 ***	30 ± 1 ***
**5**	Pentyl	20.8 ± 0.2 ***	63 ± 2 *** (78 ± 3 ***)
**6**	Isopentyl	9.8 ± 0.8 ***	31.4 ± 0.8 ***
**7**	4-Phenylbutyl	29 ± 3 ***	68 ± 4 *** (70 ± 5 ***)
**8**	Cyclopentyl	9 ± 3 ***	32 ± 3 ***
**9**	Cyclohexyl	15 ± 3 ***	60 ± 2 *** (80 ± 2 ***)
**10**	Isopropyl	1.7 ± 0.4 ***	18.0 ± 0.8 ***
Kojic acid		92.4 ± 0.1 (22.1 ± 0.6)	84.5 ± 0.7 ^a^ (20.3 ± 0.2)

The mean ± SEM of triplicates at concentrations of 50 μM for l-tyrosine and 100 μM for l-DOPA were used to display the inhibition (%) values. All data are presented as the mean ± standard error of the mean (SEM) of three independent replicate experiments, and IC_50_ values (half-maximal inhibition concentration; μM) are shown in parentheses. ^a^ The value was obtained at 50 μM. *p* *** < 0.001 vs. kojic acid. l-DOPA, l-3,4-dihydroxyphenylalanine.

## Data Availability

Data is contained within the article or Supplementary Materials.

## Supplementary Materials

The following supporting information can be downloaded at https://www.mdpi.com/article/10.3390/antiox15010039/s1. Figure S1: ^1^H NMR spectrum of analog **1**; Figure S2: ^13^C NMR spectrum of analog **1**; Figure S3: ^1^H NMR spectrum of analog **2**; Figure S4: ^13^C NMR spectrum of analog **2**; Figure S5: ^1^H NMR spectrum of analog **3**; Figure S6: ^13^C NMR spectrum of analog **3**; Figure S7: ^1^H NMR spectrum of analog **4**; Figure S8: ^13^C NMR spectrum of analog **4**; Figure S9: ^1^H NMR spectrum of analog **5**; Figure S10: ^13^C NMR spectrum of analog **5**; Figure S11: ^1^H NMR spectrum of analog **6**; Figure S12: ^13^C NMR spectrum of analog **6**; Figure S13: ^1^H NMR spectrum of analog **7**; Figure S14: ^13^C NMR spectrum of analog **7**; Figure S15: ^1^H NMR spectrum of analog **8**; Figure S16: ^13^C NMR spectrum of analog **8**; Figure S17: ^1^H NMR spectrum of analog **9**; Figure S18: ^13^C NMR spectrum of analog **9**; Figure S19: ^1^H NMR spectrum of analog **10**; Figure S20: ^13^C NMR spectrum of analog **10**; Figure S21: HRMS spectrum of analog **4**; Figure S22: HRMS spectrum of analog **5**; Figure S23: HRMS spectrum of analog **6**; Figure S24: HRMS spectrum of analog **7**; Figure S25: HRMS spectrum of analog **8**; Figure S26: HRMS spectrum of analog **9**; Figure S27: Original data (A) and photo (B) for Cu^2+^ chelating activity of NBTC analogs **1**–**10**; Figure S28: Graphs used to calculate IC_50_ values for kojic acid in the presence of l-tyrosine; Figure S29: Graphs used to calculate IC_50_ values for analog **1** and **5** in the presence of l-dopa; Figure S30: Graphs used to calculate IC_50_ values for analog **7** and **9** in the presence of l-dopa; Figure S31: Graphs used to calculate IC_50_ values for kojic acid in the presence of l-dopa; Figure S32: Photo (A) and original data (B) for melanin production results at 20 μM of **1**–**10** in B16F10 cells; Figure S33: Melanin production results at three different concentrations (5, 10, and 20 μM) of analogs **3** (A), **4** (B), and **9** (C); Figure S34: Photo (A) and original data (B) for melanin production results for analog **9** and PTU (positive control) in B16F10 cells; Figure S35: Effect of NBTC analogs on cellular tyrosinase activity in B16F10 cells. Cellular tyrosinase activity results at three different concentrations (5, 10, and 20 μM) of analogs **3** (A), **4** (B), and **9** (C); Figure S36: Images of the control group (*n* = 5) in the in situ B16F10 cellular tyrosinase activity experiments; Figure S37: Images of α-MSH + IBMX group (*n* = 5) in the in situ B16F10 cellular tyrosinase activity experiments; Figure S38: Images of kojic acid (20 μM) group (*n* = 7) in the in situ B16F10 cellular tyrosinase activity experiments; Figure S39: Images of analog **3** (5 μM) group (*n* = 10) in the in situ B16F10 cellular tyrosinase activity experiments; Figure S40: Images of analog **3** (10 μM) group (*n* = 10) in the in situ B16F10 cellular tyrosinase activity experiments; Figure S41: Images of analog **3** (20 μM) group (*n* = 7) in the in situ B16F10 cellular tyrosinase activity experiments; Figure S42: Images of analog **4** (5 μM) group (*n* = 8) in the in situ B16F10 cellular tyrosinase activity experiments; Figure S43: Images of analog **4** (10 μM) group (*n* = 7) in the in situ B16F10 cellular tyrosinase activity experiments; Figure S44: Images of analog **4** (20 μM) group (*n* = 7) in the in situ B16F10 cellular tyrosinase activity experiments; Figure S45: Images of analog **9** (5 μM) group (*n* = 9) in the in situ B16F10 cellular tyrosinase activity experiments; Figure S46: Images of analog **9** (10 μM) group (*n* = 8) in the in situ B16F10 cellular tyrosinase activity experiments; Figure S47: Images of analog **9** (20 μM) group (*n* = 9) in the in situ B16F10 cellular tyrosinase activity experiments; Figure S48: Pigmentation area data (A) and analysis graphs (B) for l-dopa staining of analogs **3**, **4**, and **9**; Figure S49: Effect of NBTC analogs **1**–**10** on the browning of potato juice; Figure S50: Depigmentation results performed using zebrafish embryos. Dorsal and lateral views of zebrafish larvae treated with control (A) and KA (kojic acid, 20 mM); Figure S51: Depigmentation results performed using zebrafish embryos. Dorsal and lateral views of zebrafish larvae treated with analog **1** (0.01, 0.03 and 0.1 mM) (A) and analog **2** (0.01, 0.03 and 0.1 mM) (B); Figure S52: Depigmentation results performed using zebrafish embryos. Dorsal and lateral views of zebrafish larvae treated with analog **3** (0.01, 0.03 and 0.1 mM) (A) and analog **10** (0.01, 0.03 and 0.1 mM) (B); Figure S53: Depigmentation results performed using zebrafish embryos. Dorsal and lateral views of zebrafish larvae treated with PTU (0.01, 0.03 and 0.1 mM); Figure S54: Cell viability of CuSO_4_ on B16F10 cells; Figure S55: Photo (A) and original data (B) of melanin production results for analog **9** with or without CuSO_4_ in B16F10 cells; Figure S56: Photo (A) and original data (B) of cellular TYR activity results for analog **9** with or without CuSO_4_ in B16F10 cells; Figure S57: Alignment of the re-docked ligand (green) and co-crystallized ligand (red) with the 2Y9X protein; Figure S58. Docking scores of analogs **1**–**10** and possible chemical interactions between analogs **2**–**4**, **6**, **8**, and **10** and mTYR amino acid residues.

## Abbreviations

α-MSH	α-Melanocyte-stimulating hormone
DPBS	Dulbecco’s phosphate-buffered saline
Hpf	Hour post-fertilization
IBMX	3-Isobutyl-1-methylxanthine
L–B	Lineweaver–Burk
mTYR	Mushroom tyrosinase
NBTC	*N*-Benzylthiocarbamate
NMR	Nuclear magnetic resonance
OD	Optical density
PTU	*N*-Phenylthiourea
PCV	Pyrocatechol violet
ROS	Reactive oxygen species
TRP	TYR-related protein
TYR	Tyrosinase
VM	VersaMax^®^ microplate
