# Supplementary material for: Alkyl N-Benzylthiocarbamates, the First Copper(II) Ion-Chelating Tyrosinase Inhibitors with a Thiocarbamate Group and ROS-Scavenging Activity, Exhibit Different Inhibitory Activities Depending on the Origin of Tyrosinase
Source: Antioxidants (Basel). 2025 Dec 26;15(1):39. doi: 10.3390/antiox15010039 (PMC12837751; doi:10.3390/antiox15010039)
Supplement: Supplementary file 1 [file antioxidants-15-00039-s001.zip › antioxidants-4028886-supplementary.pdf]

Article

Alkyl *N*-Benzylthiocarbamates, the First Copper(II) Ion-Chelating Tyrosinase Inhibitors Bearing a Thiocarbamate Group, Exhibit Different Inhibitory Activities Depending on the Origin of Tyrosinase

Hee Jin Jung [1,†], Hyeon Seo Park [1,†], Yeonsoo Jeong [1], Ga Young Kim [1], Hyunju Lee [1], Hye Soo Park [1], Hye Jin Kim [1], Hyunhee Ju [1], Hyejin Kang [1], Yujin Park [2], Hae Young Chung [1] and Hyung Ryong Moon [1,*]

[1] College of Pharmacy and Research Institute for Drug Development, Pusan National University, Busan 46241, Republic of Korea; hjjung2046@pusan.ac.kr (H.J.J.); gustj6956@pusan.ac.kr (H.S.P.); jysoo627@pusan.ac.kr (Y.J.); kgy9905@pusan.ac.kr (G.Y.K.); lhj6384770@pusan.ac.kr (H.L.); hyesoo0713@pusan.ac.kr (H.S.P.); khj3358@pusan.ac.kr (H.J.K.); hyunh@pusan.ac.kr (H.J.); dirgowls22@pusan.ac.kr (H.K.); hyjung@pusan.ac.kr (H.Y.C.)

[2] Department of Medicinal Chemistry, New Drug Development Center, Daegu-Gyeongbuk Medical Innovation Foundation, Daegu 41061, Republic of Korea; pyj1016@kmedihub.re.kr

* Correspondence: mhr108@pusan.ac.kr; Tel.: +82-51-510-2815; Fax: +82-51-513-6754

† These authors contributed equally to this work.

Supporting Information

For

Contents

S1. ^{1}H NMR spectrum of analog **1** 6

S2. ^{13}C NMR spectrum of analog **1** 7

S3. ^{1}H NMR spectrum of analog **2** 8

S4. ^{13}C NMR spectrum of analog **2** 9

S5. ^{1}H NMR spectrum of analog **3** 10

S6. ^{13}C NMR spectrum of analog **3** 11

S7. ^{1}H NMR spectrum of analog **4** 12

S8. ^{13}C NMR spectrum of analog **4** 13

S9. ^{1}H NMR spectrum of analog **5** 14

S10. ^{13}C NMR spectrum of analog **5** 15

S11. ^{1}H NMR spectrum of analog **6** 16

S12. ^{13}C NMR spectrum of analog **6** 17

S13. ^{1}H NMR spectrum of analog **7** 18

S14. ^{13}C NMR spectrum of analog **7** 19

S15. ^{1}H NMR spectrum of analog **8** 20

S16. ^{13}C NMR spectrum of analog **8** 21

S17. ^{1}H NMR spectrum of analog **9** 22

S18. ^{13}C NMR spectrum of analog **9** 23

S19. ^{1}H NMR spectrum of analog **10** 24

S20. ^{13}C NMR spectrum of analog **10** 25

S21. HRMS spectrum of analog **4**..........26

S22. HRMS spectrum of analog **5**..........27

S23. HRMS spectrum of analog **6**..........28

S24. HRMS spectrum of analog **7**..........29

S25. HRMS spectrum of analog **8**..........30

S26. HRMS spectrum of analog **9**..........31

S27. Original data (A) and photo (B) for Cu^{2+} chelating activity of NBTC analogs **1–10** 32

S28. Graphs used to calculate IC_{50} values for kojic acid in the presence of L-tyrosine 33

S29. Graphs used to calculate IC_{50} values for analog **1** and **5** in the presence of L-dopa 34

S30. Graphs used to calculate IC_{50} values for analog **7** and **9** in the presence of L-dopa 35

S31. Graphs used to calculate IC_{50} values for kojic acid in the presence of L-dopa 36

S32. Photo (A) and original data (B) for melanin production results at 20 μM of **1**–**10** in B16F10 cells 37

S33. Melanin production results at three different concentrations (5, 10, and 20 μM) of analogs **3** (A), **4** (B), and **9** (C) 38

S34. Photo (A) and original data (B) for melanin production results for analog **9** and PTU (positive control) in B16F10 cells 39

S35. Effect of NBTC analogs on cellular tyrosinase activity in B16F10 cells. Cellular tyrosinase activity results at three different concentrations (5, 10, and 20 μM) of analogs **3** (A), **4** (B), and **9** (C) 40

S36. Images of the control group (*n* = 5) in the in situ B16F10 cellular tyrosinase activity experiments 41

S37. Images of α-MSH + IBMX group (*n* = 5) in the in situ B16F10 cellular tyrosinase activity experiments 42

S38. Images of kojic acid (20 μM) group (*n* = 7) in the in situ B16F10 cellular tyrosinase activity experiments 43

S39. Images of analog **3** (5 μM) group (*n* = 10) in the in situ B16F10 cellular tyrosinase activity experiments 44

S40. Images of analog **3** (10 μM) group (*n* = 10) in the in situ B16F10 cellular tyrosinase activity experiments 45

S41. Images of analog **3** (20 μM) group (*n* = 7) in the in situ B16F10 cellular tyrosinase activity experiments 46

S42. Images of analog **4** (5 μM) group (*n* = 8) in the in situ B16F10 cellular tyrosinase activity experiments 47

S43. Images of analog **4** (10 μM) group (*n* = 7) in the in situ B16F10 cellular tyrosinase activity experiments 48

S44. Images of analog **4** (20 μM) group (*n* = 7) in the in situ B16F10 cellular tyrosinase activity experiments 49

S45. Images of analog **9** (5 μM) group (*n* = 9) in the in situ B16F10 cellular tyrosinase activity experiments 50

S46. Images of analog **9** (10 μM) group (*n* = 8) in the in situ B16F10 cellular tyrosinase activity experiments 51

S47. Images of analog **9** (20 μM) group (*n* = 9) in the in situ B16F10 cellular tyrosinase activity experiments 52

S48. Pigmentation area data (A) and analysis graphs (B) for L-dopa staining of analogs **3**, **4**, and **9** 53

S49. Effect of NBTC analogs **1**–**10** on the browning of potato juice.. 54

S50. Depigmentation results performed using zebrafish embryos. Dorsal and lateral views of zebrafish larvae treated with control (A) and KA (kojic acid, 20 mM) 55

S51. Depigmentation results performed using zebrafish embryos. Dorsal and lateral views of zebrafish larvae treated with analog **1** (0.01, 0.03 and 0.1 mM) (A) and analog **2** (0.01, 0.03 and 0.1 mM) (B) 56

S52. Depigmentation results performed using zebrafish embryos. Dorsal and lateral views of zebrafish larvae treated with analog **3** (0.01, 0.03 and 0.1 mM) (A) and analog **10** (0.01, 0.03 and 0.1 mM) (B) 57

S53. Depigmentation results performed using zebrafish embryos. Dorsal and lateral views of zebrafish larvae treated with PTU (0.01, 0.03 and 0.1 mM) 58

S54. Cell viability of $CuSO_4$ on B16F10 cells 59

S55. Photo (A) and original data (B) of melanin production results for analog **9** with or without $CuSO_4$ in B16F10 cells 60

S56. Photo (A) and original data (B) of cellular TYR activity results for analog **9** with or without $CuSO_4$ in B16F10 cells 61

S57. Alignment of the re-docked ligand (green) and co-crystallized ligand (red) with the 2Y9X protein……………………………………………………………………….……..62

S58. Docking scores of analogs **1**–**10** and possible chemical interactions between analogs **2**–**4**, **6**, **8**, and **10** and mTYR amino acid residues…………………..……………………….……63

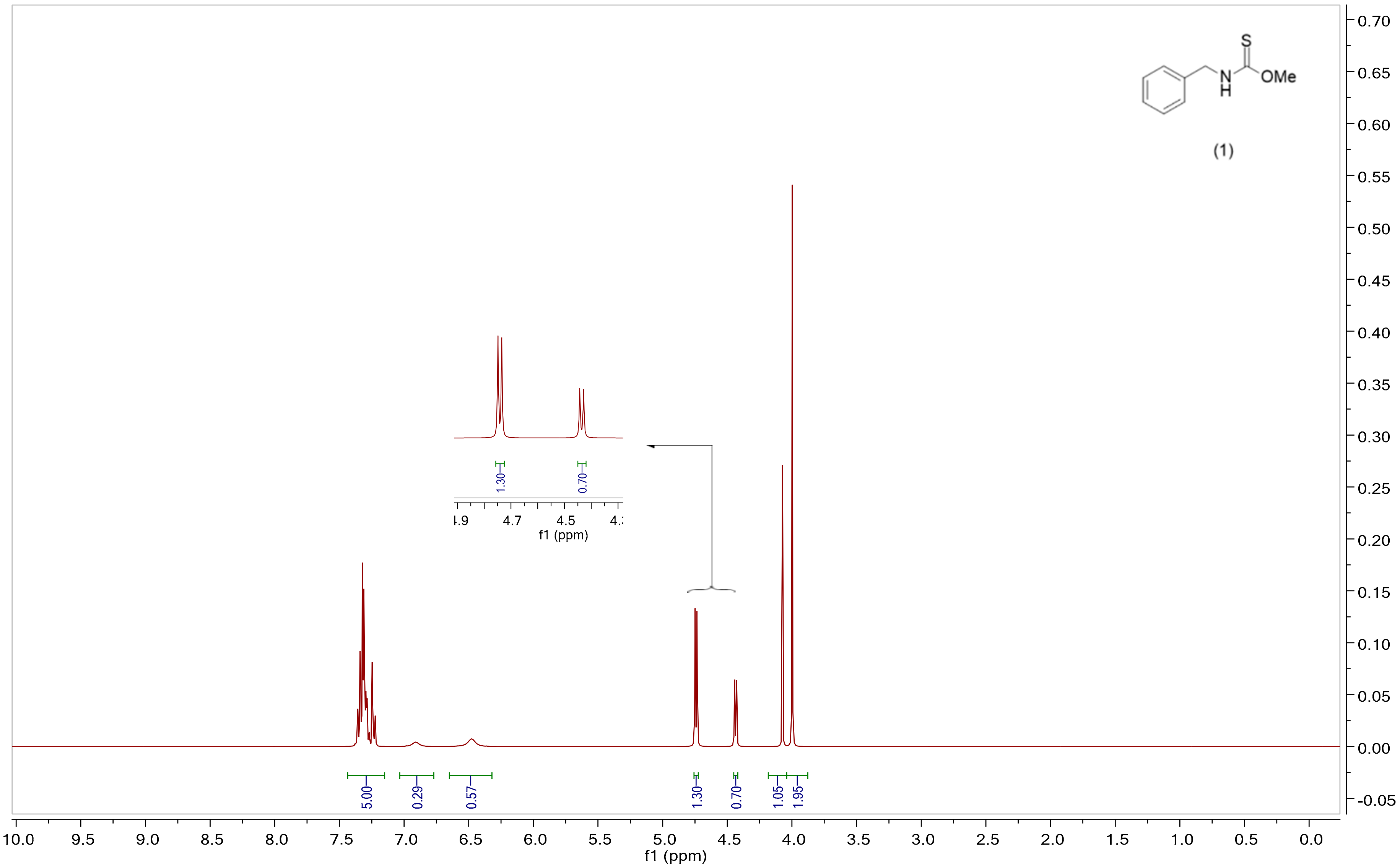

S1. ^{1}H NMR spectrum of analog **1**

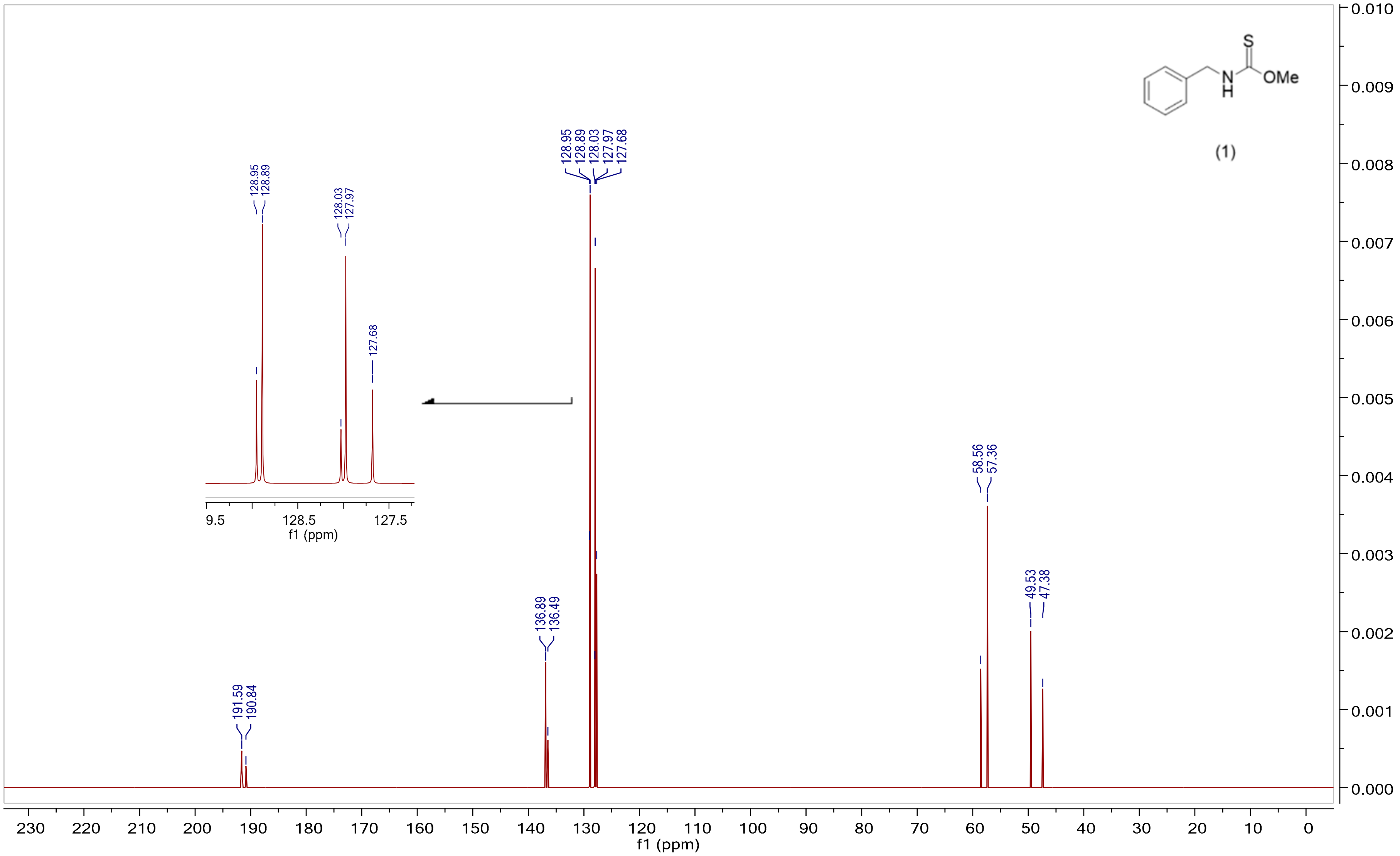

S2. ^{13}C NMR spectrum of analog **1**

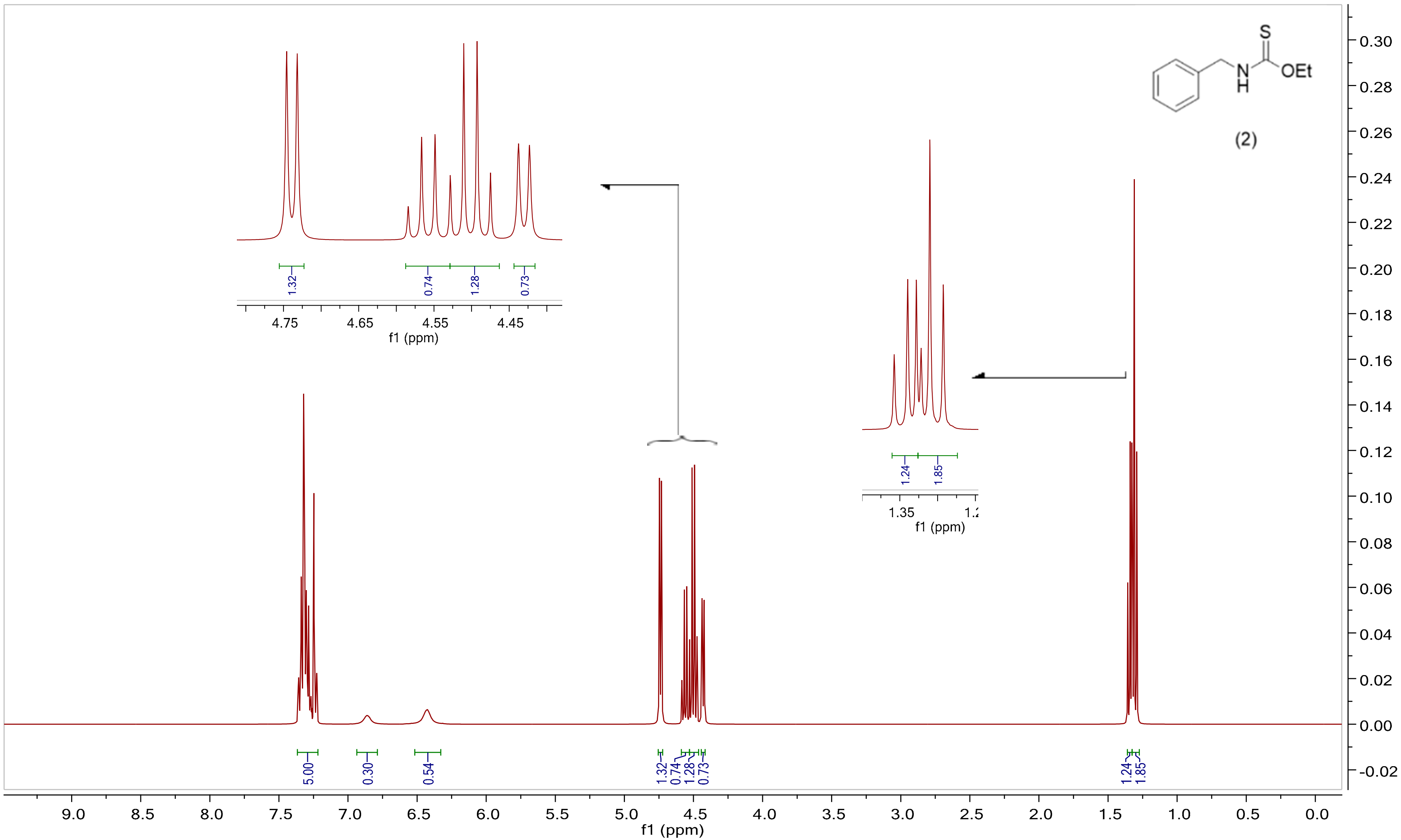

S3. ^{1}H NMR spectrum of analog **2**

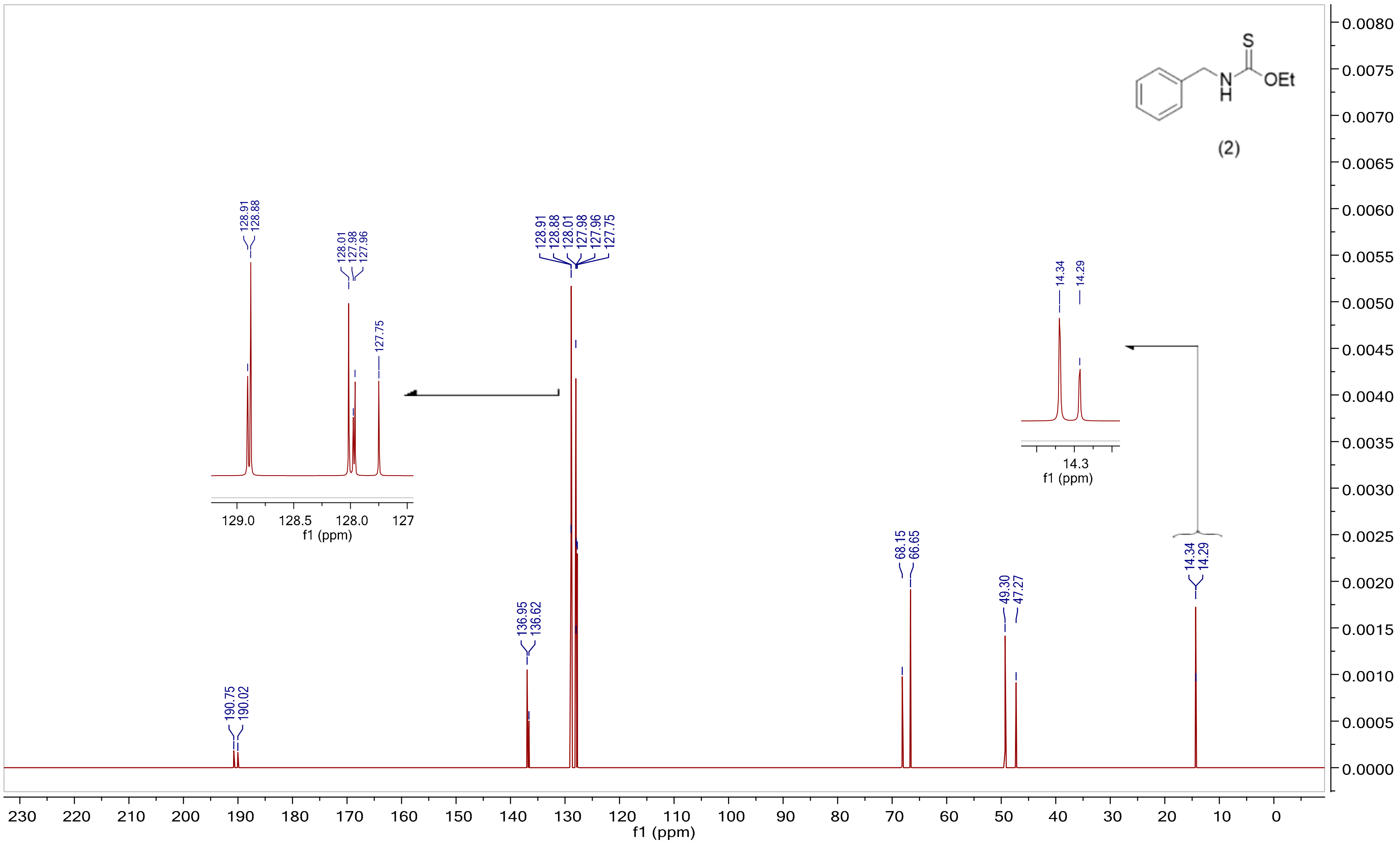

S4. ^{13}C NMR spectrum of analog **2**

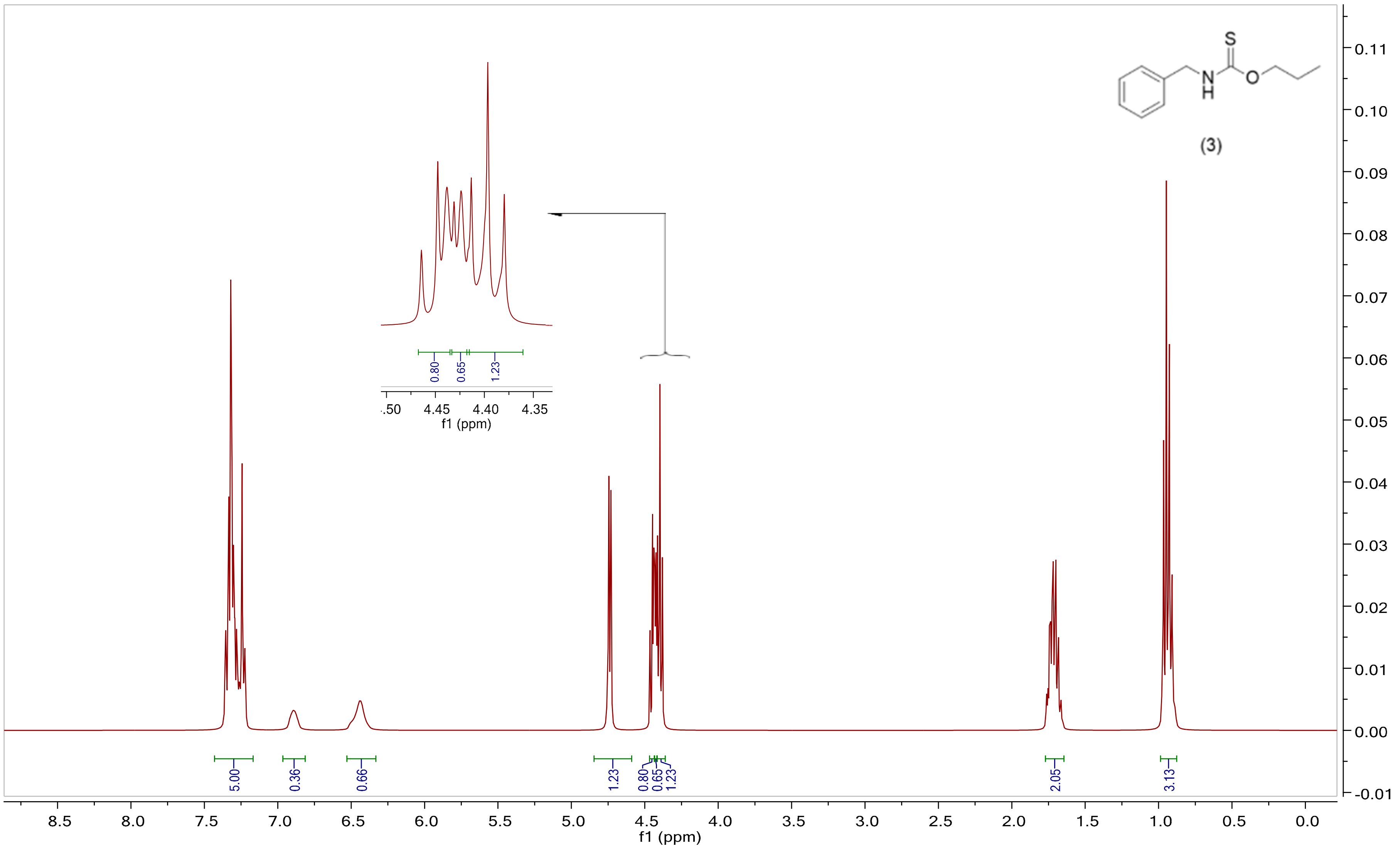

S5. ^{1}H NMR spectrum of analog **3**

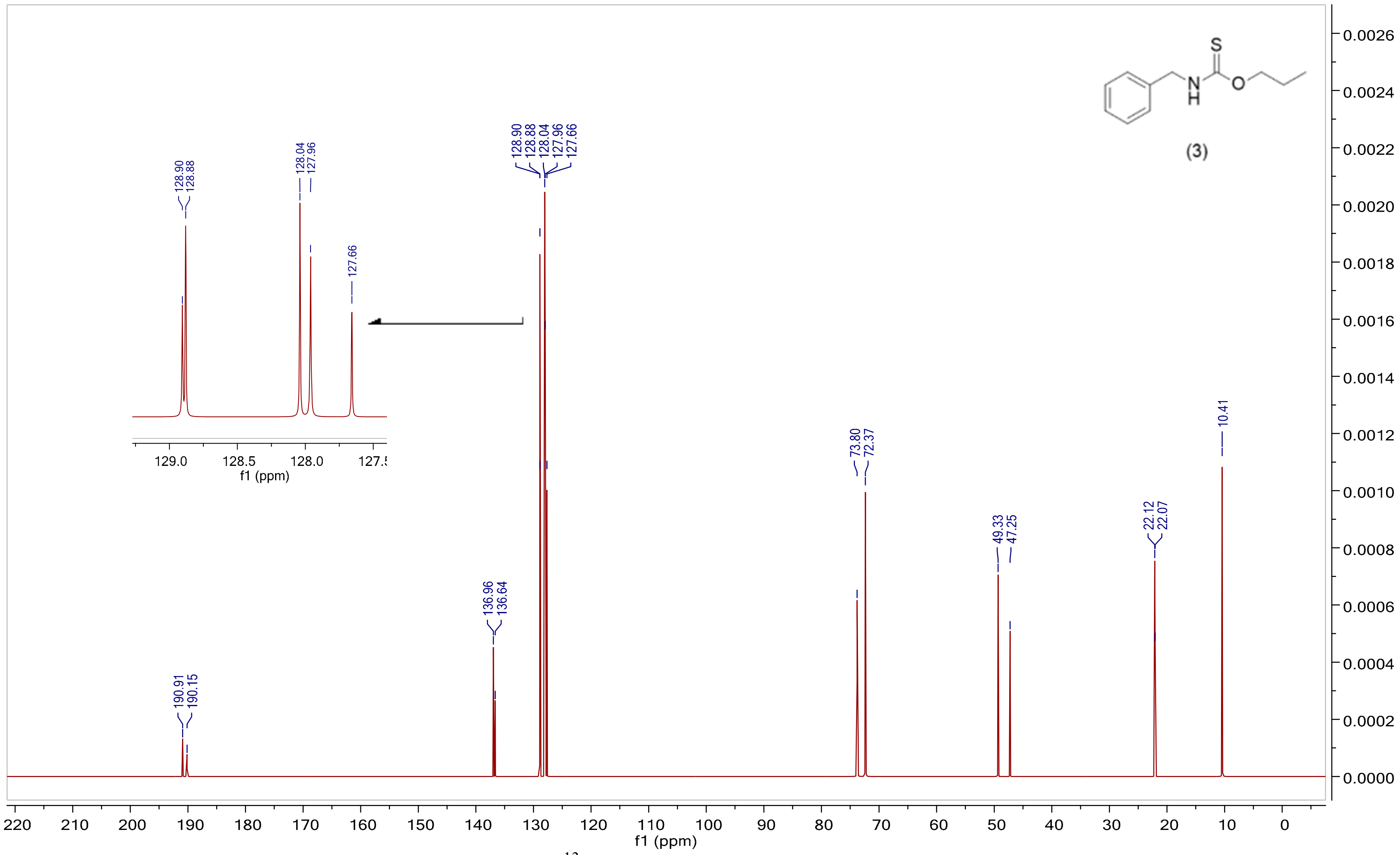

S6. ^{13}C NMR spectrum of analog **3**

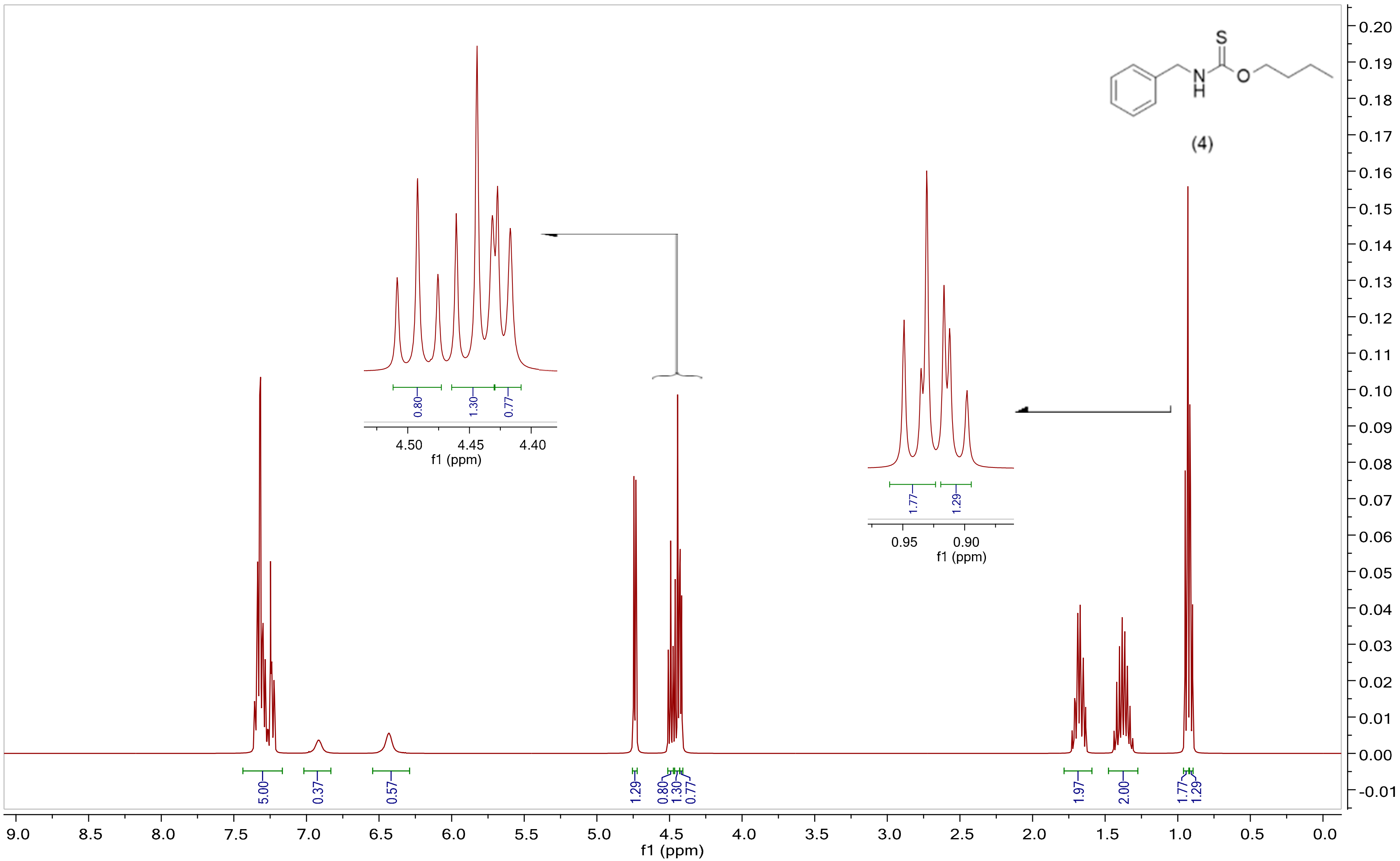

S7. ^{1}H NMR spectrum of analog **4**

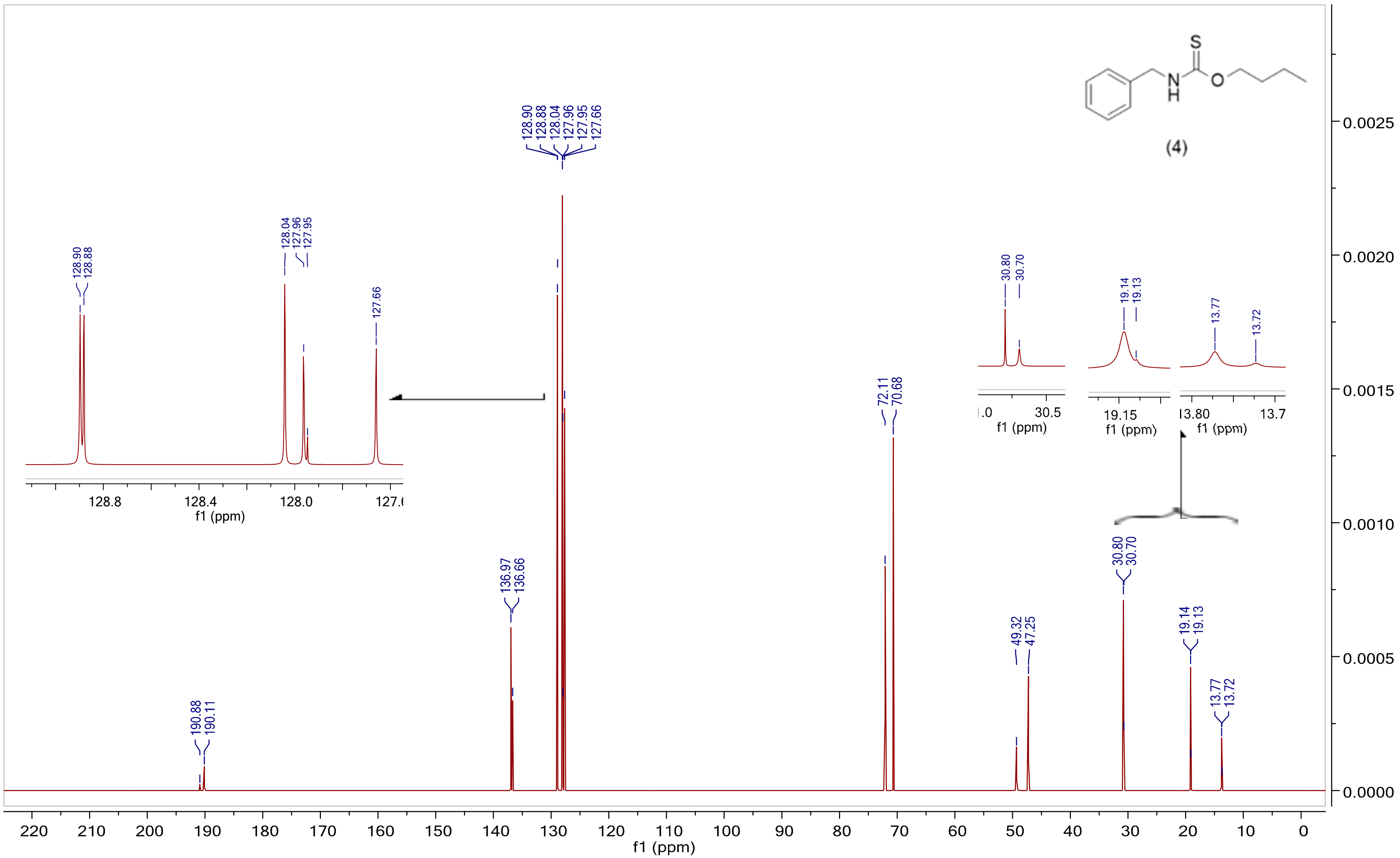

S8. ^{13}C NMR spectrum of analog **4**

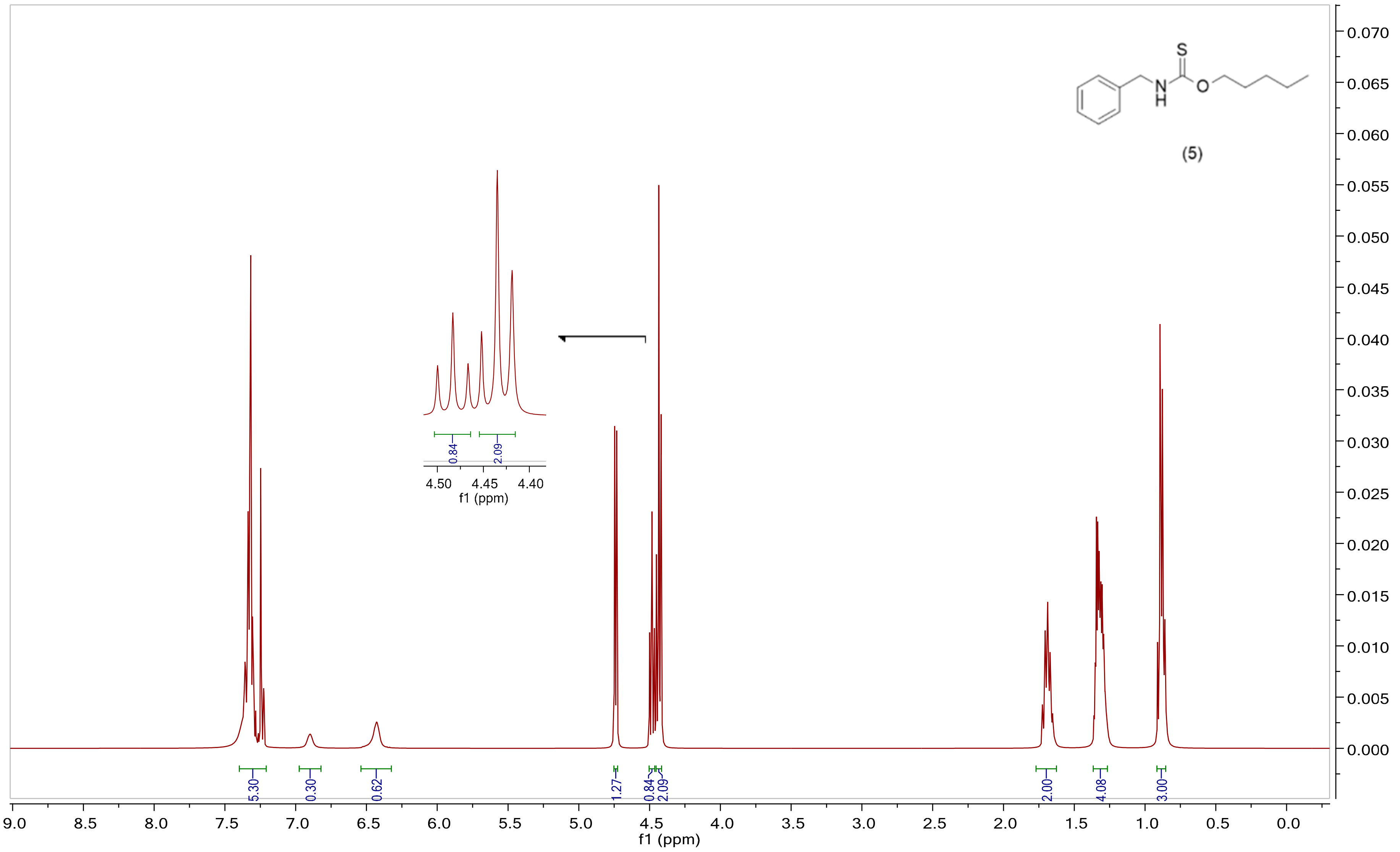

S9. ^{1}H NMR spectrum of analog **5**

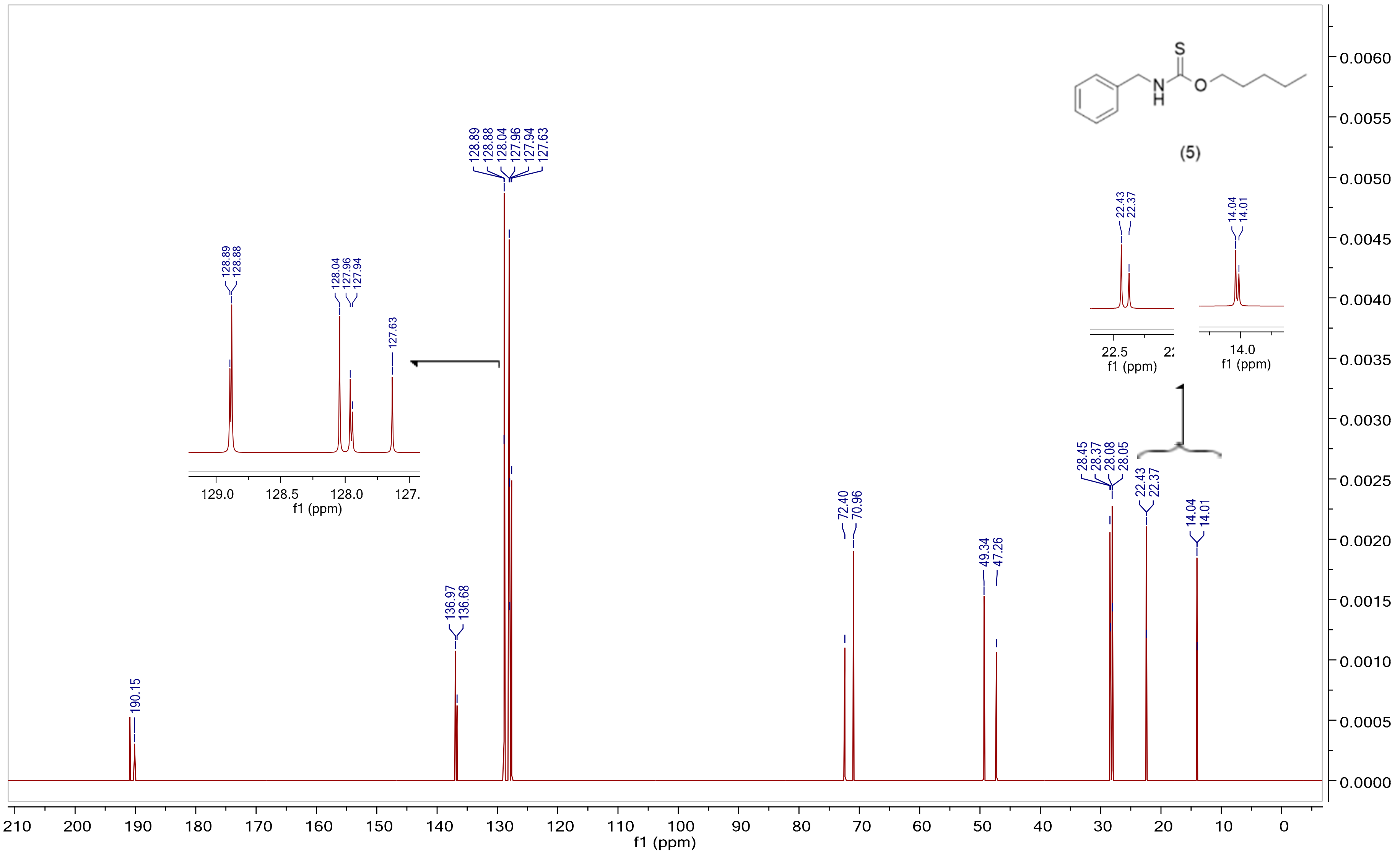

S10. ^{13}C NMR spectrum of analog **5**

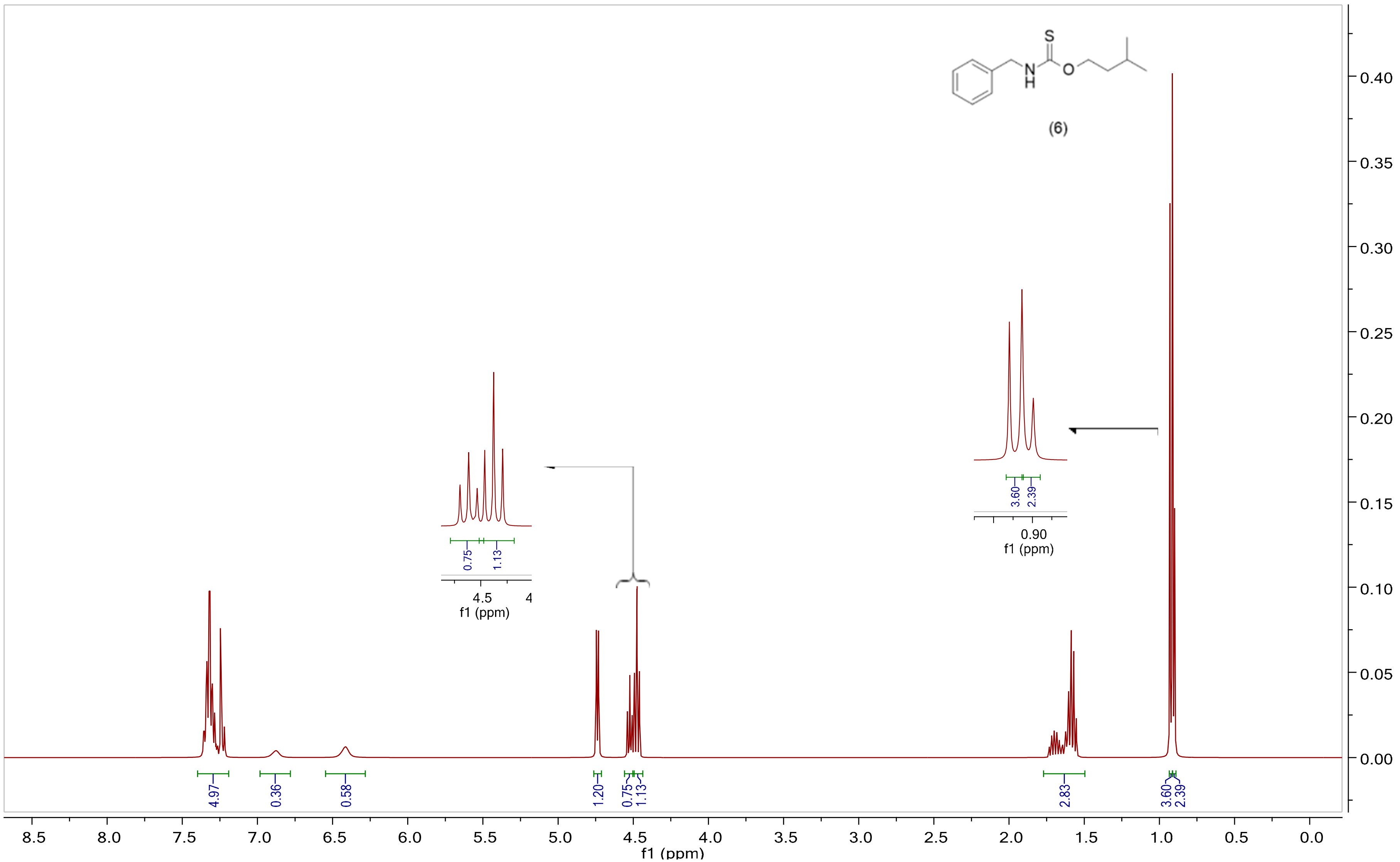

S11. ^{1}H NMR spectrum of analog **6**

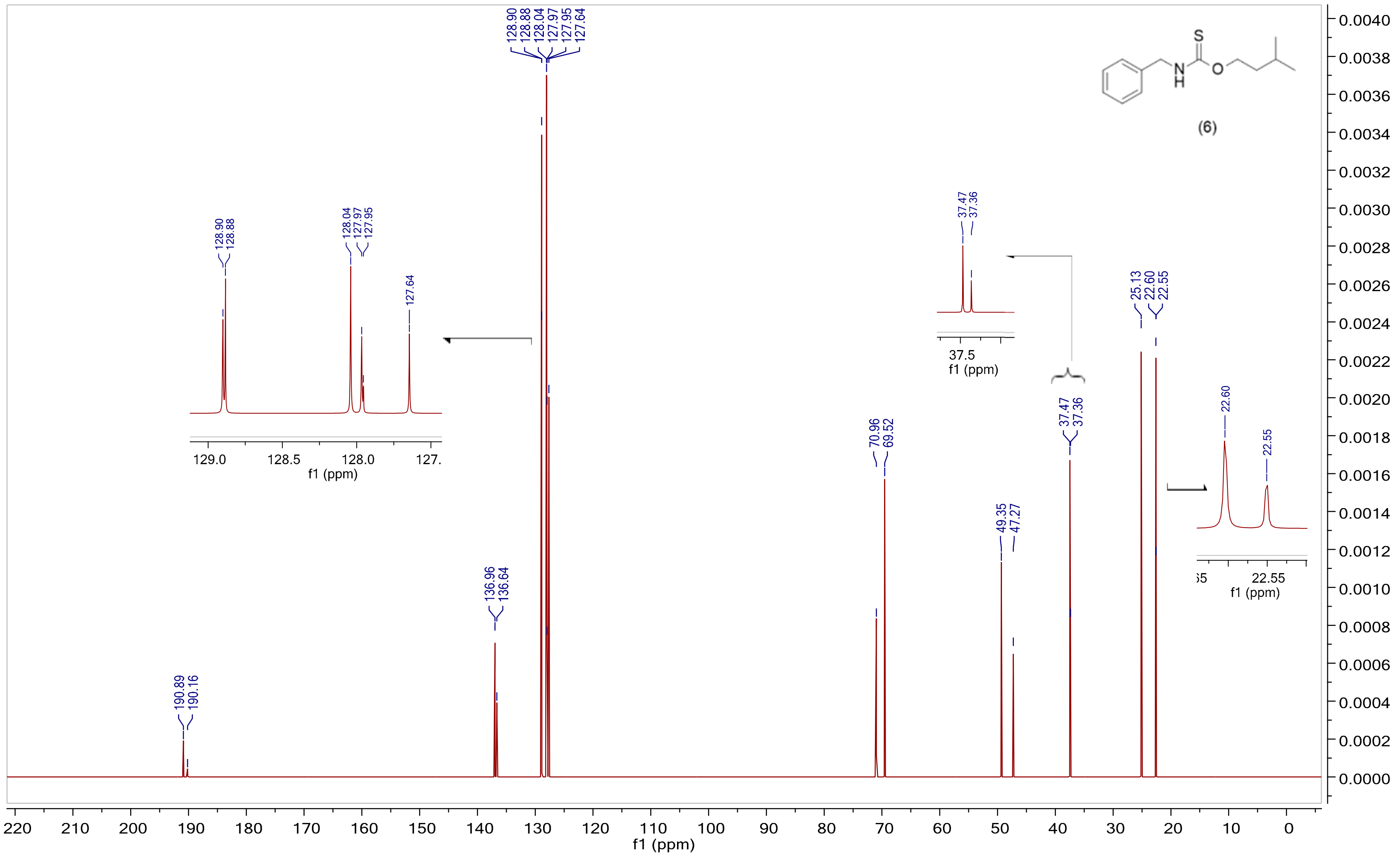

S12. ^{13}C NMR spectrum of analog **6**

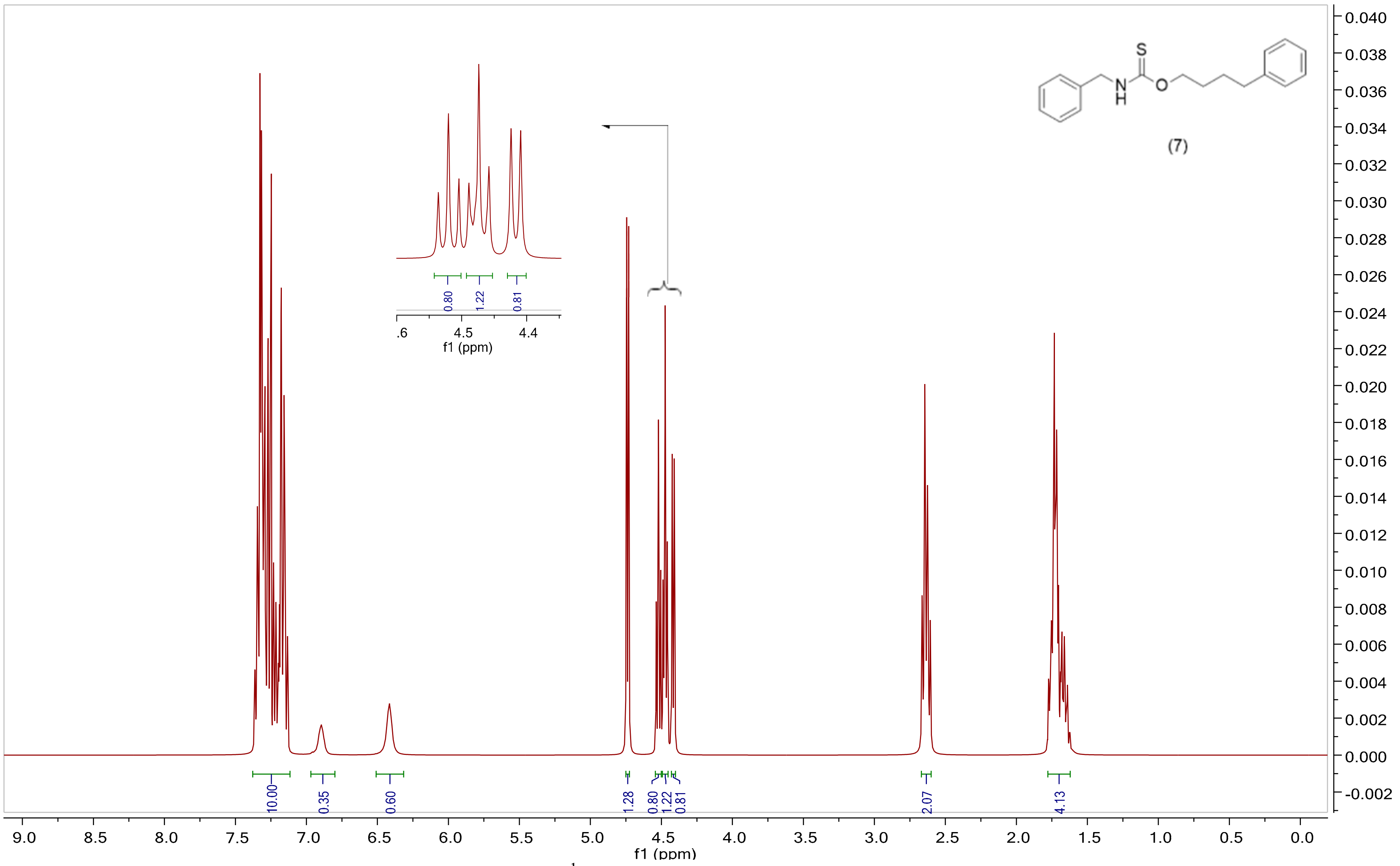

S13. ^{1}H NMR spectrum of analog **7**

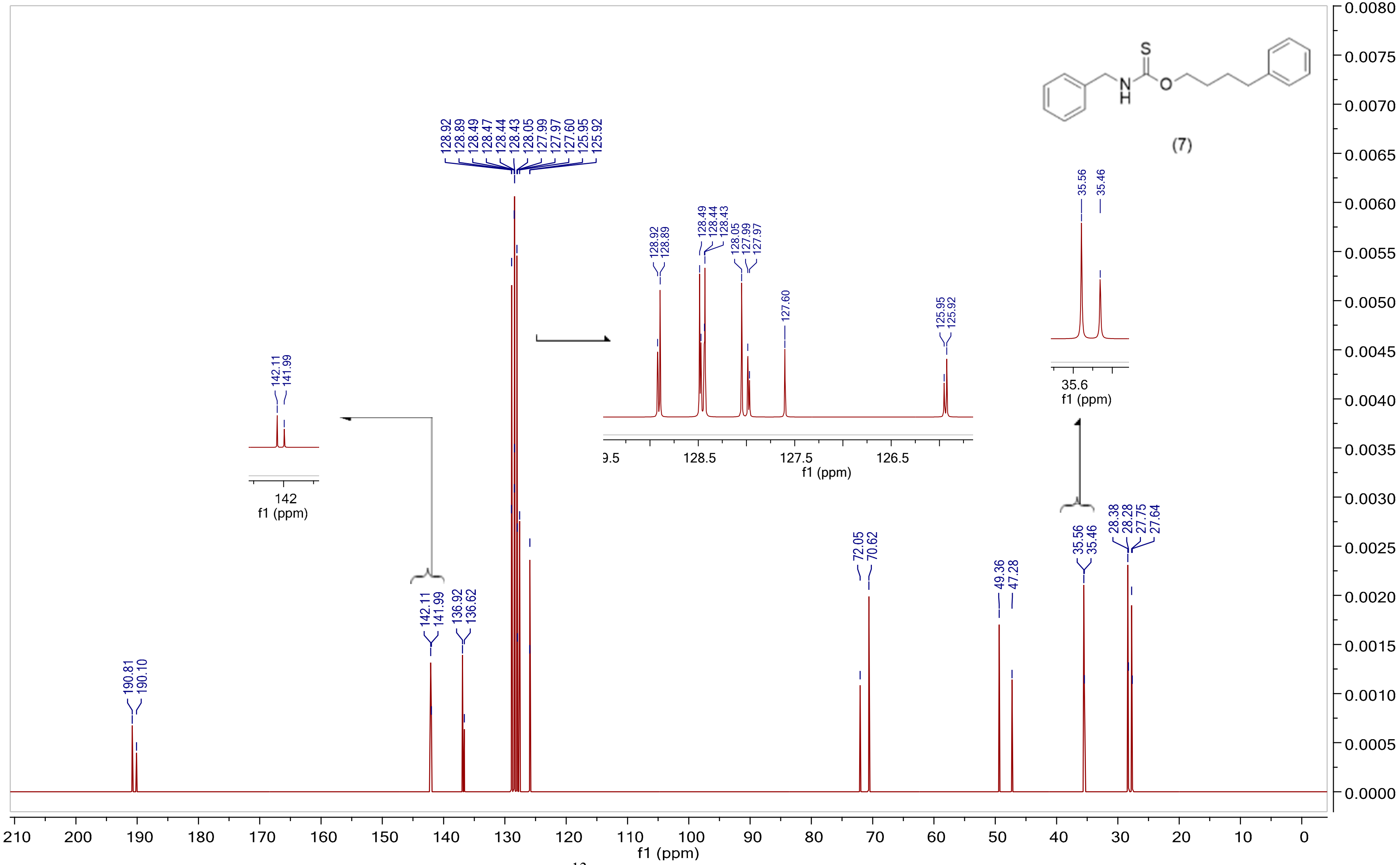

S14. ^{13}C NMR spectrum of analog **7**

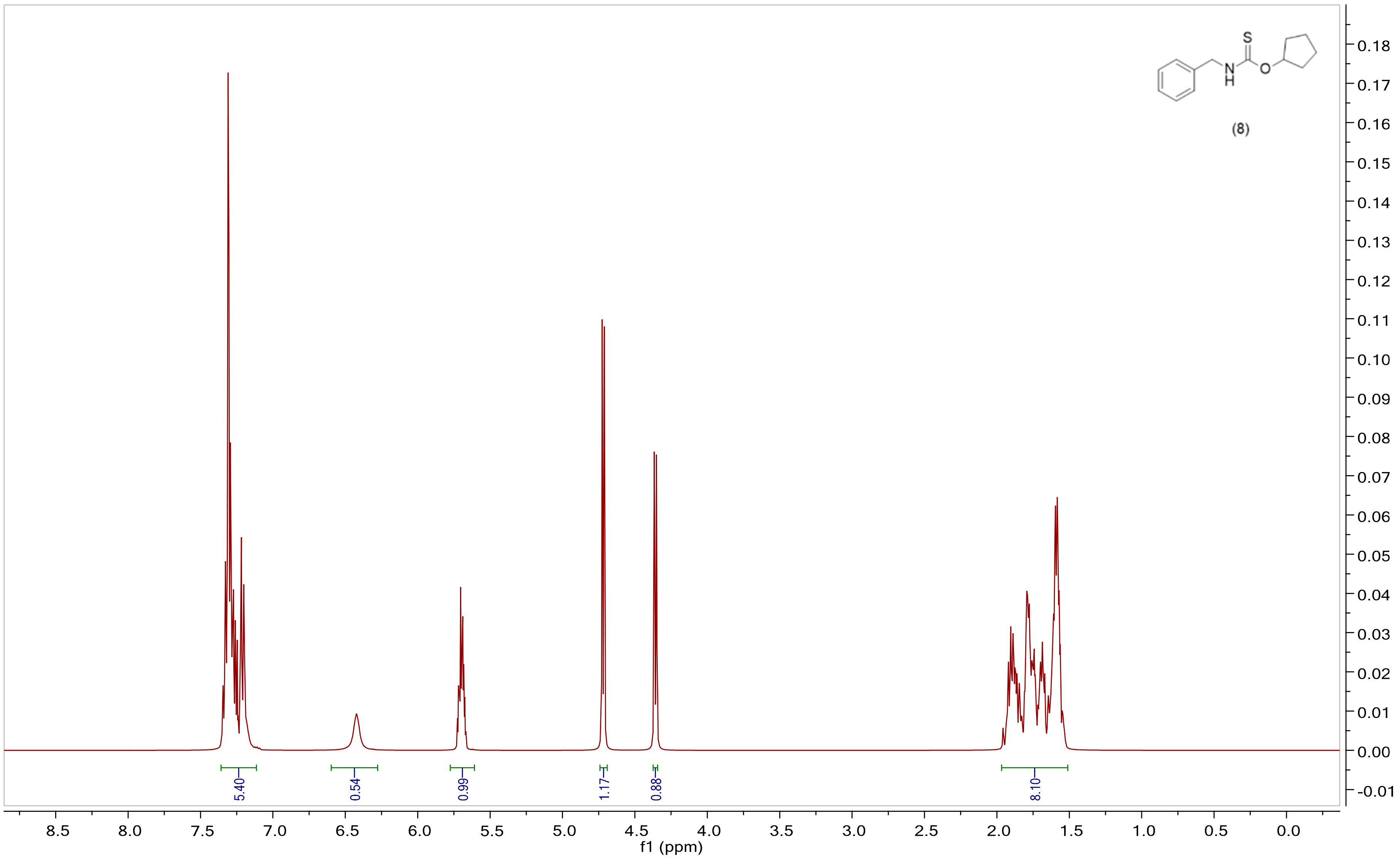

S15. ^{1}H NMR spectrum of analog **8**

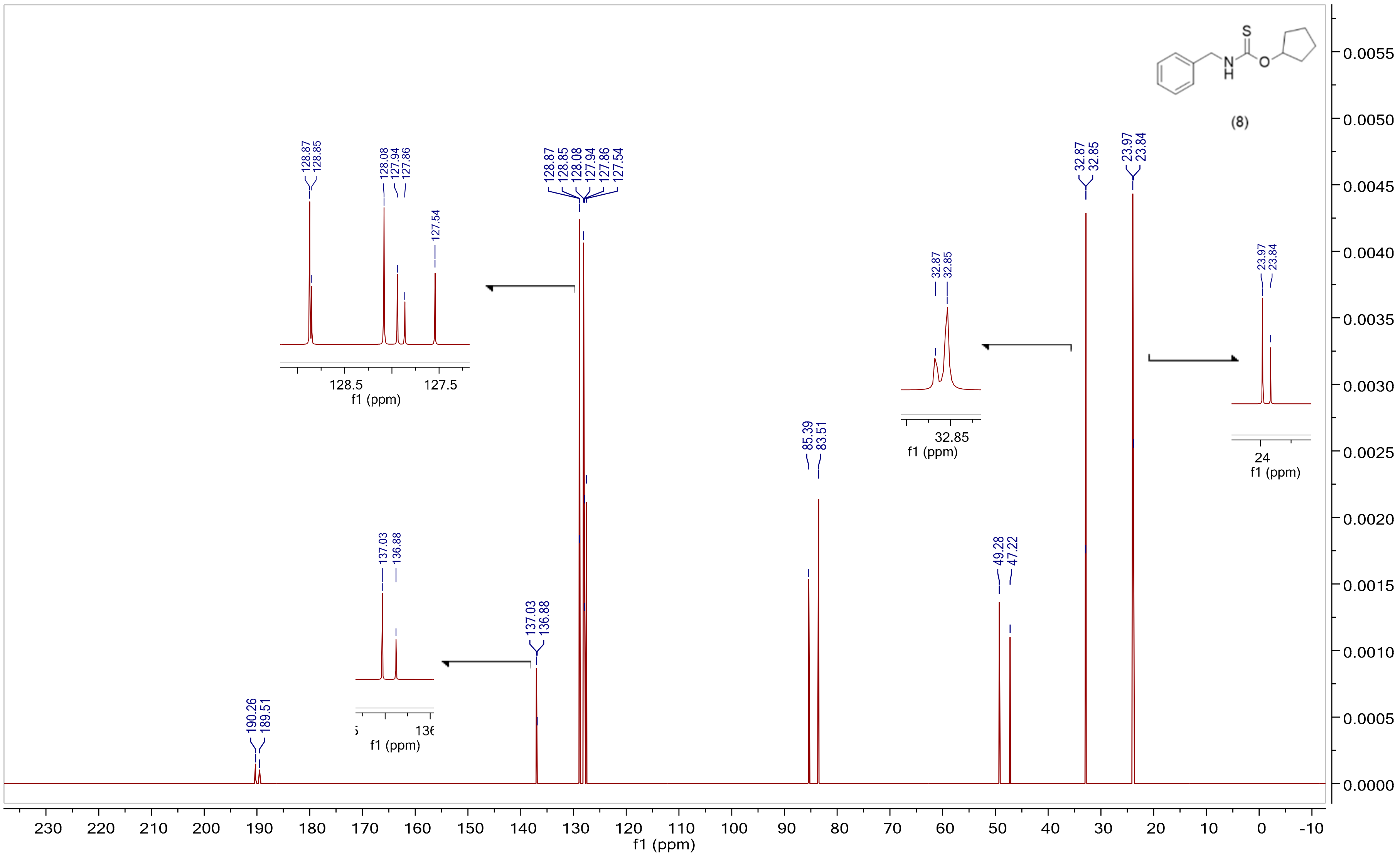

S16. ^{13}C NMR spectrum of analog **8**

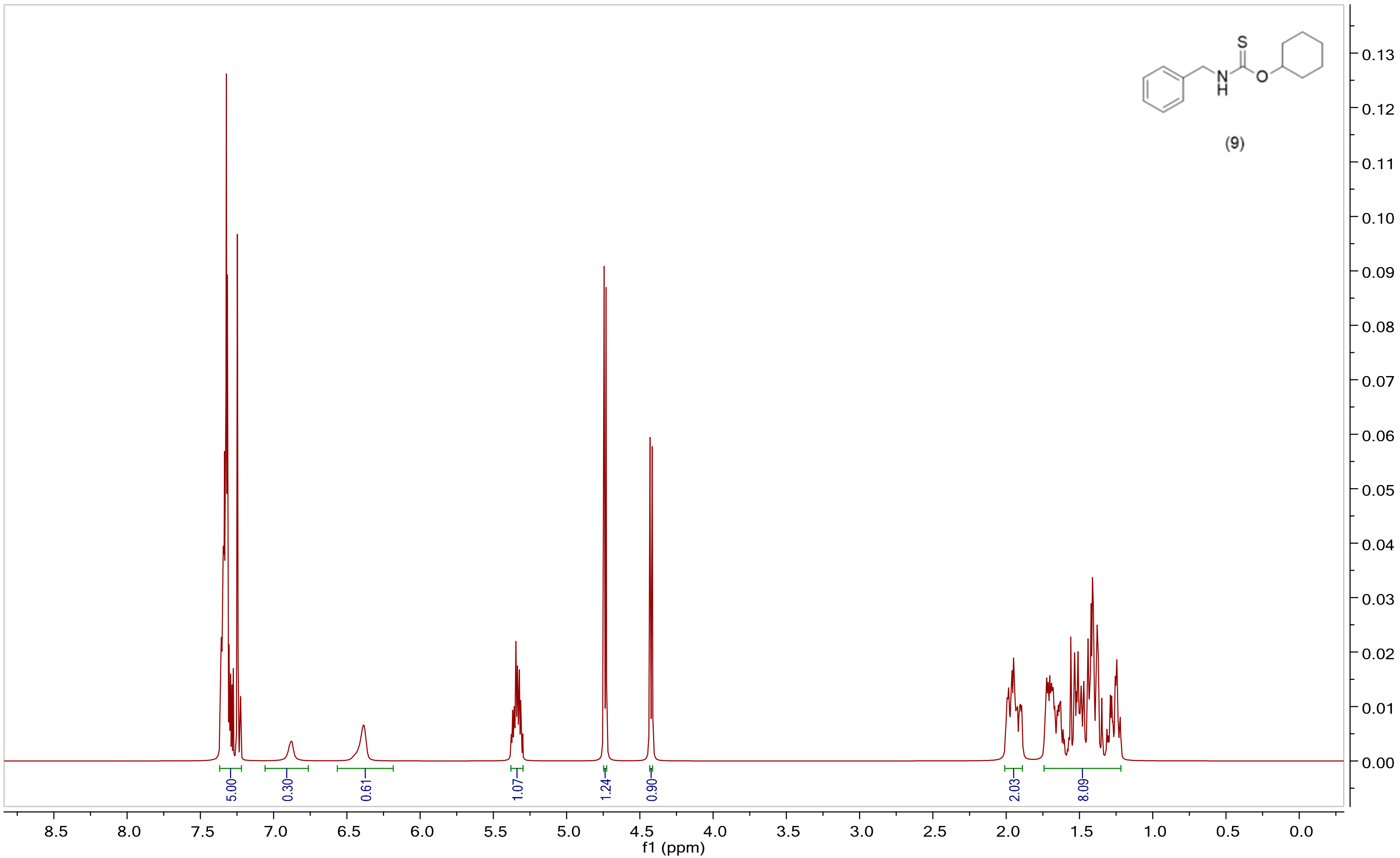

S17. ^{1}H NMR spectrum of analog **9**

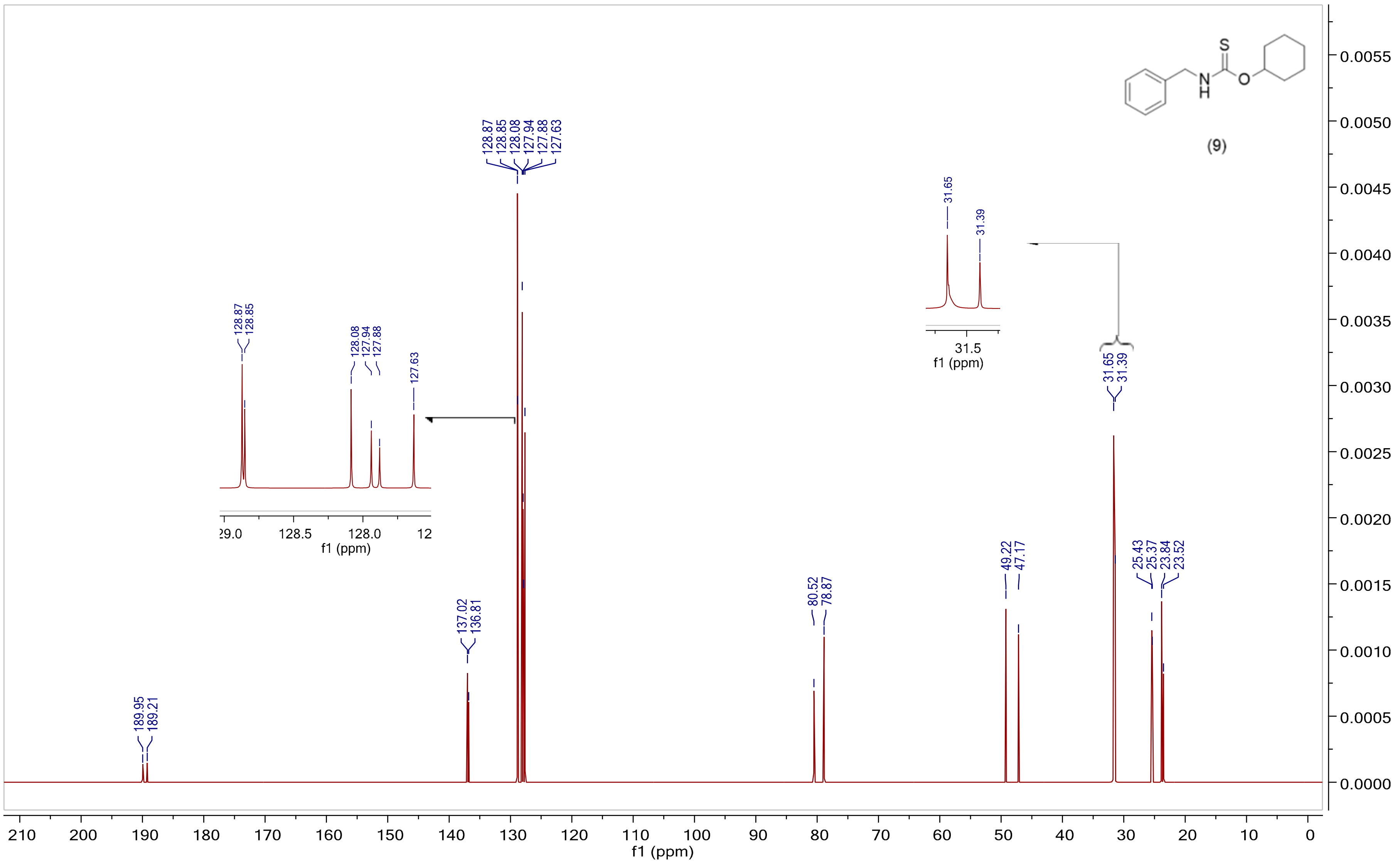

S18. ^{13}C NMR spectrum of analog **9**

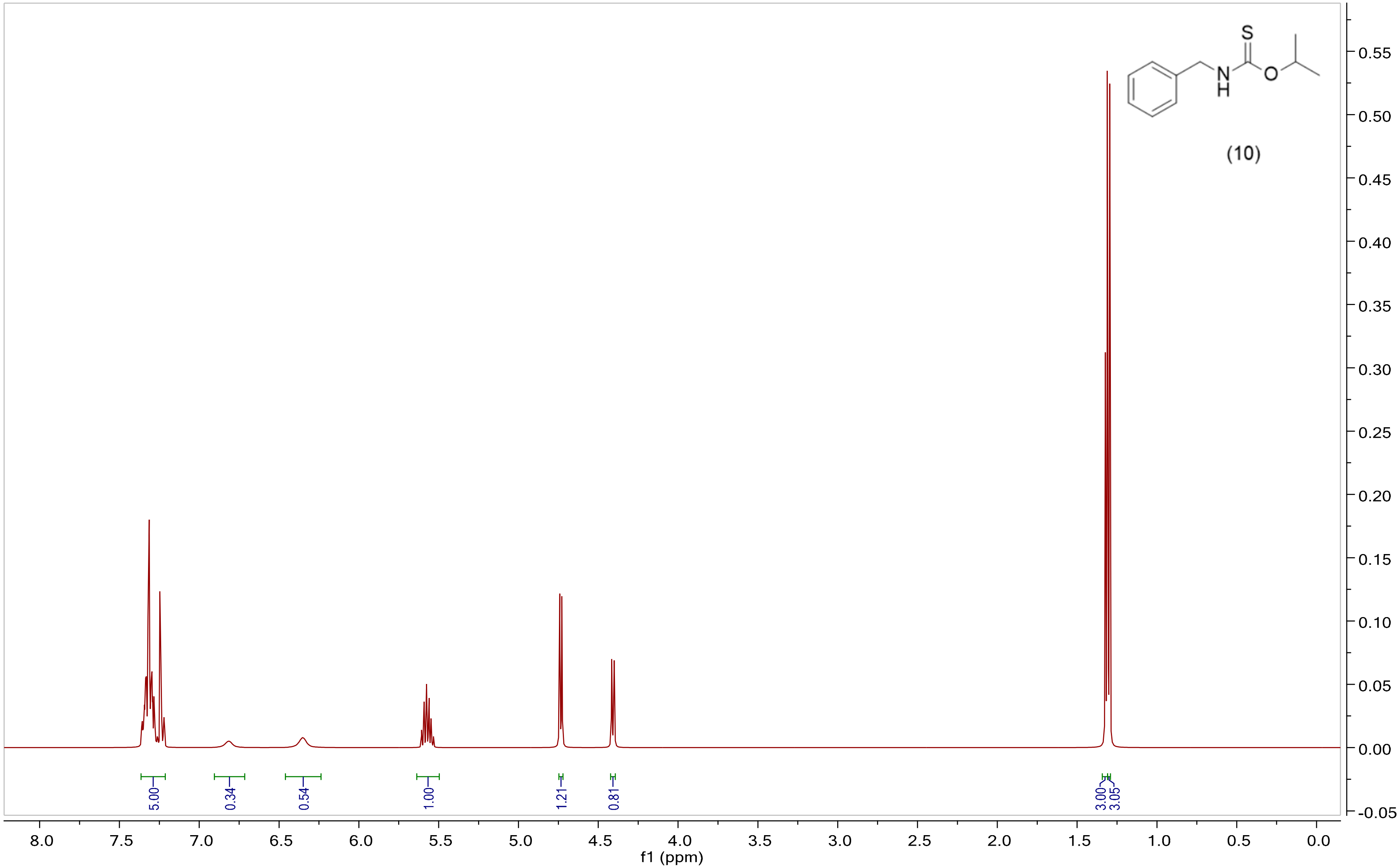

S19. ^{1}H NMR spectrum of analog **10**

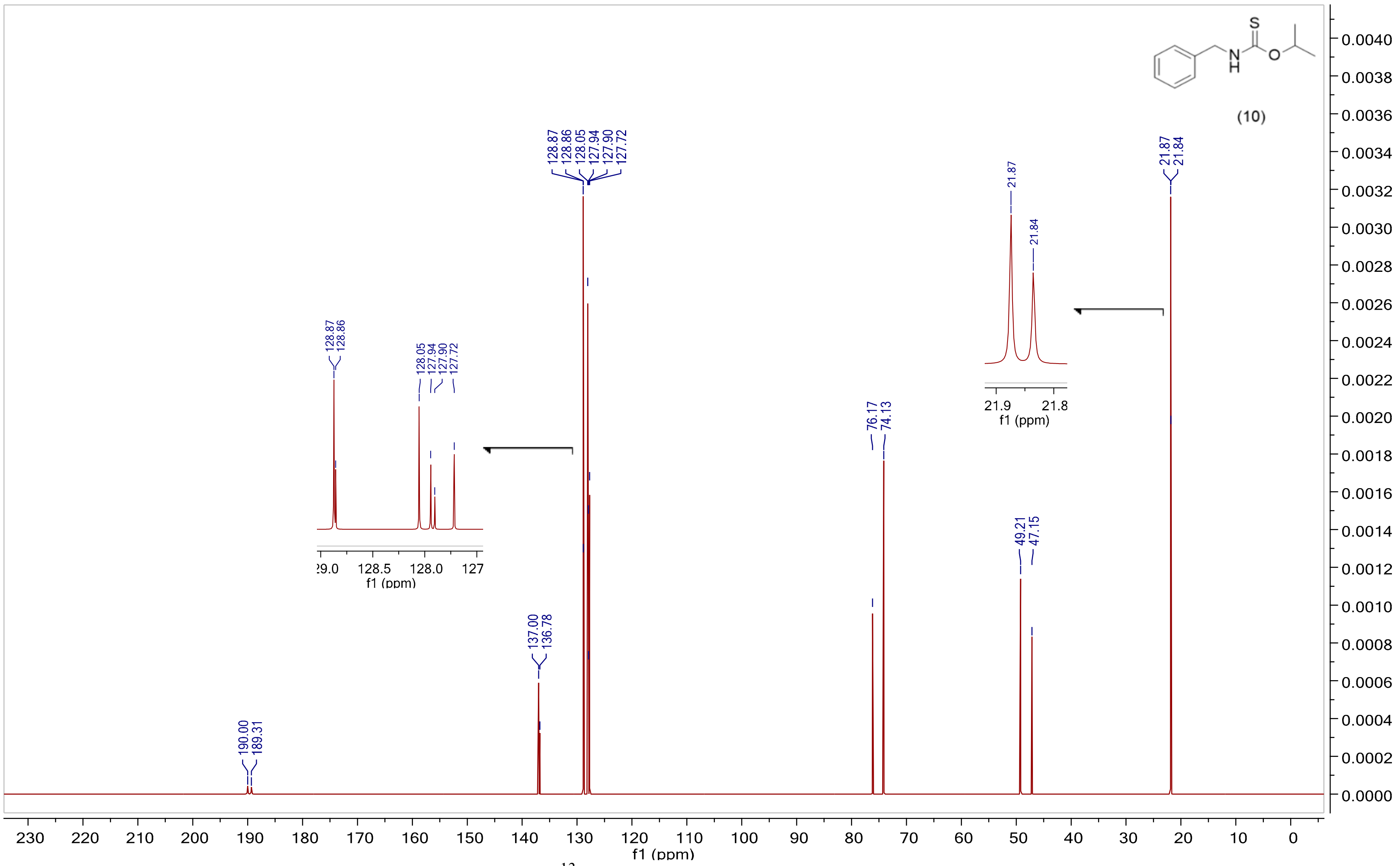

S20. ^{13}C NMR spectrum of analog **10**

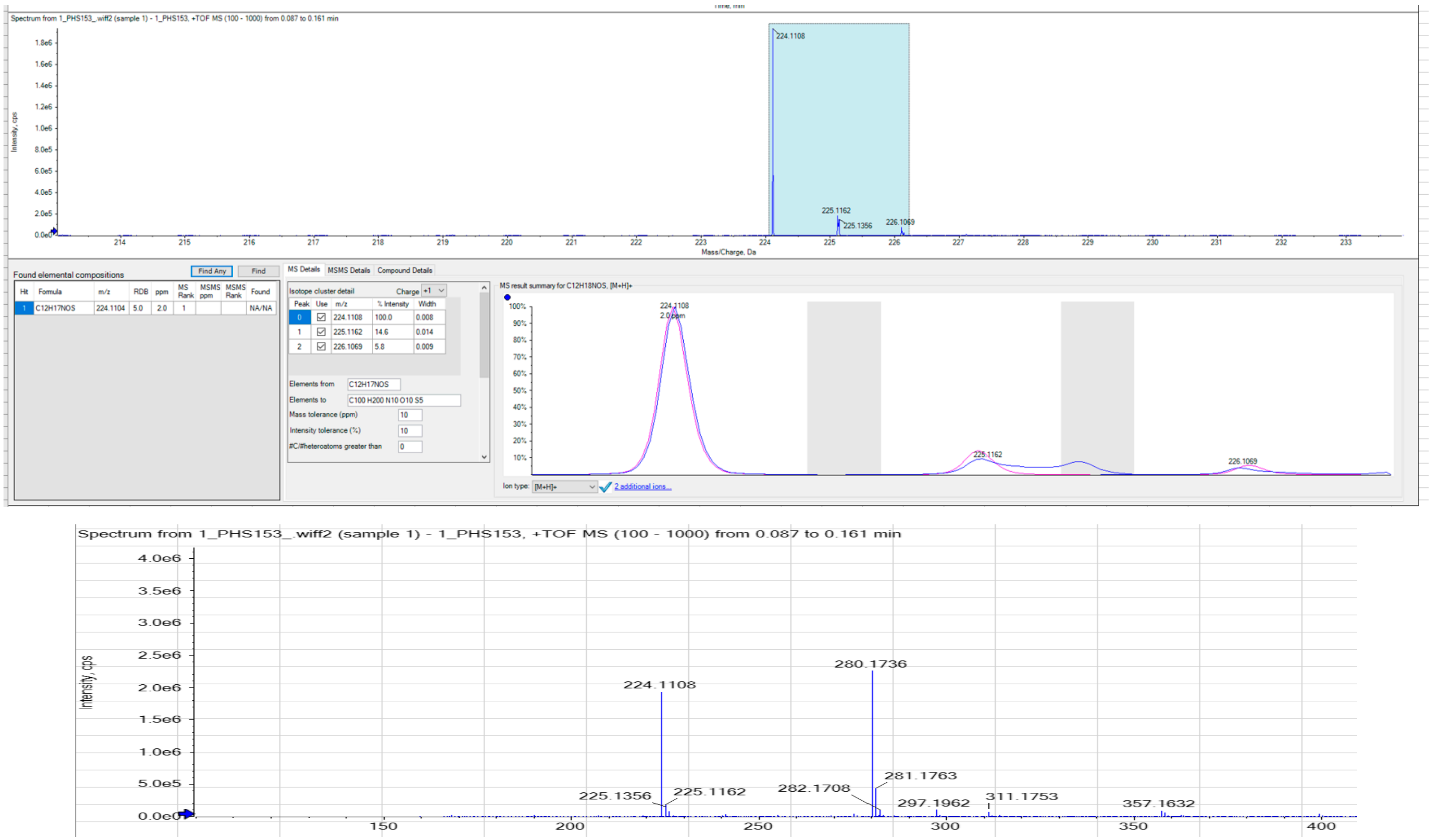

S21. HRMS spectrum of analog **4**

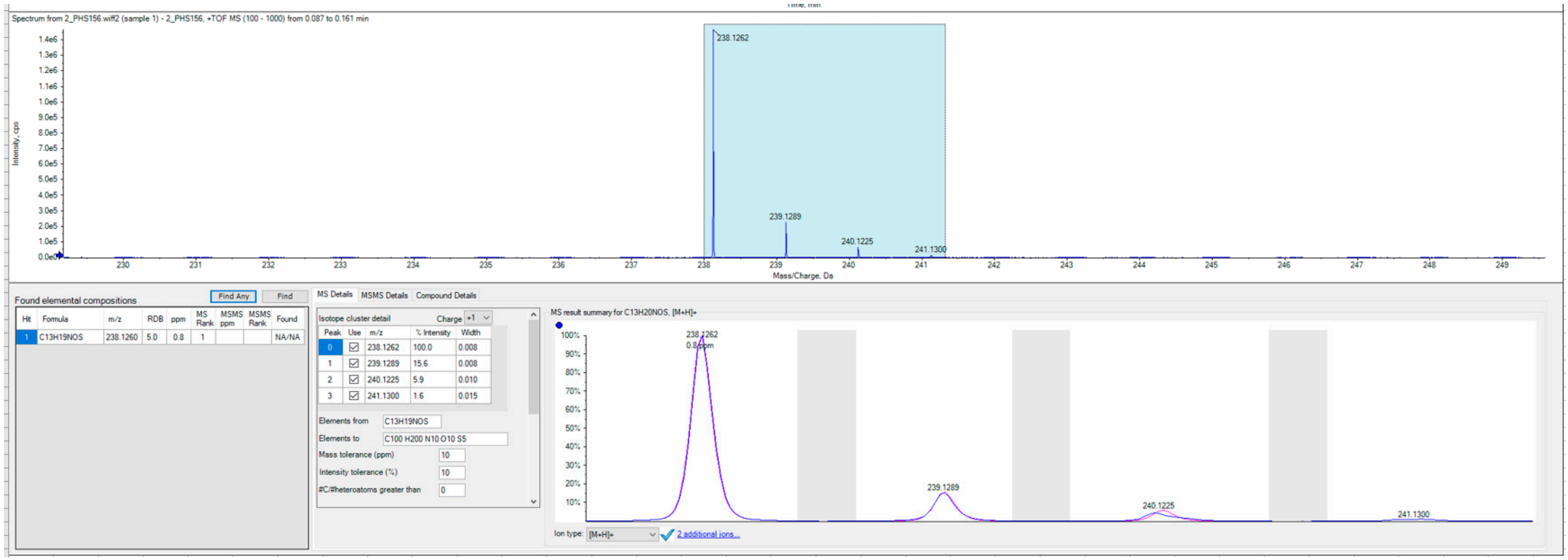

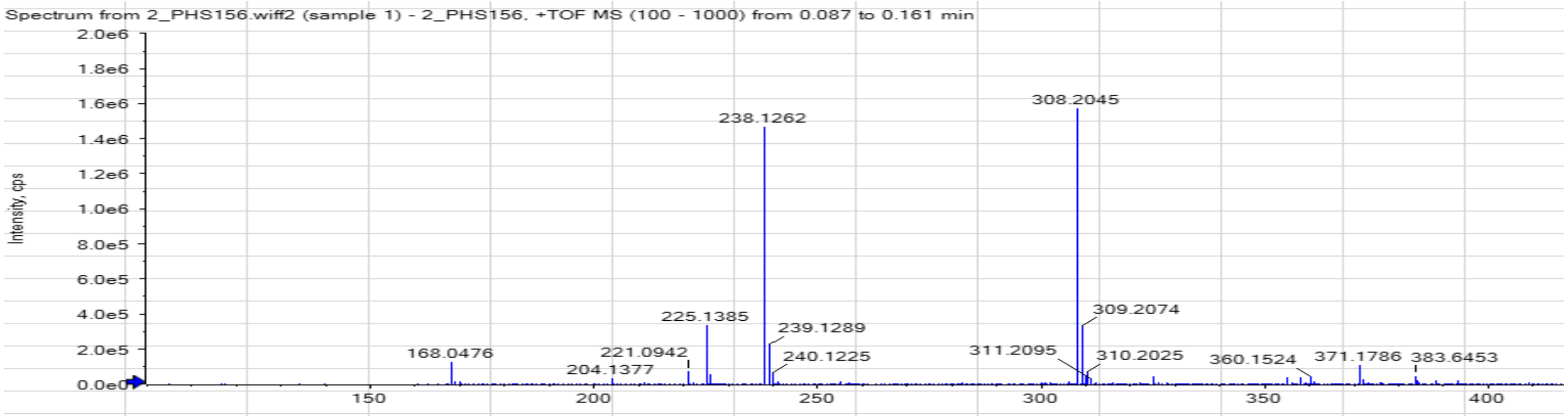

S22. HRMS spectrum of analog **5**

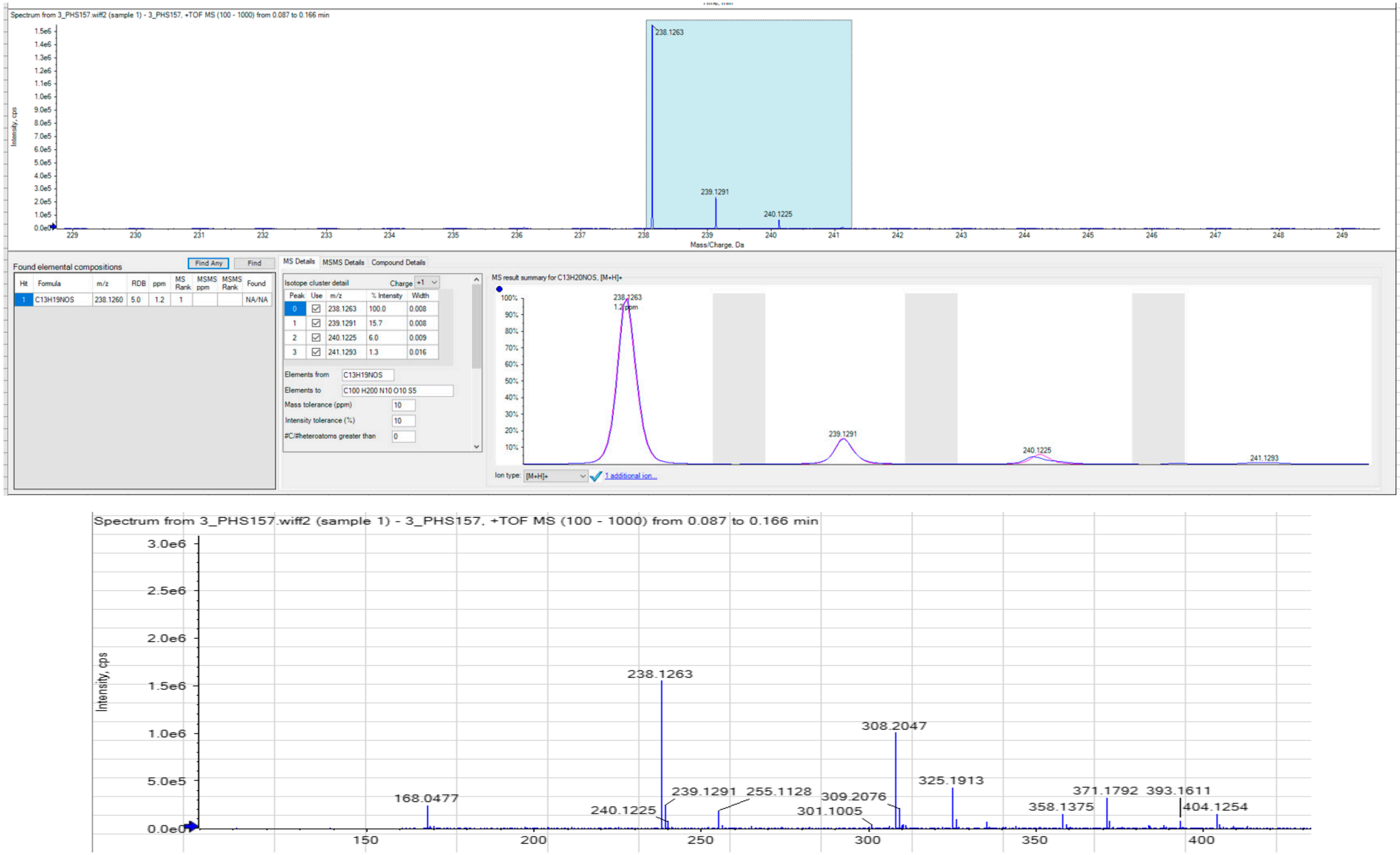

S23. HRMS spectrum of analog **6**

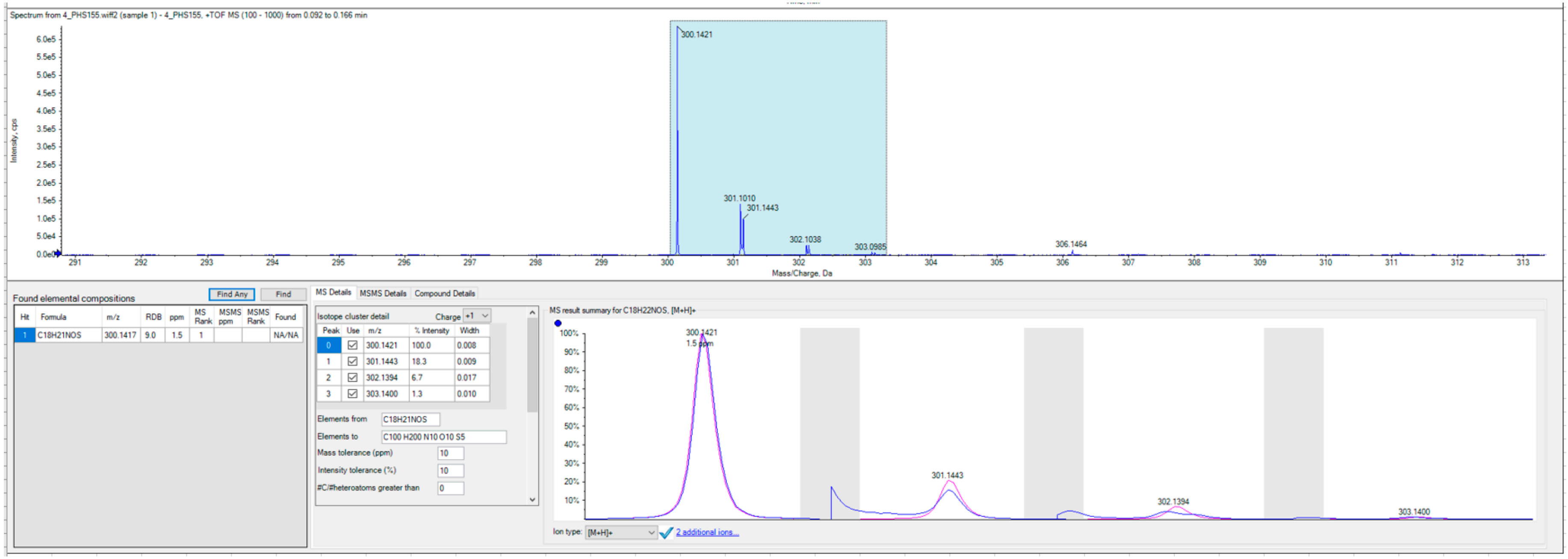

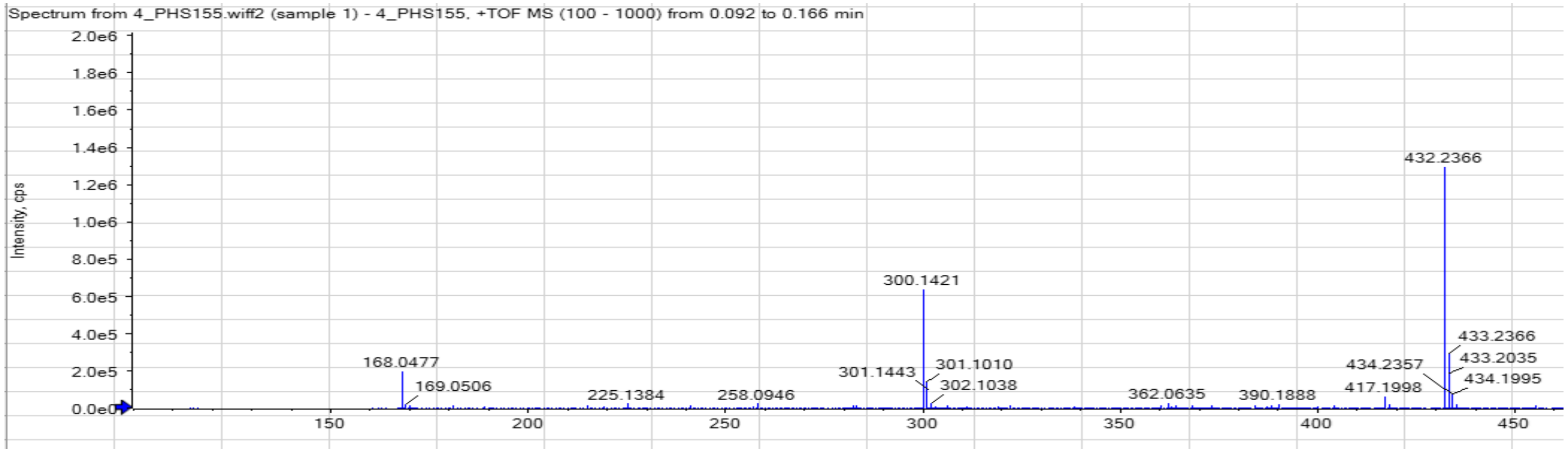

S24. HRMS spectrum of analog **7**

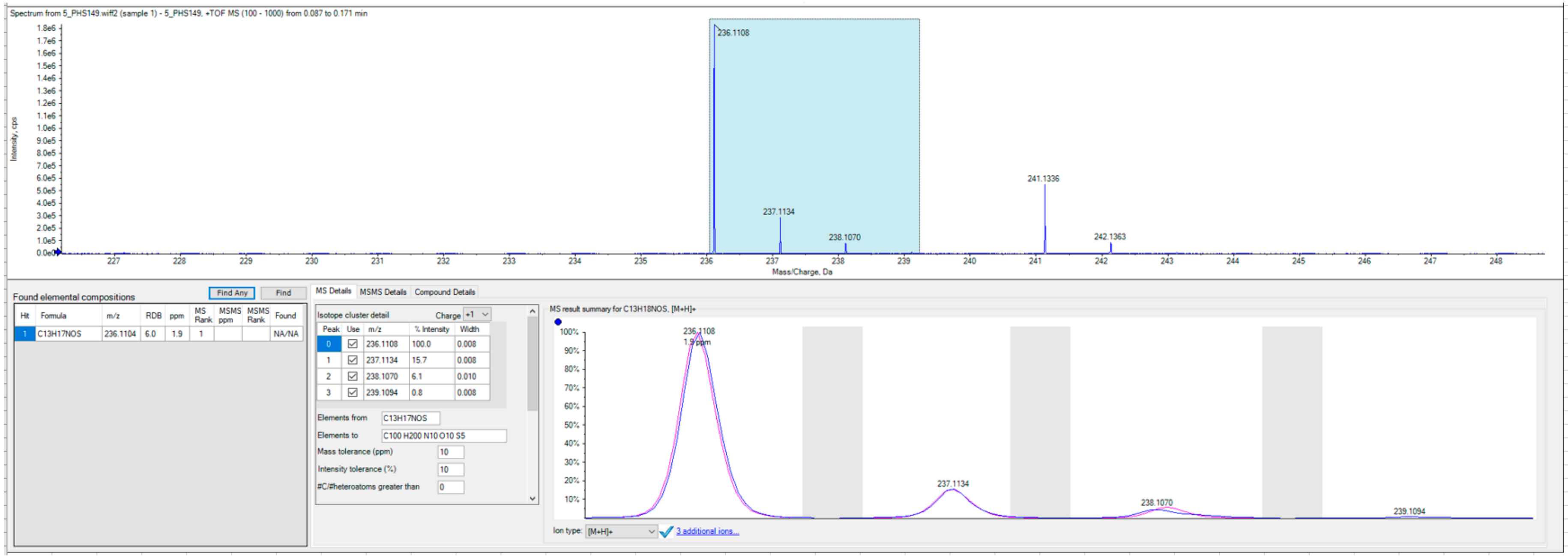

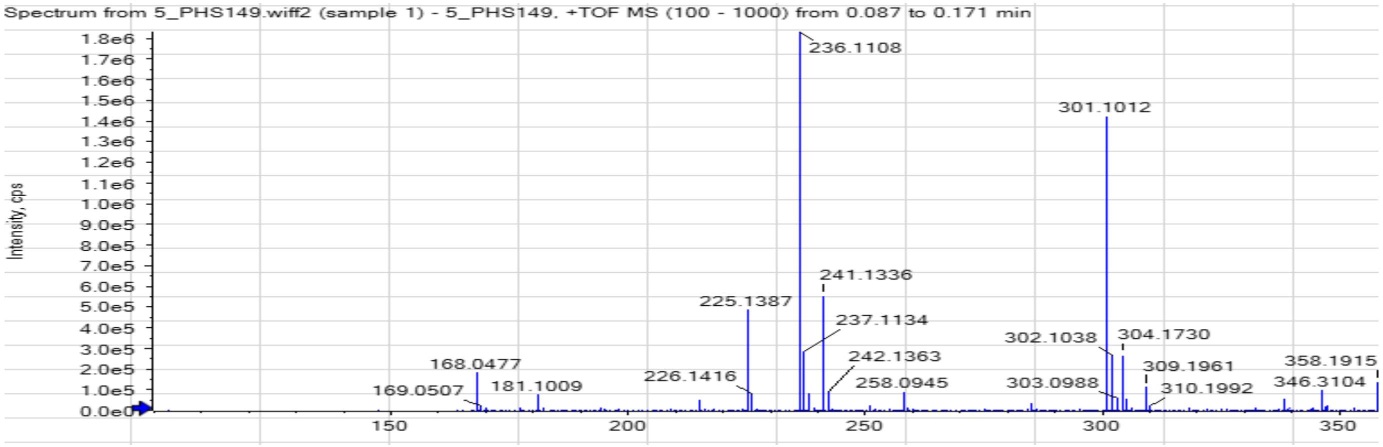

S25. HRMS spectrum of analog **8**

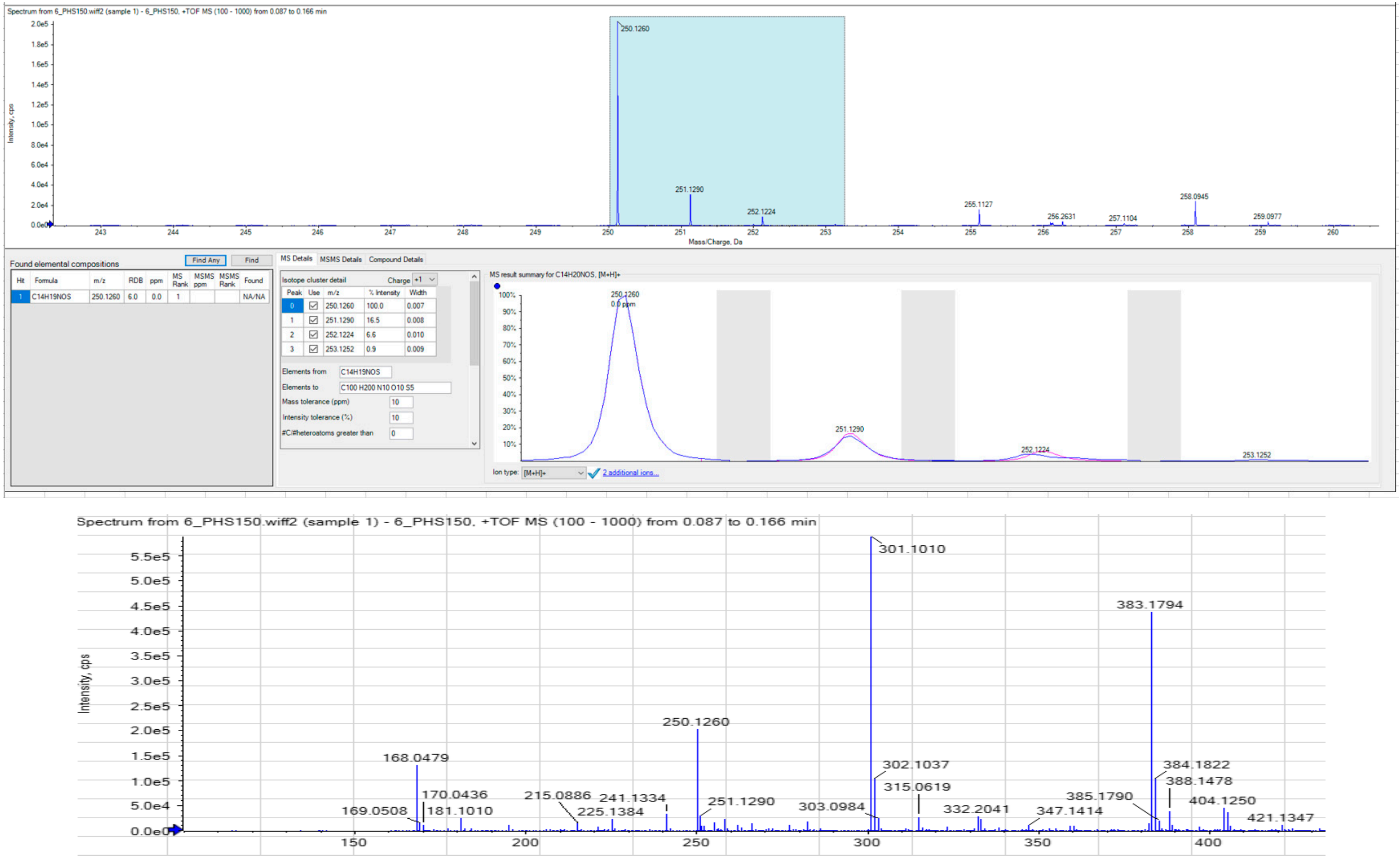

S26. HRMS spectrum of analog **9**

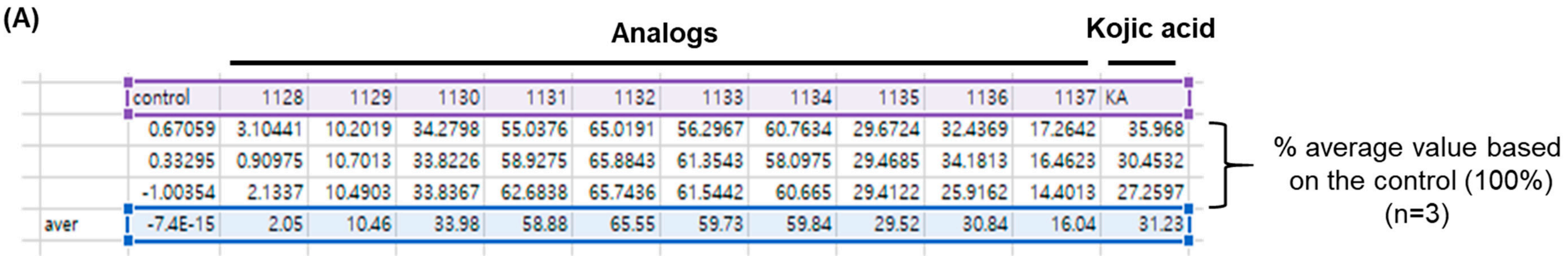

	control	1128	1129	1130	1131	1132	1133	1134	1135	1136	1137	KA
	0.67059	3.10441	10.2019	34.2798	55.0376	65.0191	56.2967	60.7634	29.6724	32.4369	17.2642	35.968
	0.33295	0.90975	10.7013	33.8226	58.9275	65.8843	61.3543	58.0975	29.4685	34.1813	16.4623	30.4532
	-1.00354	2.1337	10.4903	33.8367	62.6838	65.7436	61.5442	60.665	29.4122	25.9162	14.4013	27.2597
aver	-7.4E-15	2.05	10.46	33.98	58.88	65.55	59.73	59.84	29.52	30.84	16.04	31.23

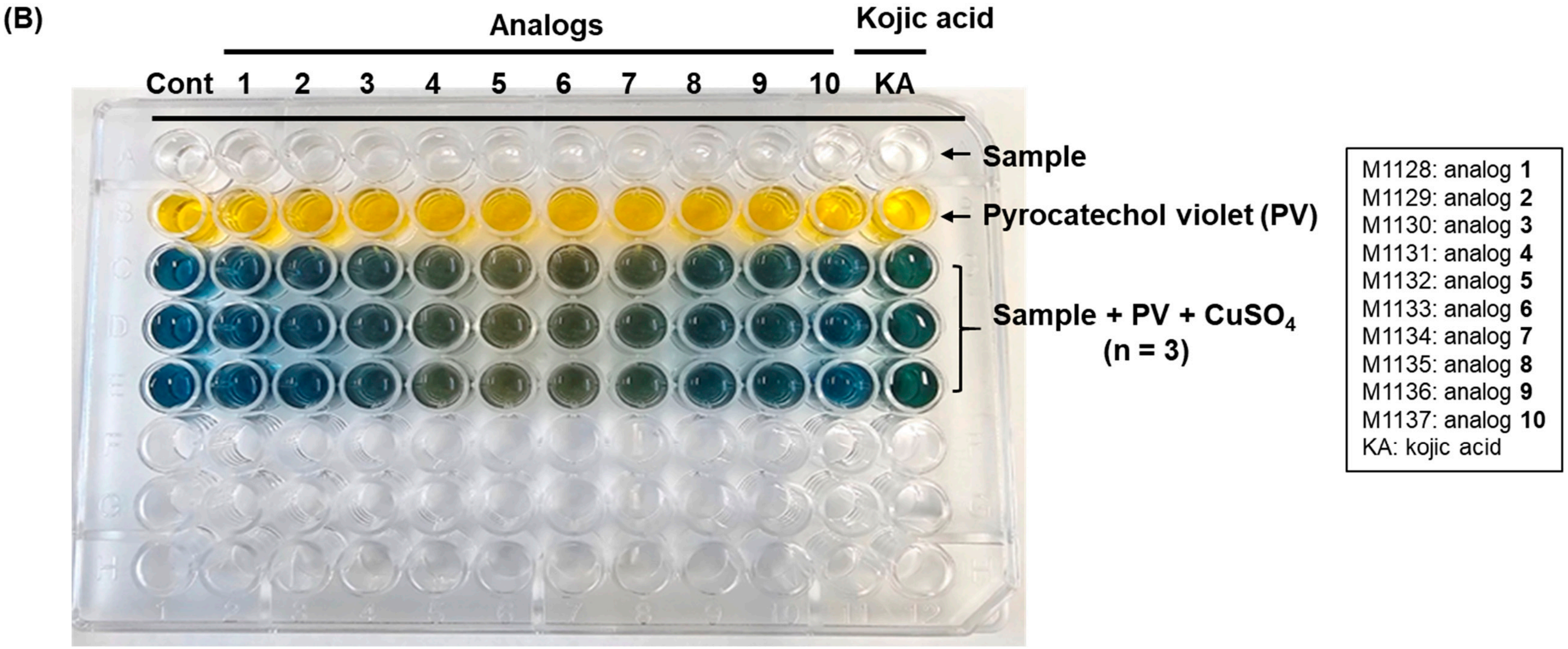

S27. Original data (A) and photo (B) for Cu^{2+} chelation activity of NBTC analogs **1**-**10**.

Substrate: L-Tyrosine

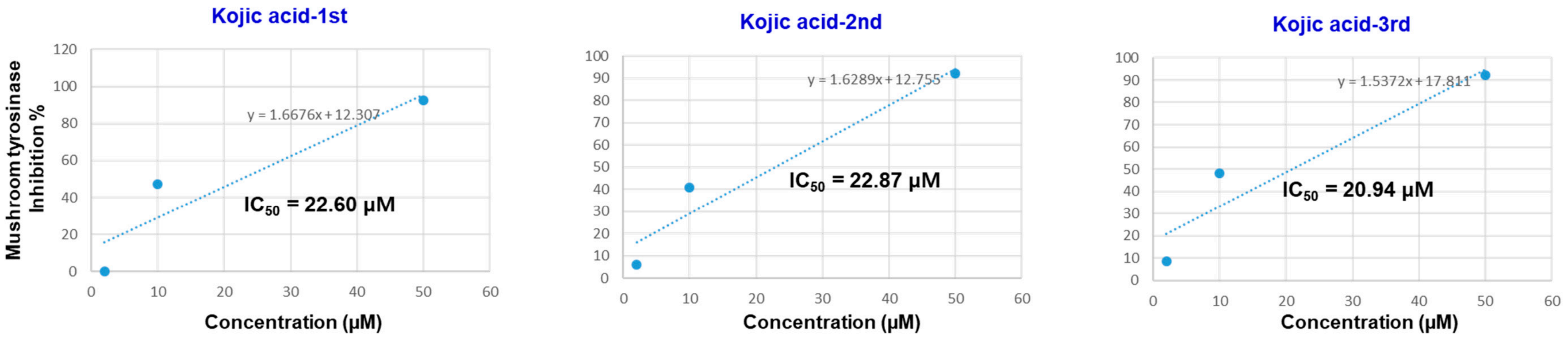

S28. Graphs used to calculate the IC_{50} value for kojic acid in the presence of L-tyrosine

Substrate: L-Dopa

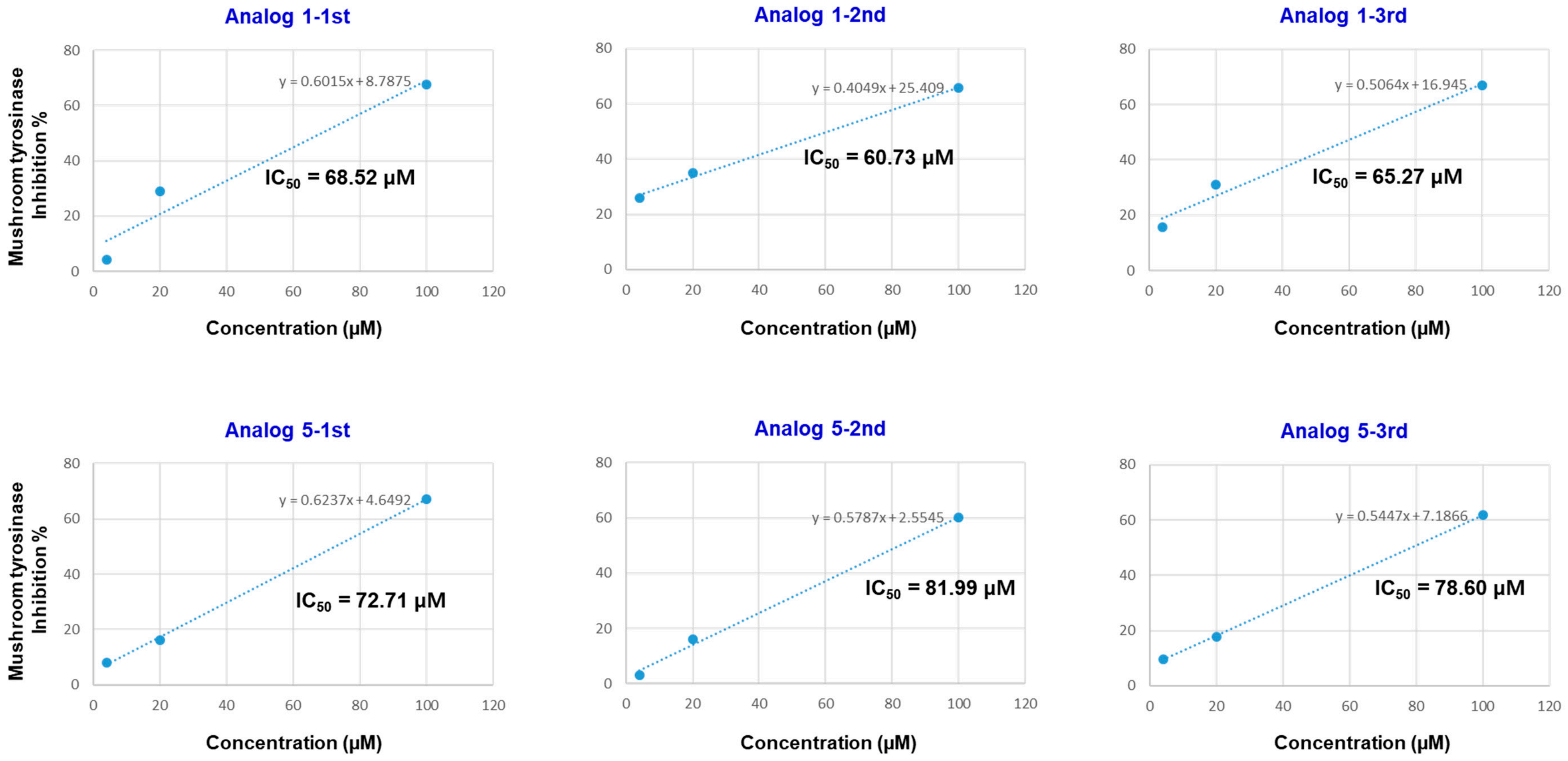

S29. Graphs used to calculate the IC_{50} values for analogs **1** and **5** in the presence of L-dopa

Substrate: L-Dopa

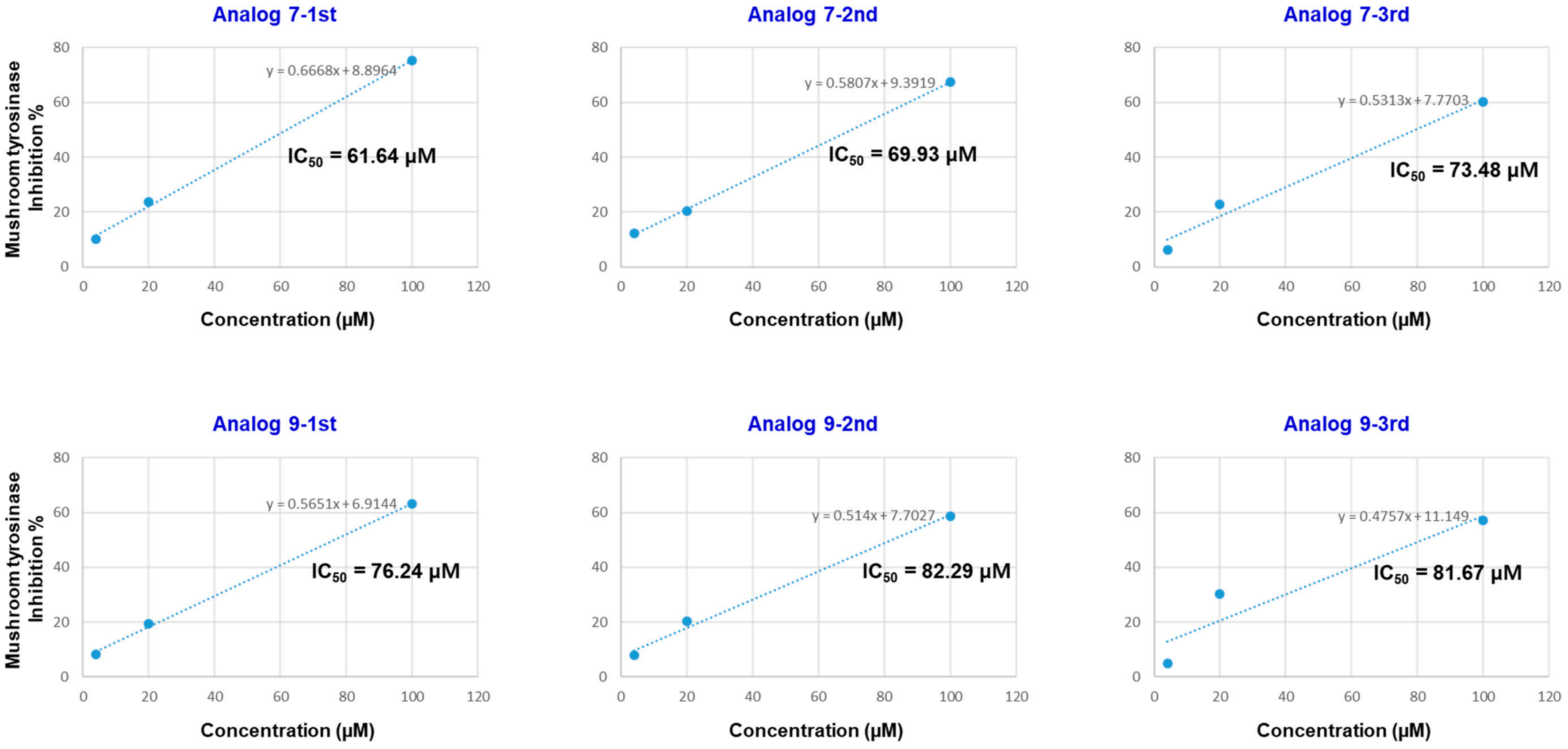

S30. Graphs used to calculate the IC_{50} values for analogs **7** and **9** in the presence of L-dopa

Substrate: L-Dopa

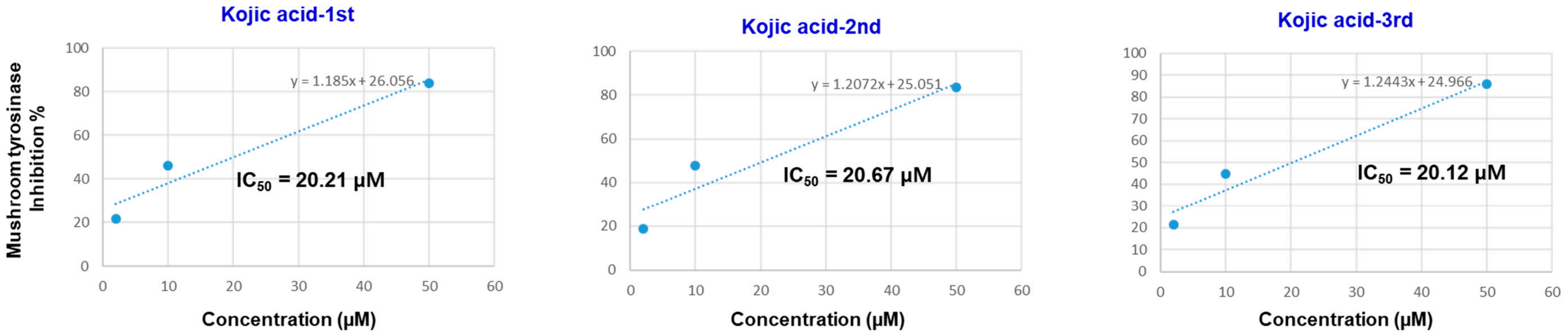

S31. Graphs used to calculate the IC_{50} value for kojic acid in the presence of L-dopa

(A)

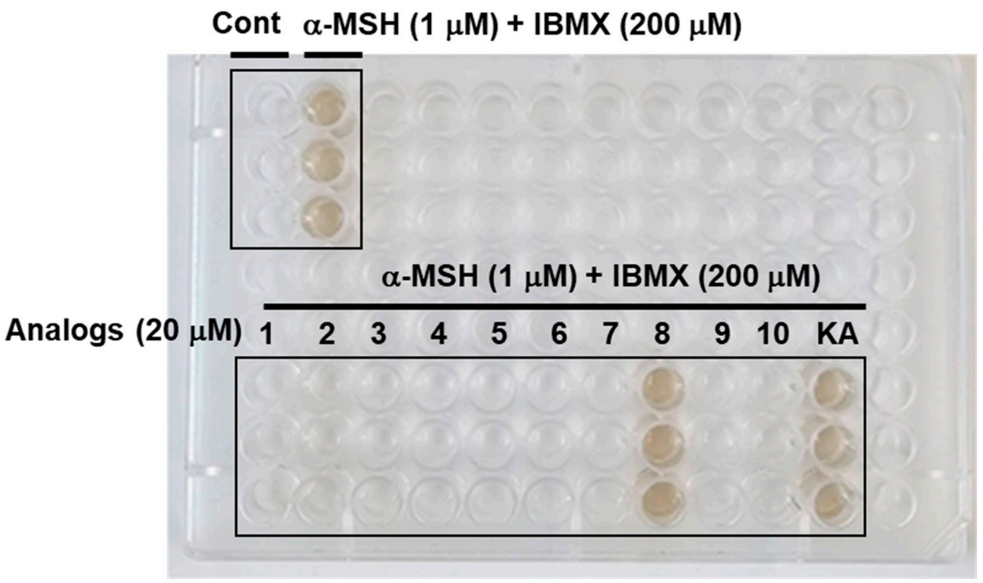

Cont: control
aM+IB: α-MSH + IBMX
M1128: analog **1**
M1129: analog **2**
M1130: analog **3**
M1131: analog **4**
M1132: analog **5**
M1133: analog **6**
M1134: analog **7**
M1135: analog **8**
M1136: analog **9**
M1137: analog **10**
KA: kojic acid

(B)

			Analogs										Kojic acid
	Control	αM+IB	M1128	M1129	M1130	M1131	M1132	M1133	M1134	M1135	M1136	M1137	KA
	95.641	317.949	131.966	135.214	101.282	102.821	132.051	112.564	175.299	333.504	94.9573	108.462	326.239
	99.7436	321.197	151.026	140.94	114.615	112.479	135.47	128.205	182.821	342.137	119.829	126.923	308.803
	104.615	345.983	159.744	151.88	114.188	116.923	142.991	130.256	199.658	370.769	118.034	145.641	266.838
aver	100	328.376	147.578	142.678	110.028	110.741	136.838	123.675	185.926	348.803	110.94	127.009	300.627

% average value based on the control (100%) (n=3)

S32. Photo (A) and original data (B) for melanin production results of **1**–**10** at 20 μM in B16F10 cells.

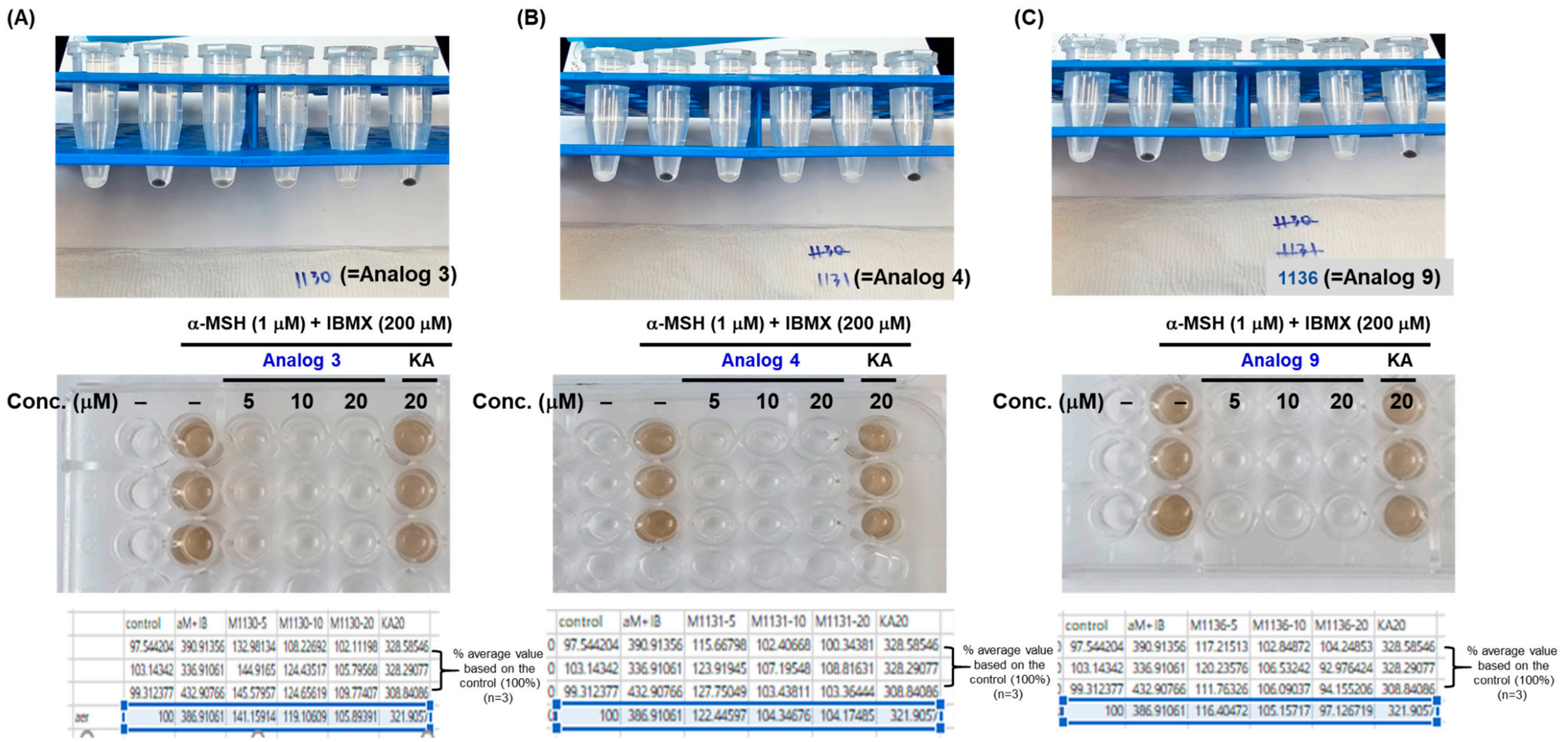

	control	aM+IB	M1130-5	M1130-10	M1130-20	KA20
	97.544204	390.91356	132.98134	108.22692	102.11198	328.58546
	103.14342	336.91061	144.9165	124.43517	105.79568	328.29077
	99.312377	432.90766	145.57957	124.65619	109.77407	308.84086
aer	100	386.91061	141.15914	119.10609	105.89391	321.9057

	control	aM+IB	M1131-5	M1131-10	M1131-20	KA20
0	97.544204	390.91356	115.66798	102.40668	100.34381	328.58546
0	103.14342	336.91061	123.91945	107.19548	108.81631	328.29077
0	99.312377	432.90766	127.75049	103.43811	103.36444	308.84086
0	100	386.91061	122.44597	104.34676	104.17485	321.9057

control	aM+IB	M1136-5	M1136-10	M1136-20	KA20
97.544204	390.91356	117.21513	102.84872	104.24853	328.58546
103.14342	336.91061	120.23576	106.53242	92.976424	328.29077
99.312377	432.90766	111.76326	106.09037	94.155206	308.84086
100	386.91061	116.40472	105.15717	97.126719	321.9057

aM+IB: α-MSH + IBMX KA20: kojic acid (20 μM)

M1130-5: analog **3** (5 μM)
M1130-10: analog **3** (10 μM)
M1130-20: analog **3** (20 μM)

M1131-5: analog **4** (5 μM)
M1131-10: analog **4** (10 μM)
M1131-20: analog **4** (20 μM)

M1136-5: analog **9** (5 μM)
M1136-10: analog **9** (10 μM)
M1136-20: analog **9** (20 μM)

S33. Melanin production results of analogs **3** (A), **4** (B), and **9** (C) at three different concentrations (5, 10, and 20 μM).

(A)

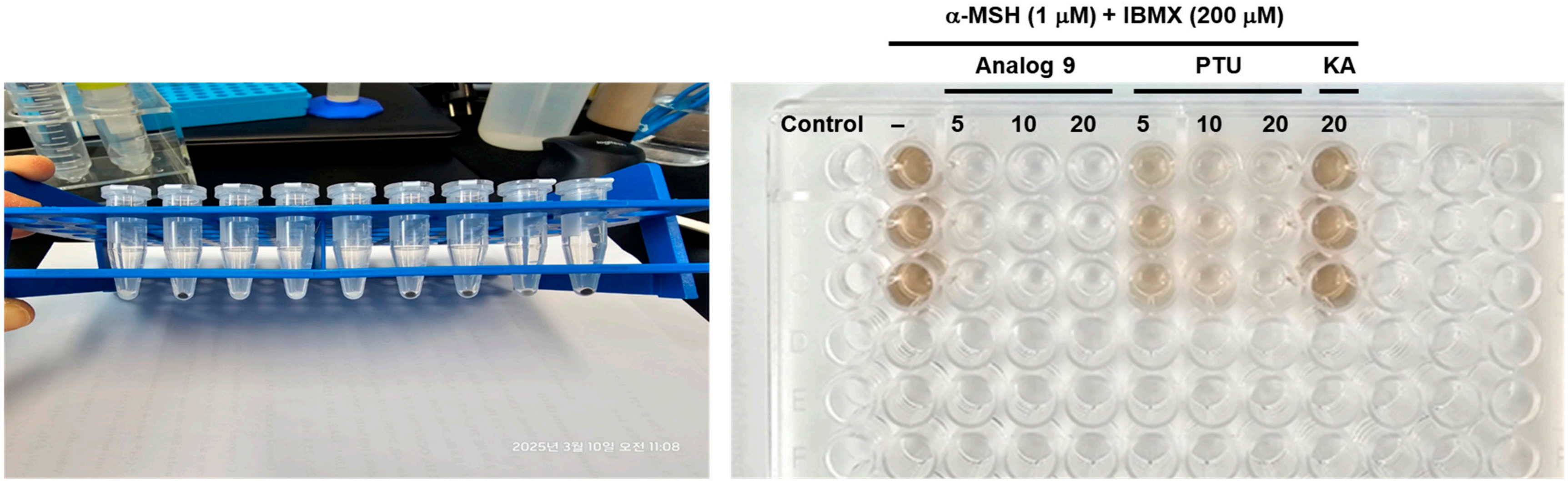

(B)

	Cont	aM+IB	M1136-5	M1136-10	M1136-20	PTU-5	PTU-10	PTU-20	KA20
	91.81254	434.3309	124.9294	111.7165	103.1621	246.8097	153.642	130.4348	343.7888
	103.6702	418.2383	115.528	105.3642	100.367	284.3309	189.3845	140.5985	341.2479
	104.5172	454.489	124.9294	102.3151	95.45455	287.5494	198.7013	136.8718	388.1705
aver	100	435.6861	121.7956	106.4653	99.66121	272.8967	180.5759	135.9684	357.7357

% average value based on the control (100%) (n=3)

Cont: control aM+IB: α-MSH + IBMX KA20: kojic acid (20 μM)
M1136-5: analog **9** (5 μM) PTU-5: PTU (5 μM)
M1136-10: analog **9** (10 μM) PTU-10: PTU (10 μM)
M1136-20: analog **9** (20 μM) PTU-20: PTU (20 μM)

S34. Photo (A) and original data (B) for melanin production results for analog **9** and PTU (positive control) in B16F10 cells.

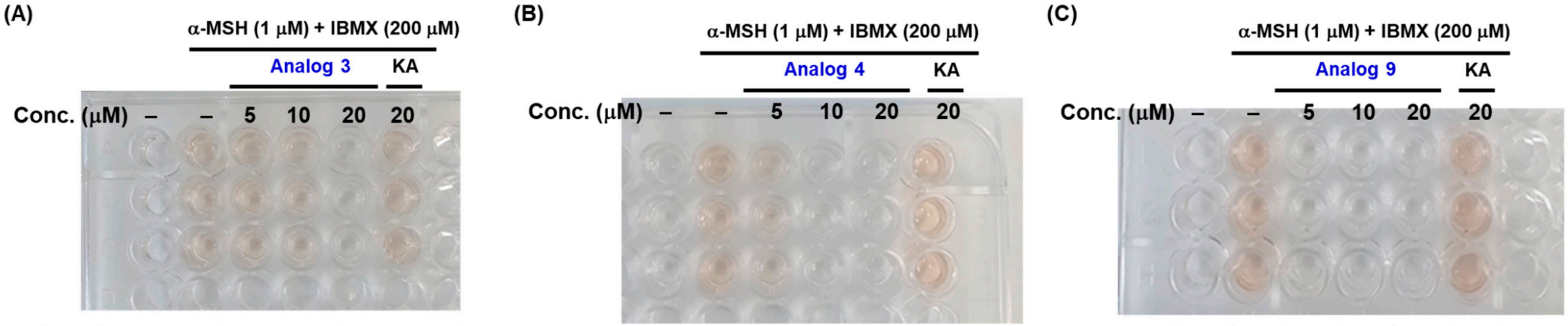

(A)

	control	aM+IB	M1130-5	M1130-10	M1130-20	KA20
n=1	98.8636	241.288	221.402	165.152	99.8106	215.341
n=2	97.9167	227.083	215.53	181.439	105.114	233.712
n=3	103.22	255.114	227.462	170.833	107.955	214.962

% average value based on the control (100%) (n=3)

(B)

	control	aM+IB	M1131-5	M1131-10	M1131-20	KA20
n=1	98.8636	241.288	183.712	92.4242	80.8712	215.341
n=2	97.9167	227.083	166.288	100	83.5227	233.712
n=3	103.22	255.114	170.265	98.2955	85.0379	214.962

% average value based on the control (100%) (n=3)

(C)

	control	aM+IB	M1136-5	M1136-10	M1136-20	KA20
n=1	98.8636	241.288	94.3182	84.8485	81.4394	215.341
n=2	97.9167	227.083	91.8561	85.2273	85.2273	233.712
n=3	103.22	255.114	119.318	87.5	83.7121	214.962

% average value based on the control (100%) (n=3)

aM+IB: α-MSH + IBMX KA20: kojic acid (20 μM)
M1130-5: analog **3** (5 μM)
M1130-10: analog **3** (10 μM)
M1130-20: analog **3** (20 μM)
M1131-5: analog **4** (5 μM)
M1131-10: analog **4** (10 μM)
M1131-20: analog **4** (20 μM)
M1136-5: analog **9** (5 μM)
M1136-10: analog **9** (10 μM)
M1136-20: analog **9** (20 μM)

S35. Effect of NBTC analogs **3** (A), **4** (B), and **9** (C) on cellular tyrosinase activity at three different concentrations (5, 10, and 20 μM) in B16F10 cells.

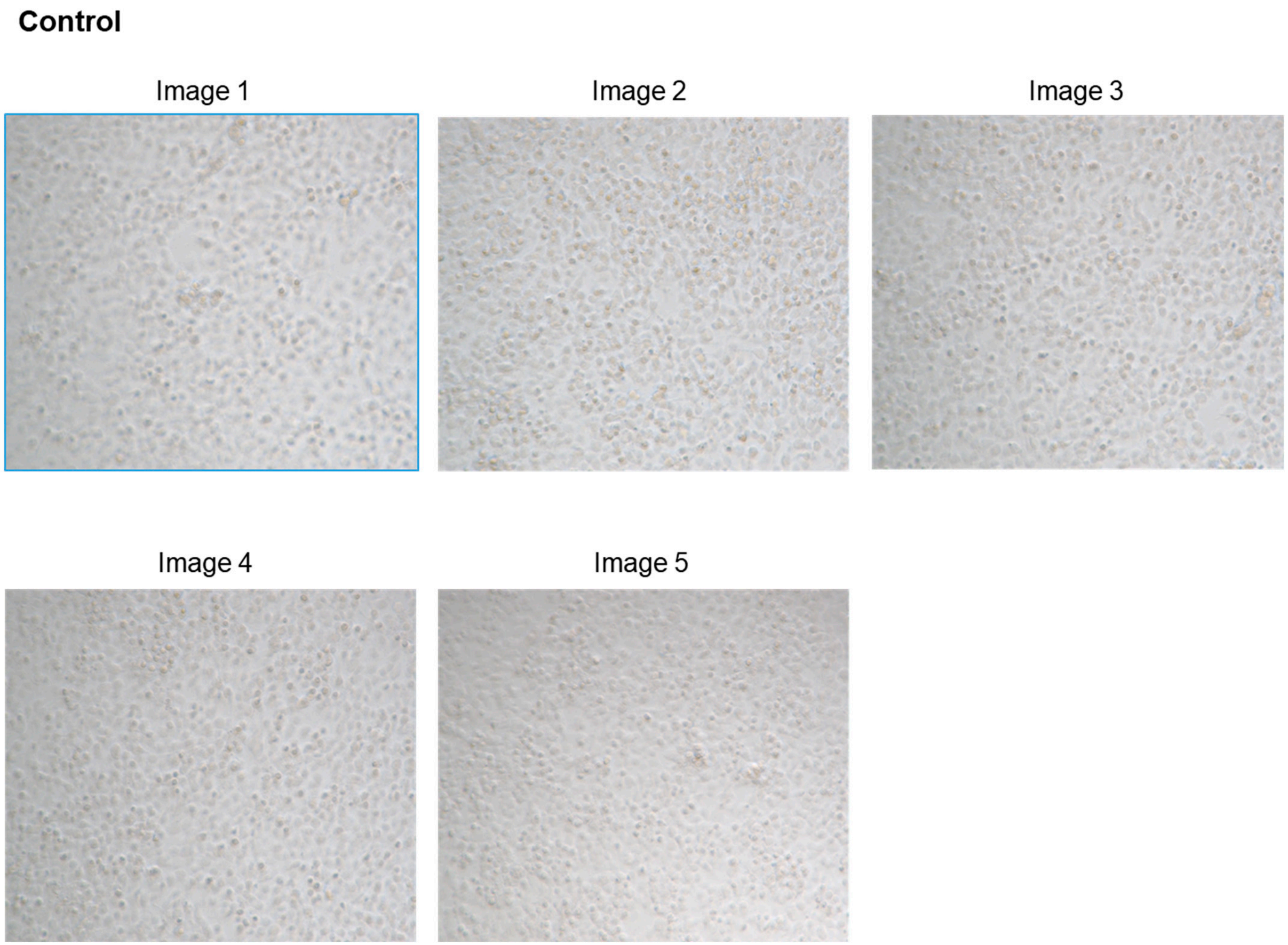

S36. Images of the control group (n = 5) in the in situ B16F10 cellular tyrosinase activity experiments.

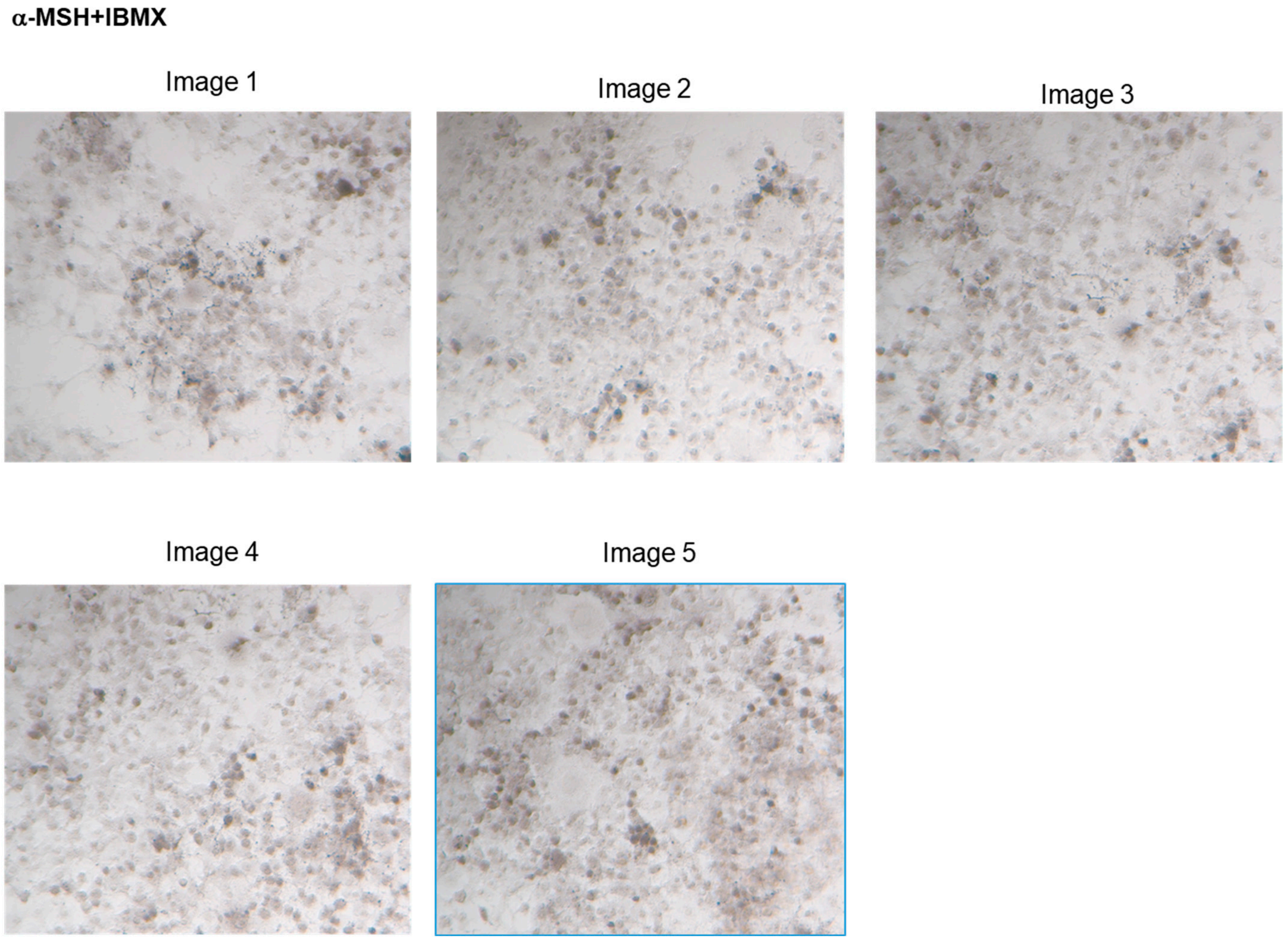

S37. Images of the α-MSH + IBMX group (n = 5) in the in situ B16F10 cellular tyrosinase activity experiments.

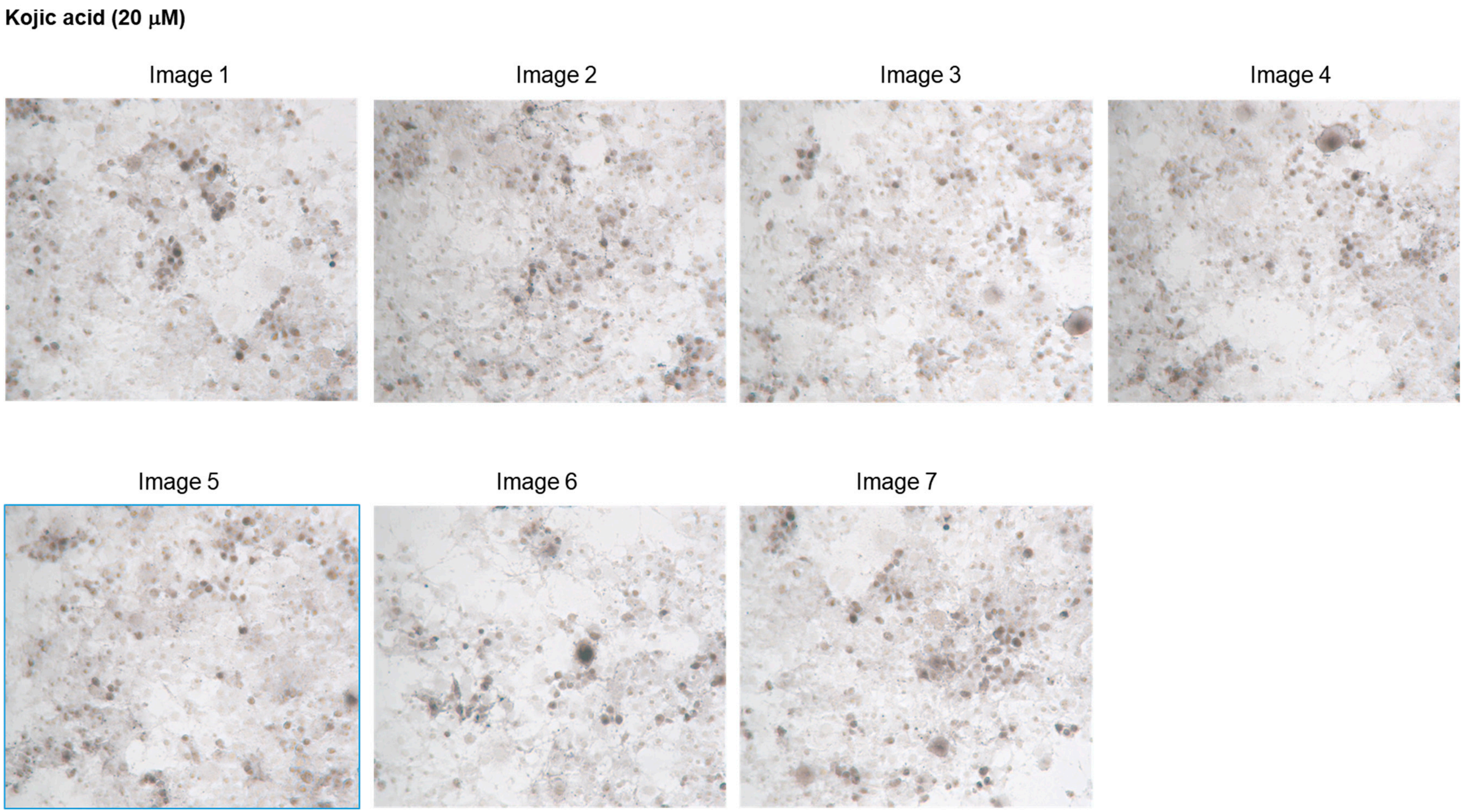

S38. Images of the kojic acid (20 μM) group (n = 7) in the in situ B16F10 cellular tyrosinase activity experiments.

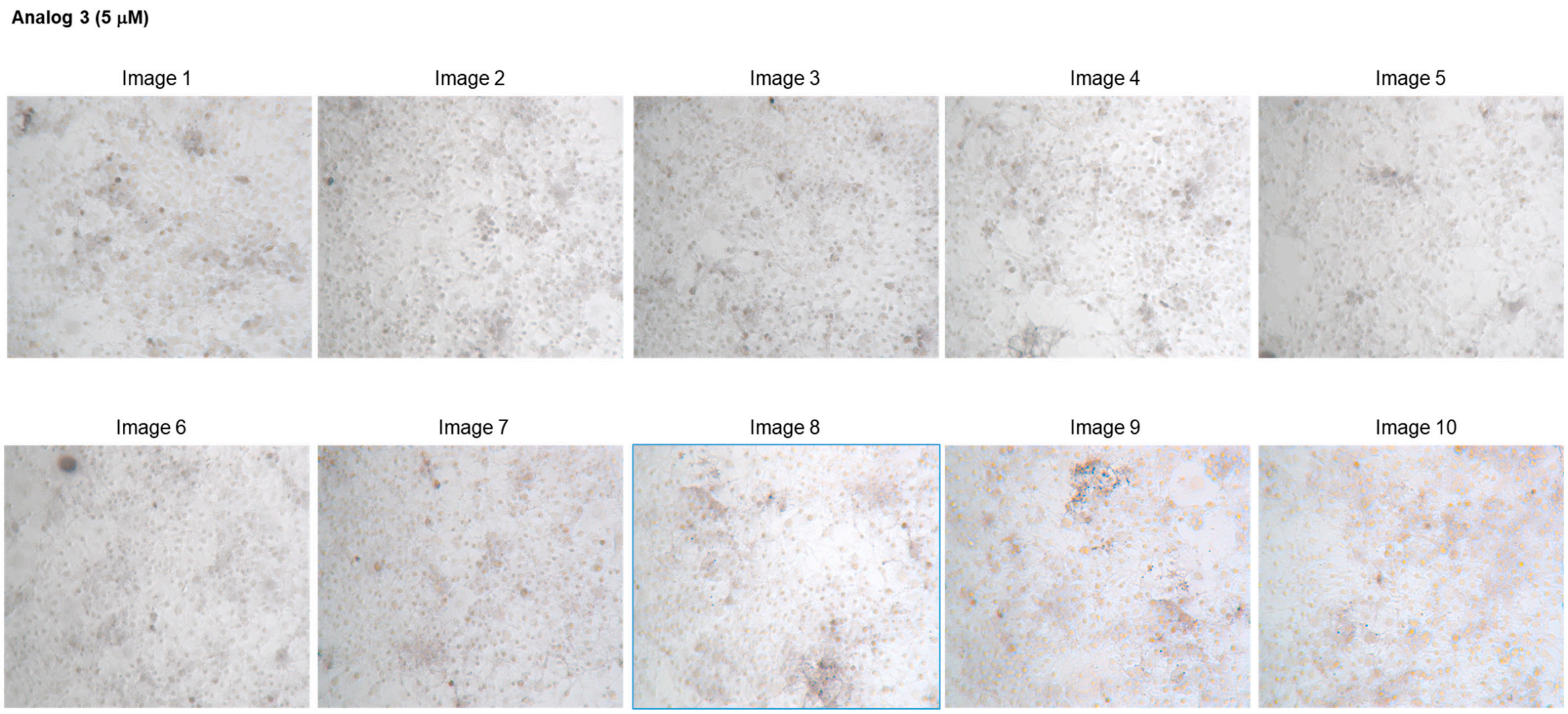

S39. Images of analog **3** (5 μM) group (*n* = 10) in the in situ B16F10 cellular tyrosinase activity experiments.

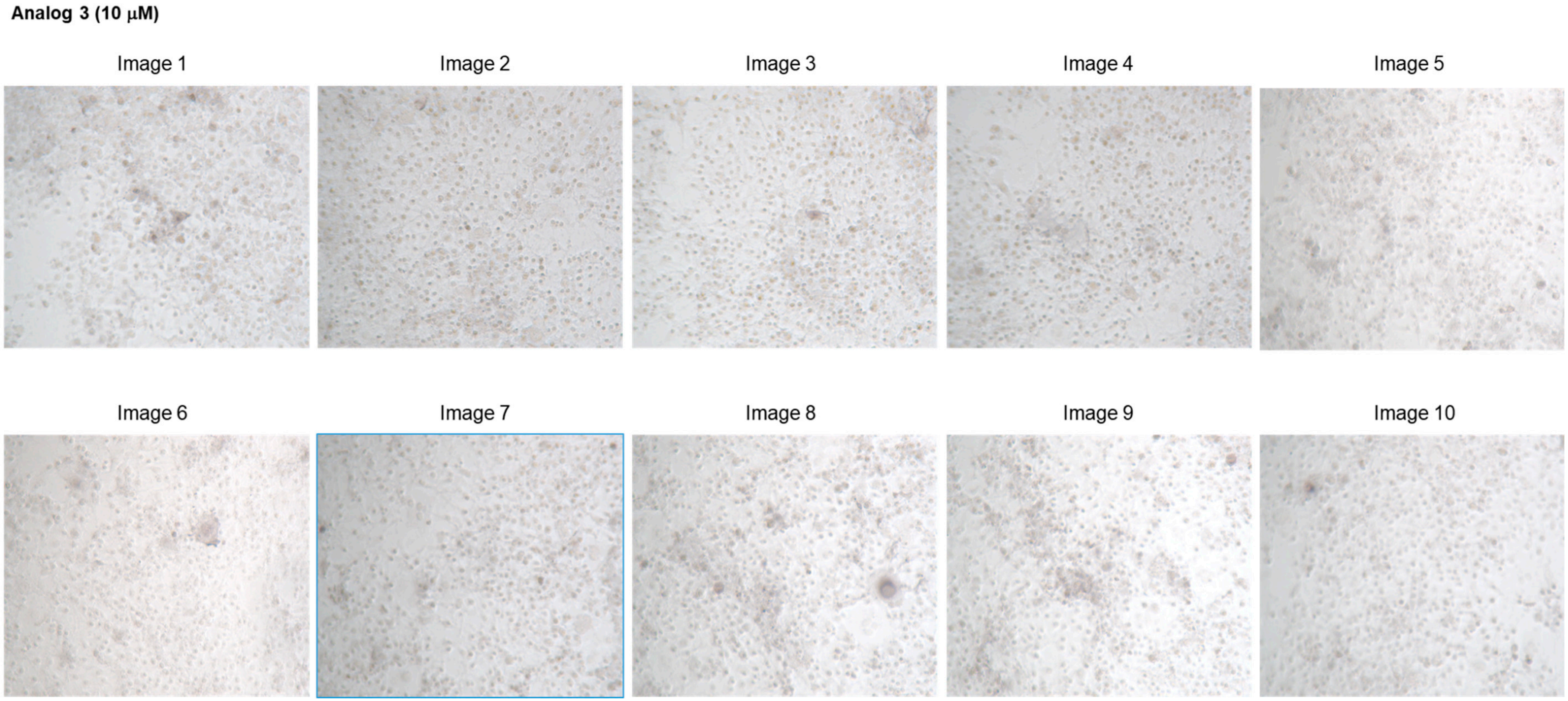

S40. Images of analog **3** (10 μM) group (*n* = 10) in the in situ B16F10 cellular tyrosinase activity experiments.

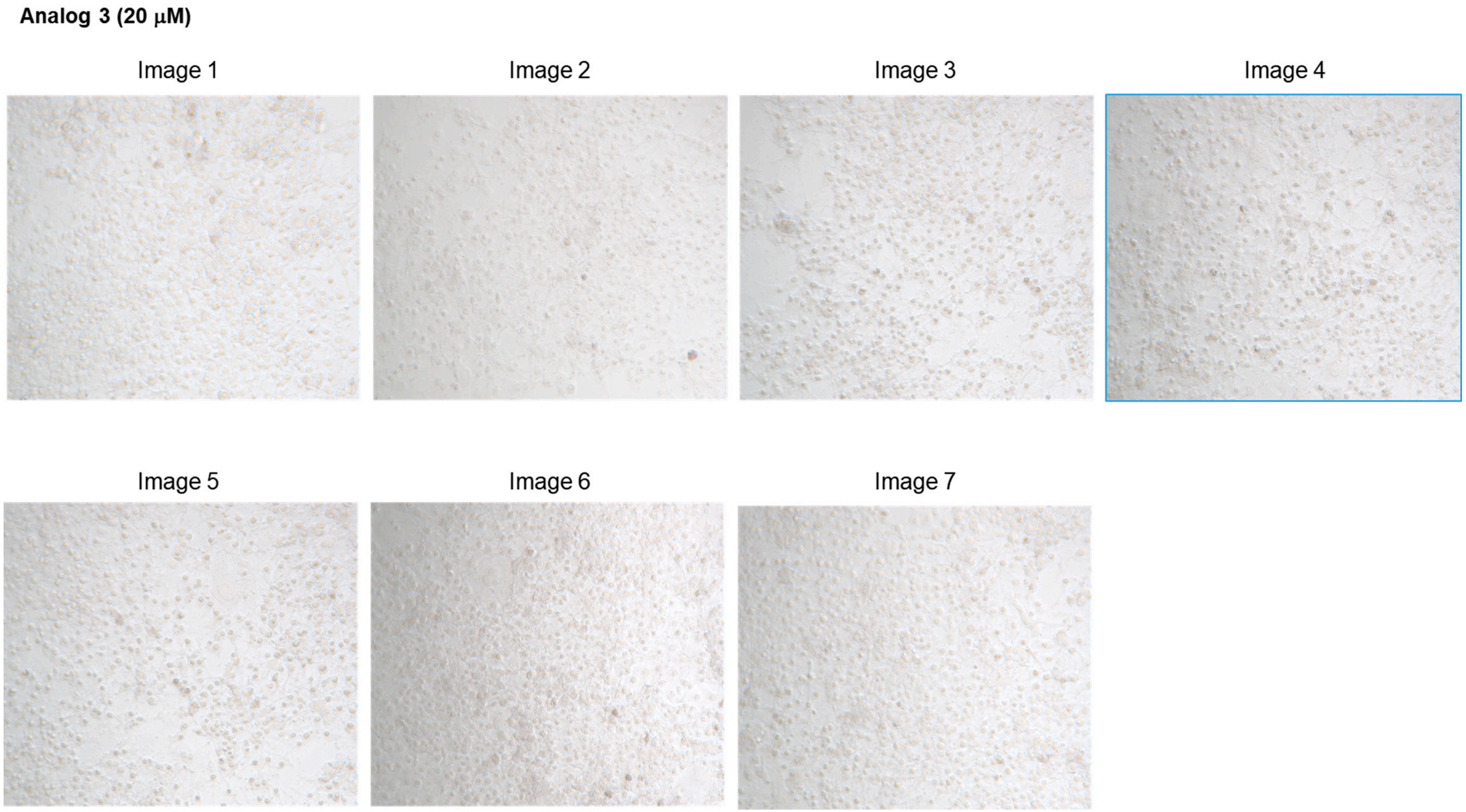

S41. Images of analog **3** (20 μM) group (*n* = 7) in the in situ B16F10 cellular tyrosinase activity experiments.

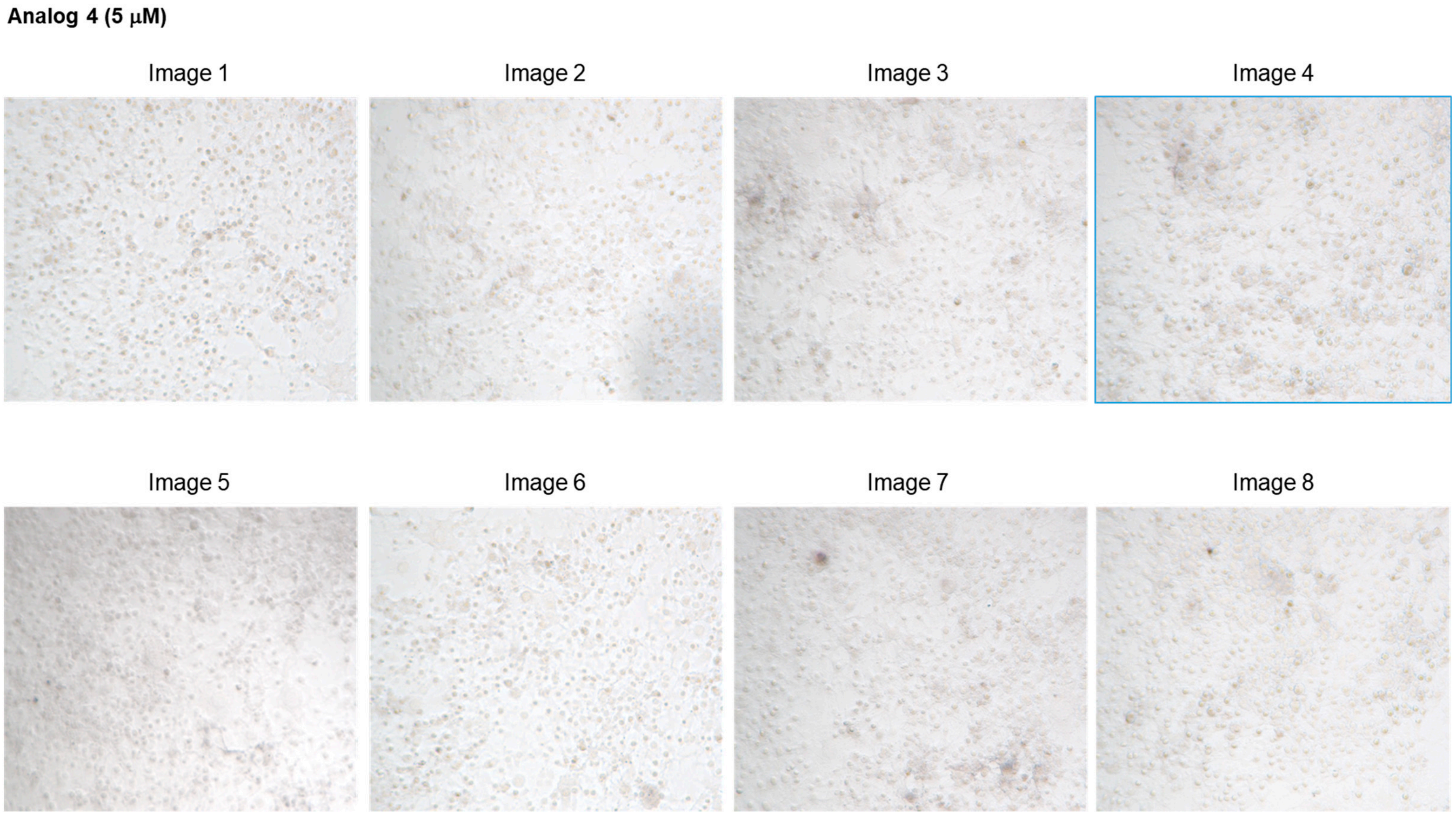

S42. Images of analog **4** (5 μM) group (*n* = 8) in the in situ B16F10 cellular tyrosinase activity experiments.

Analog 4 (10 μM)

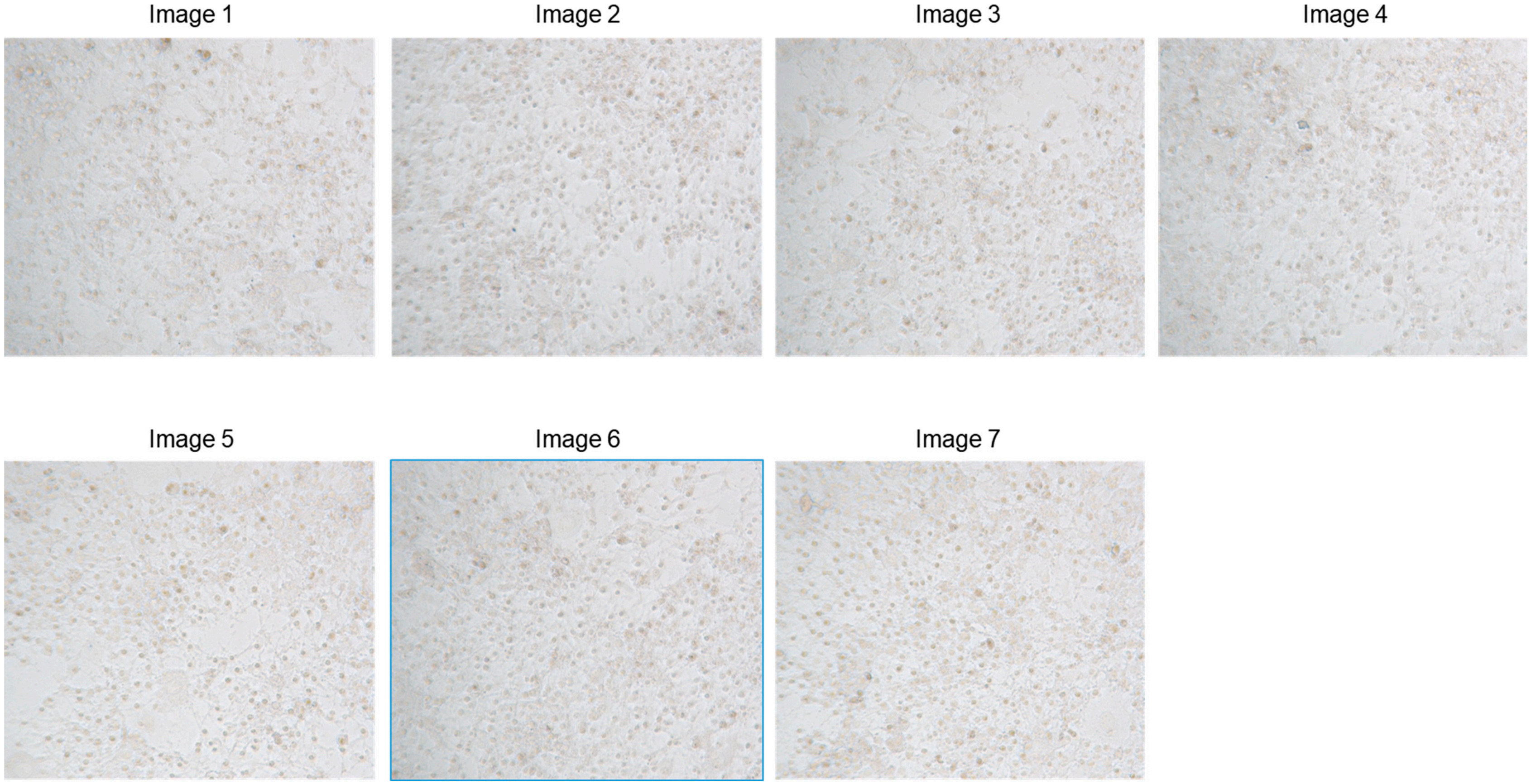

S43. Images of analog **4** (10 μM) group (*n* = 7) in the in situ B16F10 cellular tyrosinase activity experiments.

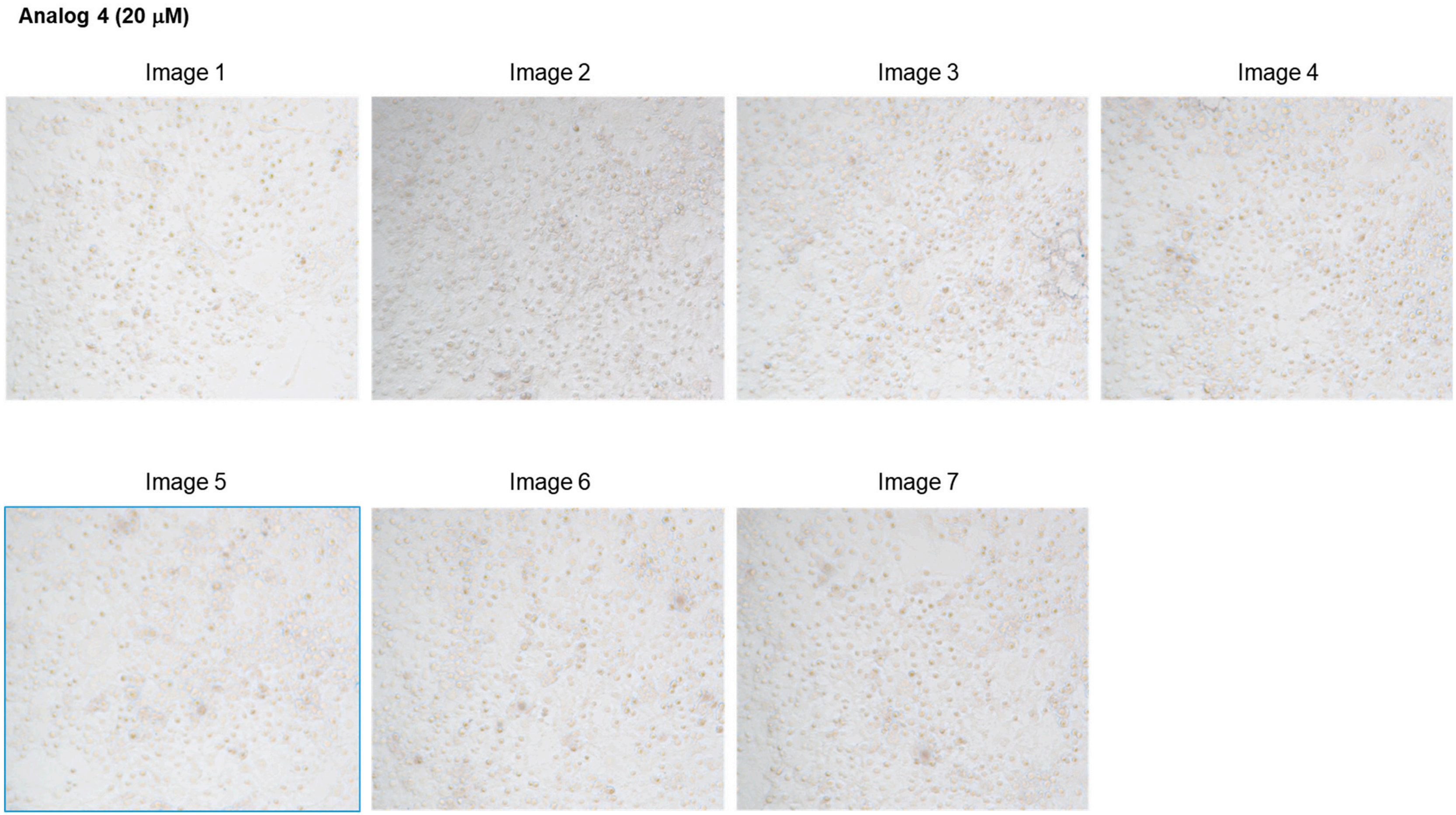

S44. Images of analog **4** (20 μM) group (*n* = 7) in the in situ B16F10 cellular tyrosinase activity experiments.

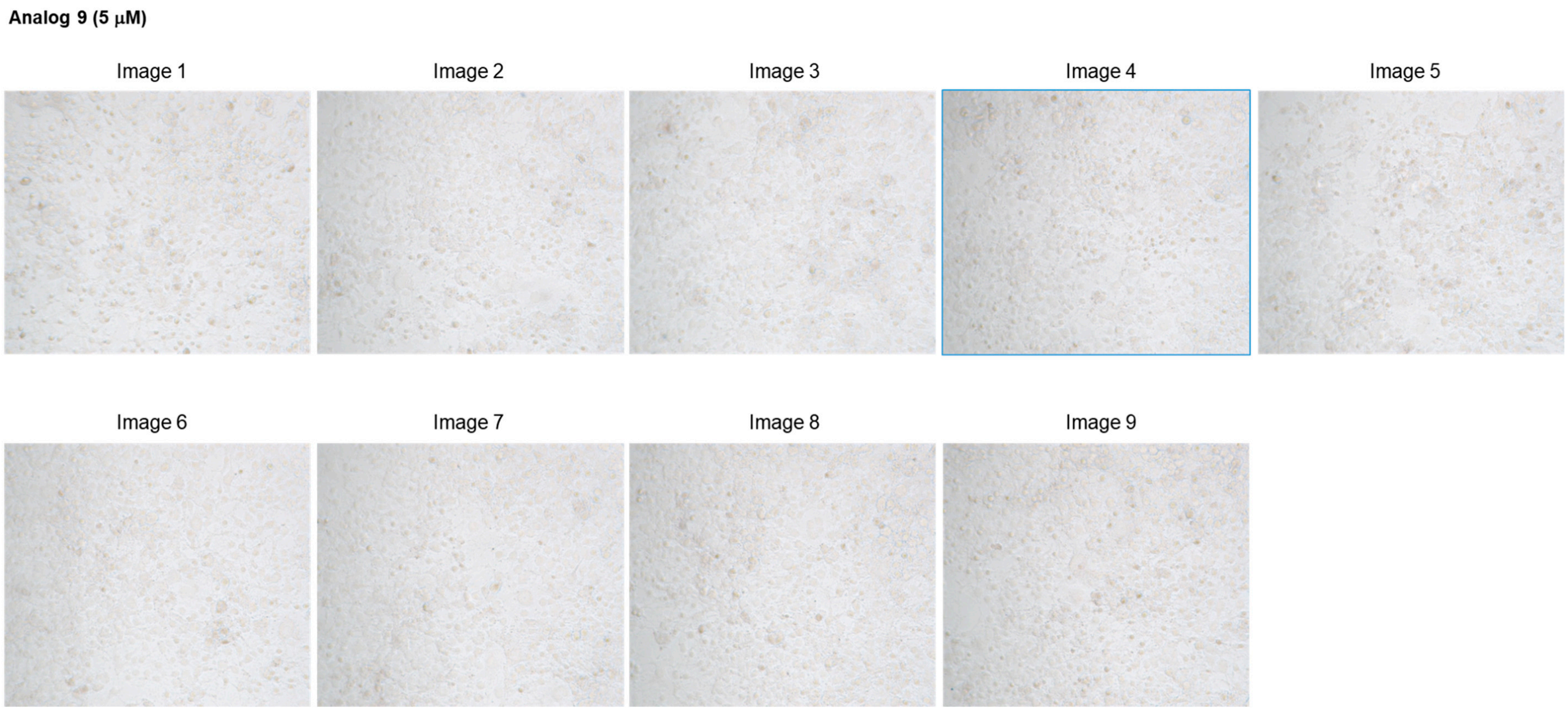

S45. Images of analog **9** (5 μM) group (*n* = 9) in the in situ B16F10 cellular tyrosinase activity experiments.

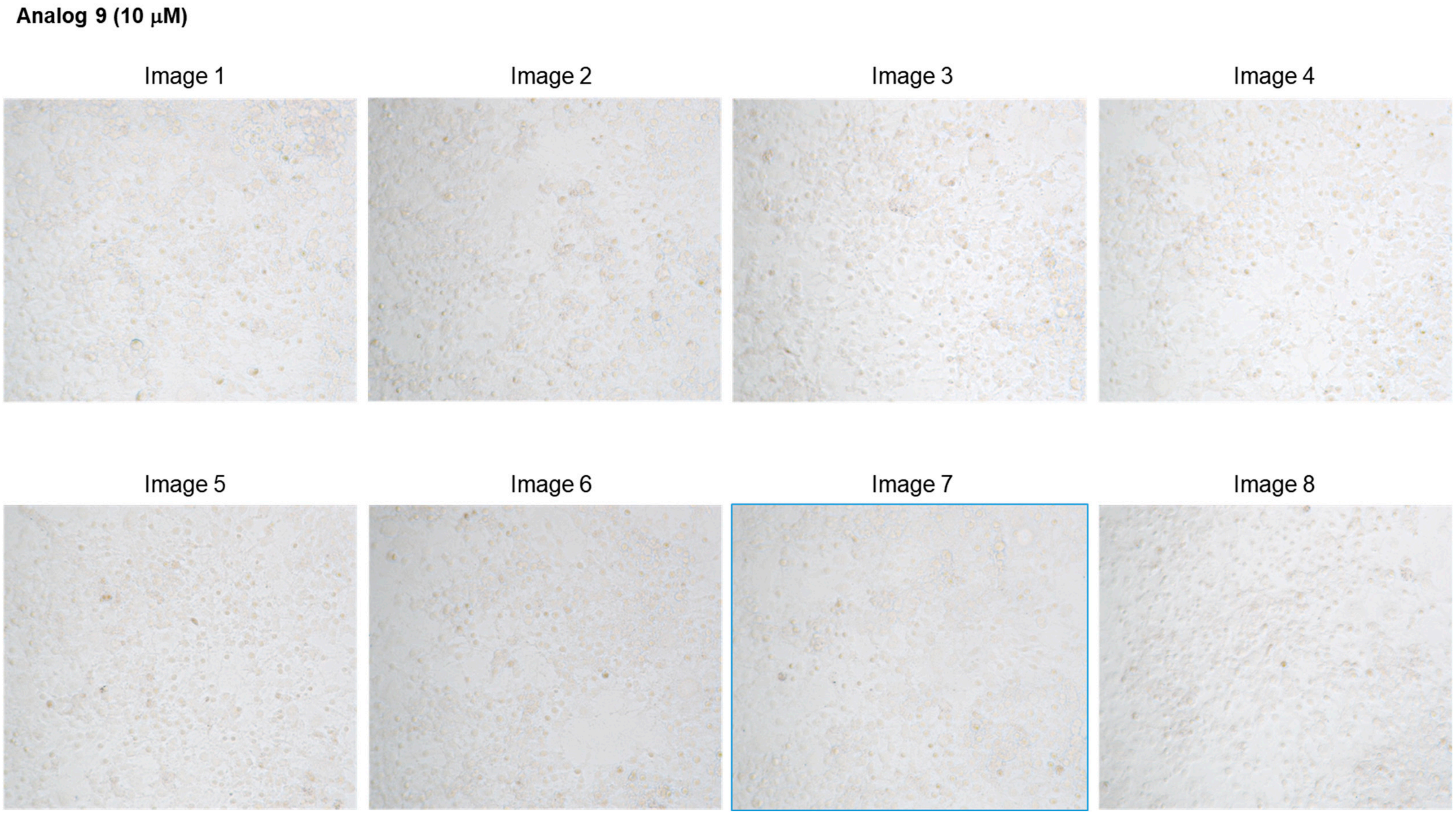

S46. Images of analog **9** (10 μM) group (*n* = 8) in the in situ B16F10 cellular tyrosinase activity experiments.

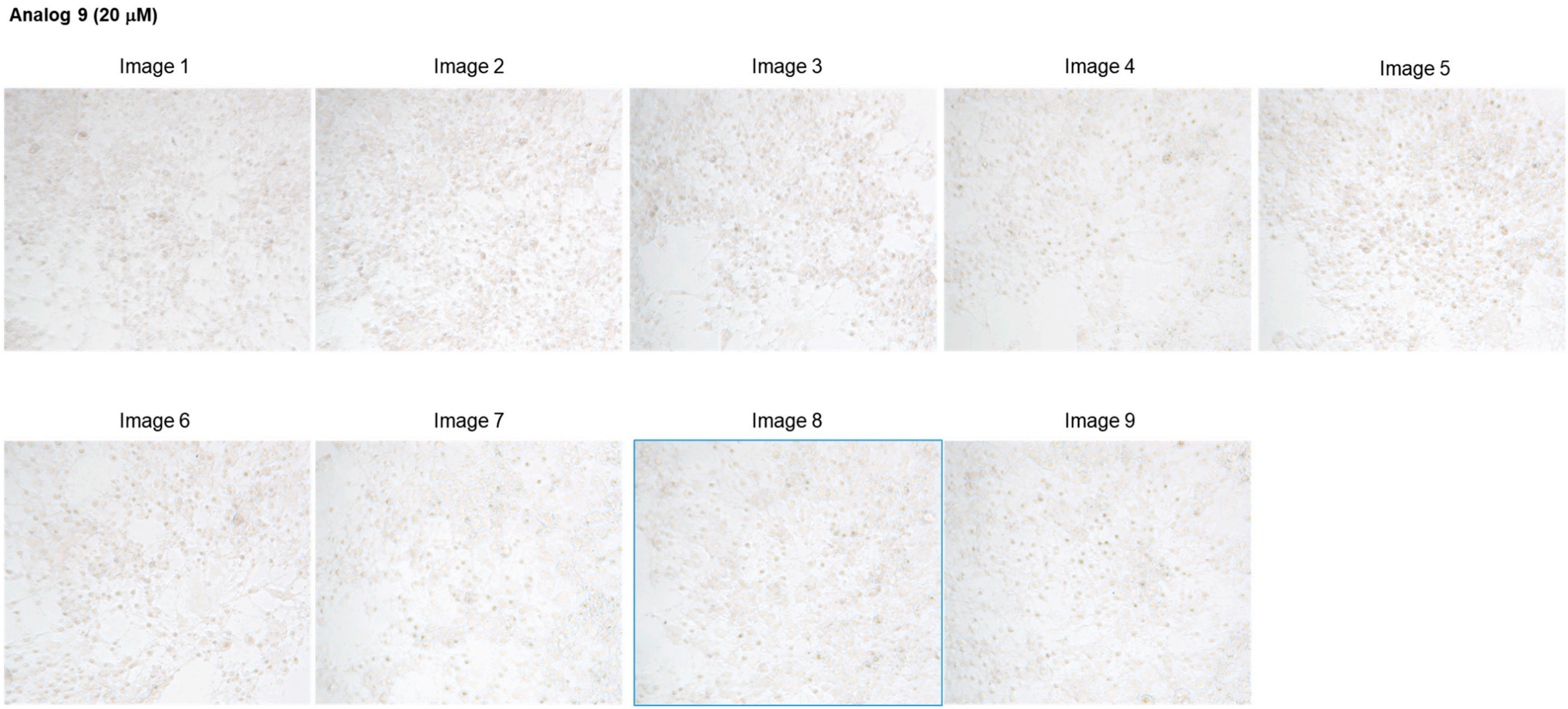

S47. Images of analog **9** (20 μM) group (*n* = 9) in the in situ B16F10 cellular tyrosinase activity experiments.

(A)

%	cont	aM+IB	KA	anal3-5	anal3-10	anal3-20	anal4-5	anal4-10	anal4-20	anal9-5	anal9-10	anal9-20
n=1	80.0248	238.854	209.268	144.649	113.843	121.303	123.845	140.815	93.6364	70.7728	79.1057	123.734
n=2	96.4001	217.154	242.619	184.452	134.839	129.436	170.182	98.3922	68.8084	112.311	88.5968	89.302
n=3	132.889	208.558	265.076	99.3375	112.01	109.841	93.6088	126.766	78.6593	85.9824	75.3142	93.5577
n=4	132.024	264.576	256.82	234.621	119.692	138.536	104.89	132.515	81.3995	123.563	70.0128	98.6366
n=5	58.6618	304.312	193.895	234.724	189.153	135.649	102.846	120.308	93.1643	112.347	95.393	48.7768
n=6			180.811	245.395	182.749	114.626	140.778	96.6588	97.5049	120.049	138.866	85.2709
n=7			242.648	219.061	216.347	162.211	101.839	70.2083	85.8269	132.978	114.489	66.4741
n=8				301.526	176.462		102.037			135.721	108.271	71.0066
n=9				228.48	277.237					117.111		65.1412
n=10				112.919	193.799							
aver	100	246.691	227.305	200.516	171.613	130.229	117.503	112.238	85.5714	112.315	96.256	82.4334

(B)

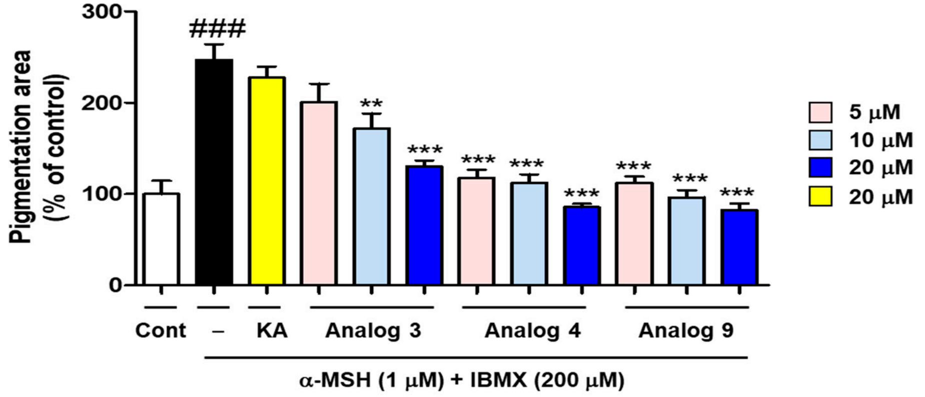

S48. Pigmentation area raw data (A) and analysis graph (B) for the L-dopa staining of analogs **3**, **4**, and **9** and kojic acid (KA).

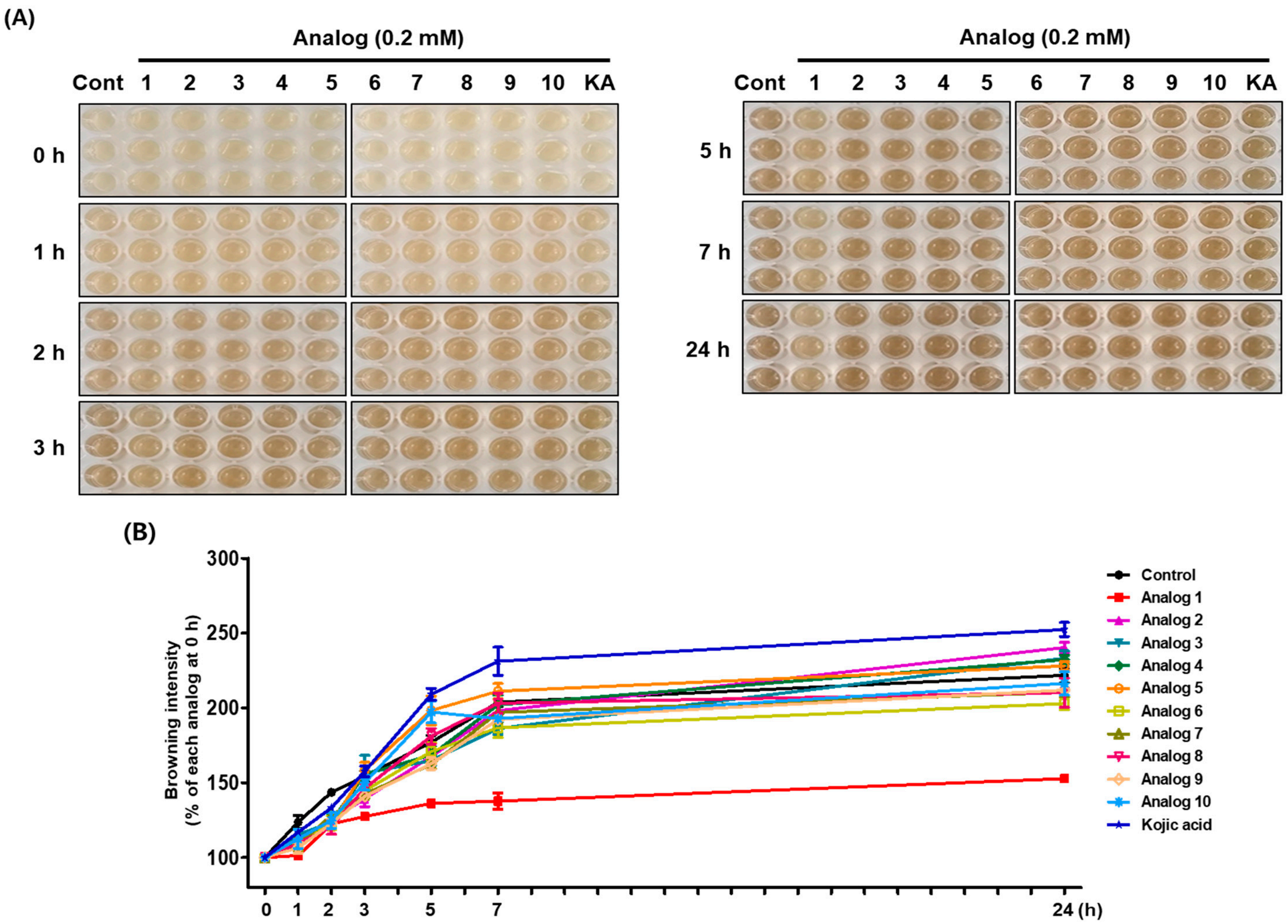

S49. Effect of NBTC analogs **1**–**10** on the browning of potato juice.

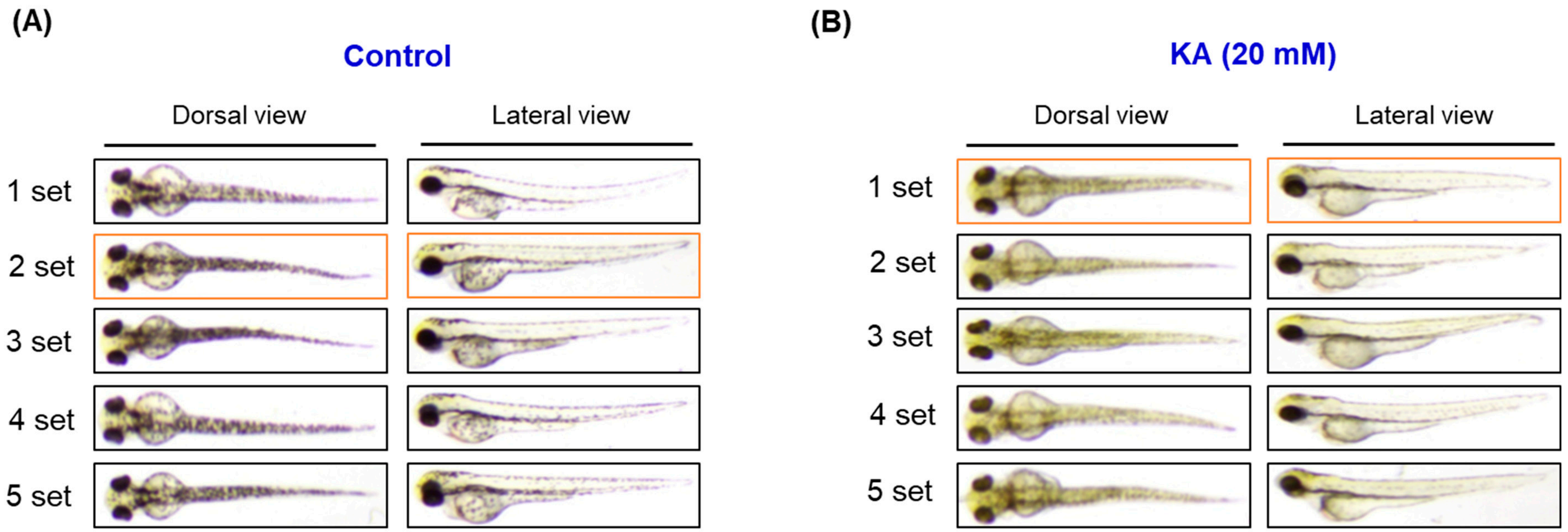

S50. Depigmentation results in dorsal and lateral views of zebrafish larvae treated with control (A) and KA (kojic acid, 20 mM) (B).

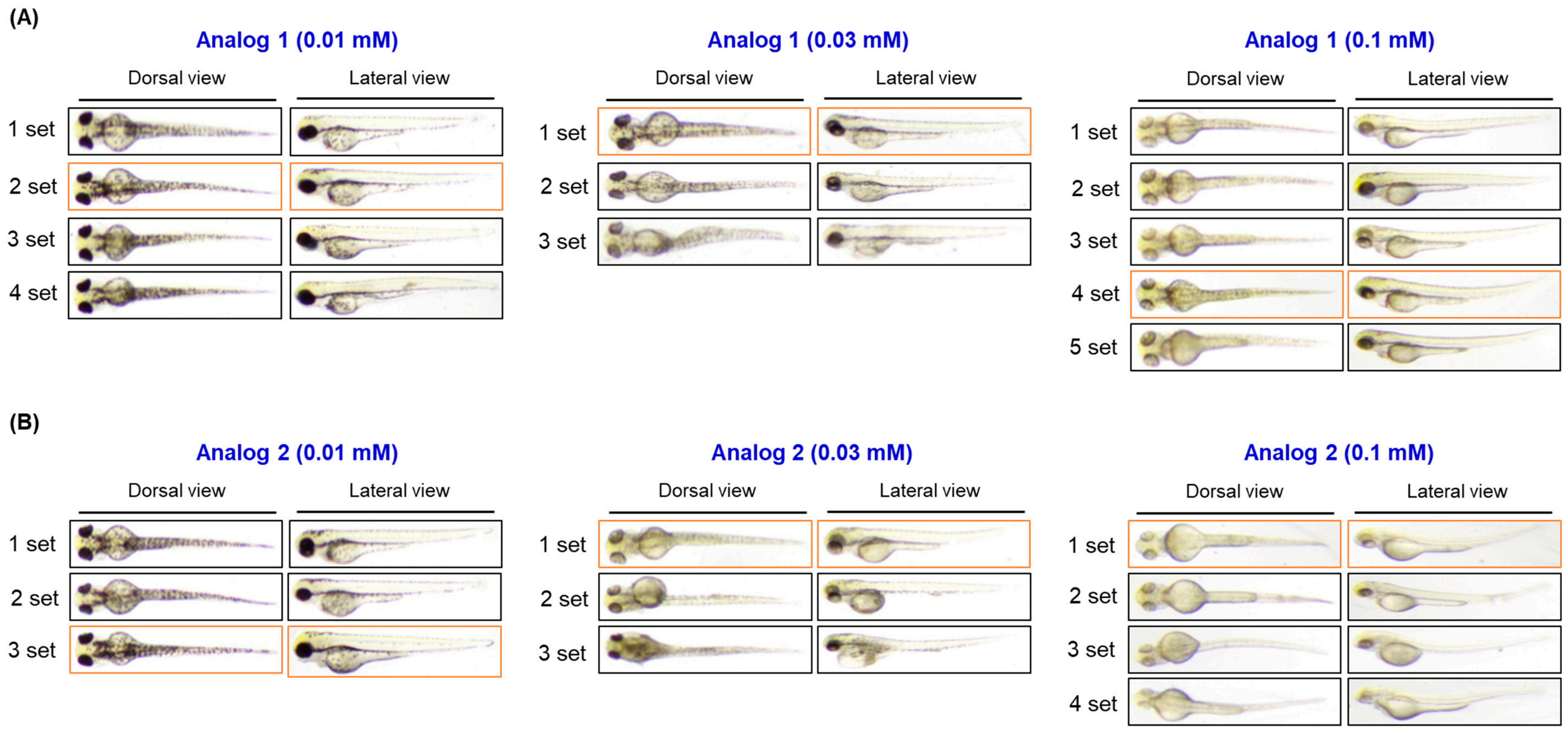

S51. Depigmentation results in dorsal and lateral views of zebrafish larvae treated with analog **1** (0.01, 0.03, and 0.1 mM) (A) and analog **2** (0.01, 0.03, and 0.1 mM) (B).

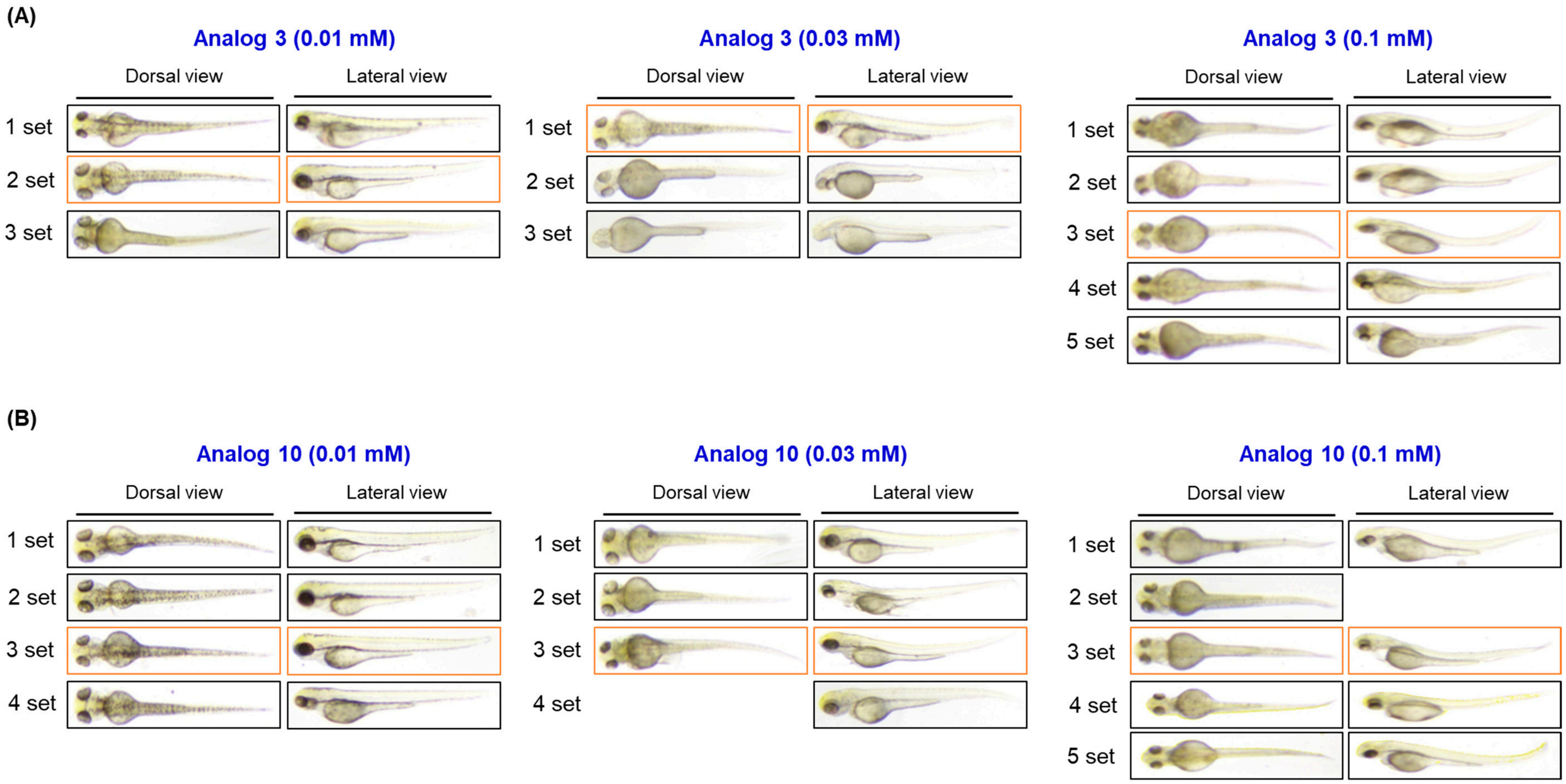

S52. Depigmentation results in dorsal and lateral views of zebrafish larvae treated with analog **3** (0.01, 0.03, and 0.1 mM) (A) and analog **10** (0.01, 0.03, and 0.1 mM) (B).

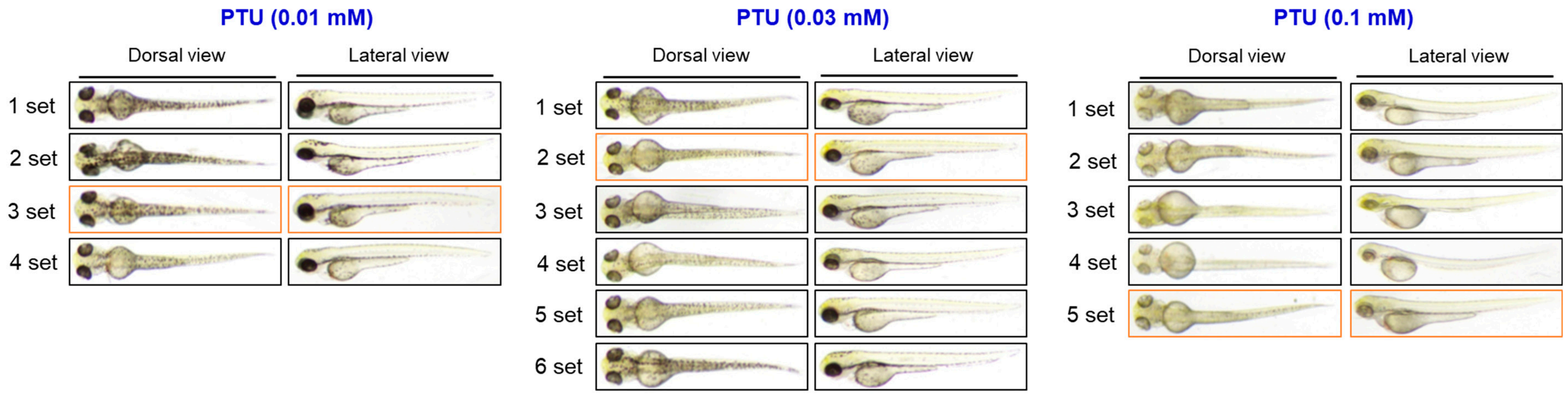

S53. Depigmentation results in dorsal and lateral views of zebrafish larvae treated with PTU (0.01, 0.03, and 0.1 mM).

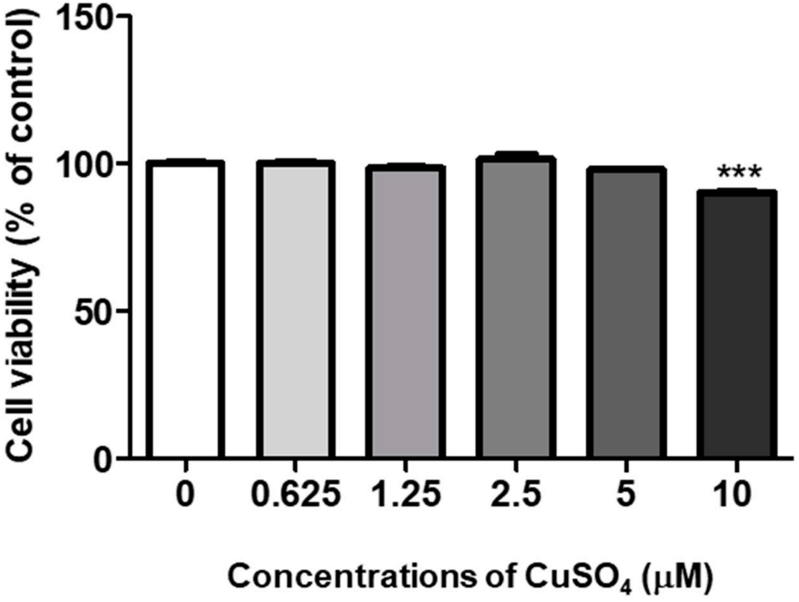

S54. Cell viability of $CuSO_4$ on B16F10 cells.

(A)

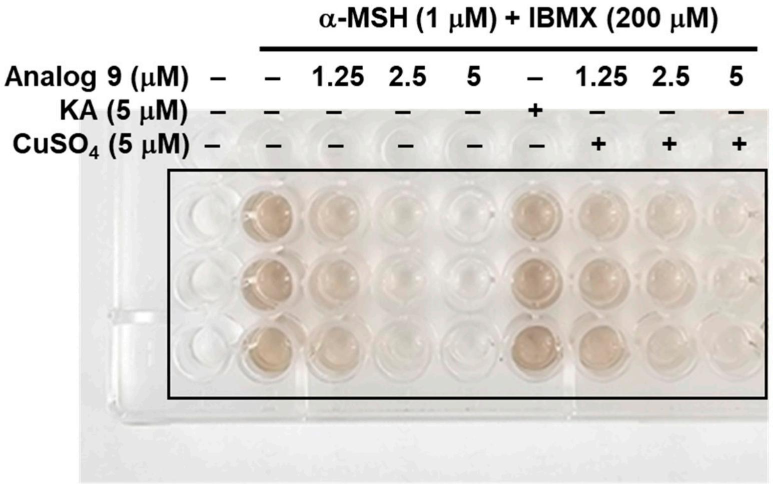

(B)

						add CuSO4 5uM		
control	aM+IB	M1136-1.25	M1136-2.5	M1136-5	KA-5	M1136-1.25-	M1136-2.5+	M1136-5+cuso4
106.9352	434.8274	237.219867	145.911569	79.9515445	324.530588	290.006057	241.58086	158.086008
95.94185	425.6511	251.211387	152.907329	95.0333131	383.858268	323.076923	259.751666	150.726832
97.12296	524.7729	275.287704	153.543307	120.109025	409.569958	365.687462	220.048455	181.34464

% average value based on the control (100%) (n=3)

aM+IB: α-MSH + IBMX
M1136-1.25: analog **9** (1.25 μM)
M1136-2.5: analog **9** (2.5 μM)
M1136-5: analog **9** (5 μM)
KA-5: kojic acid (5 μM)
M1136-1.25 + $CuSO_4$: analog **9** (1.25 μM) + $CuSO_4$ (5 μM)
M1136-2.5 + $CuSO_4$: analog **9** (2.5 μM) + $CuSO_4$ (5 μM)
M1136-5 + $CuSO_4$: analog **9** (5 μM) + $CuSO_4$ (5 μM)

S55. Photo (A) and original data (B) of melanin production results for analog **9** with or without $CuSO_4$ in B16F10 cells.

(A)

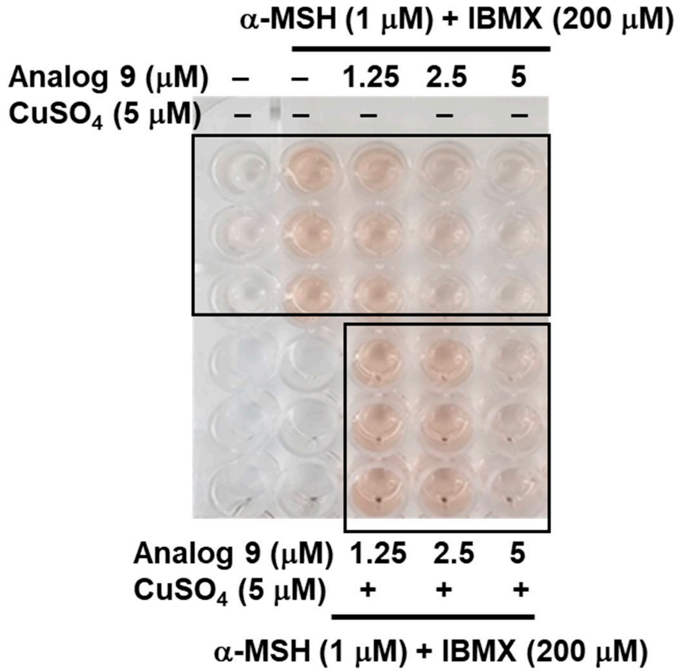

(B)

					add CuSO4 5uM		
control	aM+IB	M1136-1.:	M1136-2.!	M1136-5	M1136-1.	M1136-2.	M1136-5+cuso4
97.2085	239.573	219.048	180.131	110.345	281.117	258.949	169.458
108.046	257.471	225.123	196.388	123.153	277.176	272.906	170.443
94.7455	259.278	231.691	172.742	119.048	293.268	261.576	172.578

% average value based on the control (100%) (n=3)

aM+IB: α-MSH + IBMX
M1136-1.25: analog **9** (1.25 μM)
M1136-2.5: analog **9** (2.5 μM)
M1136-5: analog **9** (5 μM)
M1136-1.25 + $CuSO_4$: analog **9** (1.25 μM) + $CuSO_4$ (5 μM)
M1136-2.5 + $CuSO_4$: analog **9** (2.5 μM) + $CuSO_4$ (5 μM)
M1136-5 + $CuSO_4$: analog **9** (5 μM) + $CuSO_4$ (5 μM)

S56. Photo (A) and original data (B) of cellular TYR activity results for analog **9** with or without $CuSO_4$ in B16F10 cells.

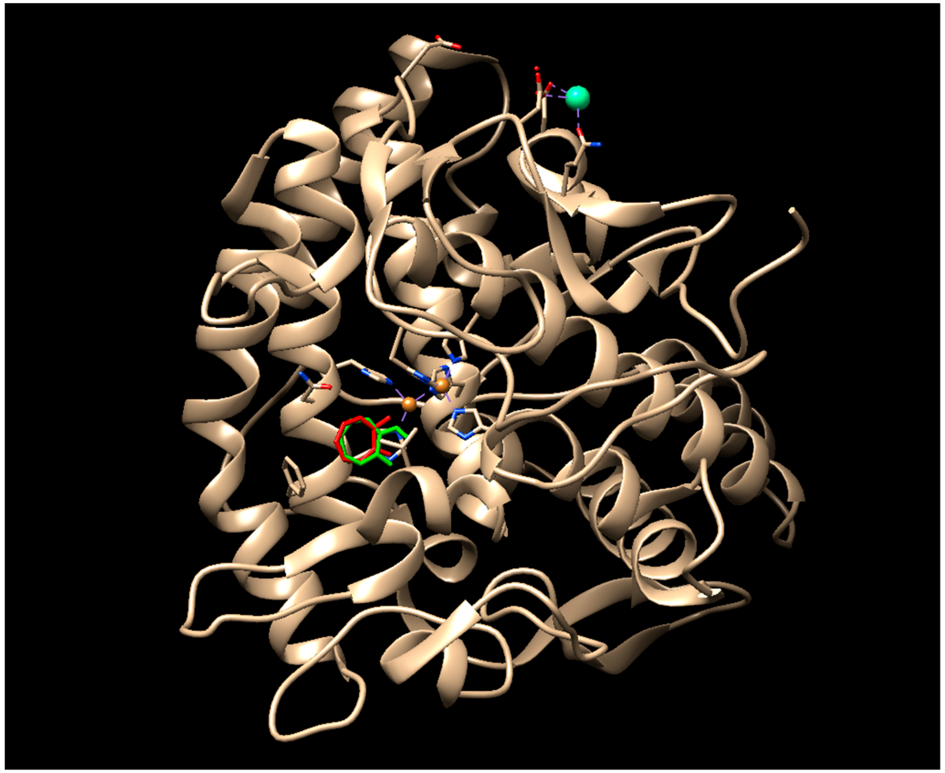

S57. Alignment of the re-docked ligand (green) and co-crystallized ligand (red) with the 2Y9X protein

The docking procedure was validated by redocking the co-crystallized tropolone at the tyrosinase active site. The redocking results revealed that the re-docked tropolone maintained the binding pose with a binding affinity of −5.5 kcal/mol. The co-crystallized and experimental poses had an RMSD of 0.61 Å. These findings indicate that the docking simulation could adequately accommodate the crystallized ligand.

Analog	**1**	**2**	**3**	**4**	**5**	**6**	**7**	**8**	**9**	**10**
Docking score (kcal/mol)	−5.7	−5.6	−5.3	−5.7	−5.4	−6.2	−6.6	−6.3	−6.4	−5.9

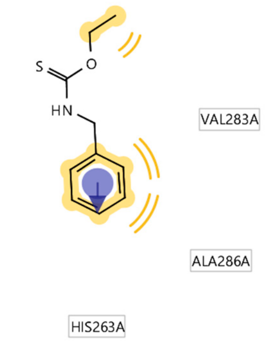

Derivative **2**

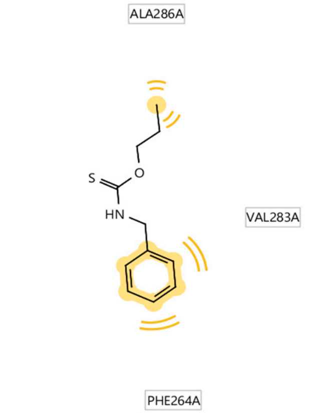

Derivative **3**

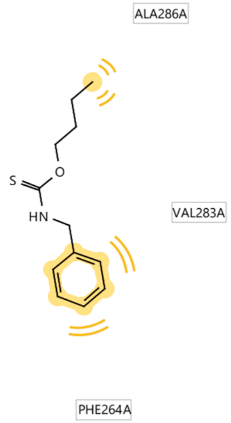

Derivative **4**

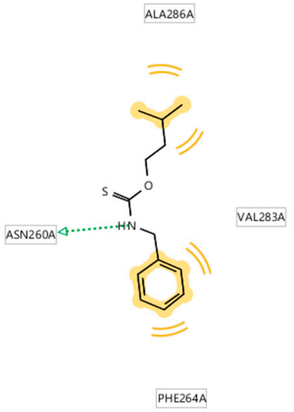

Derivative **6**

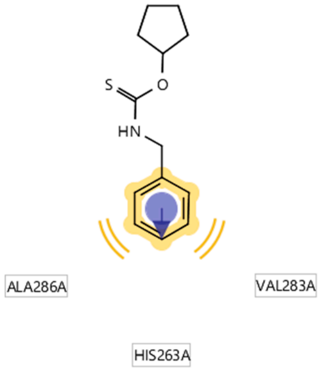

Derivative **8**

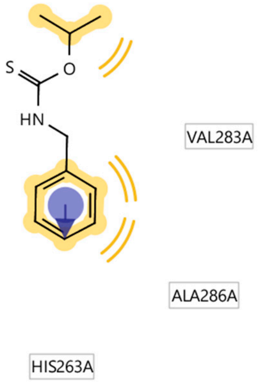

Derivative **10**

S58. Docking scores of analogs **1**–**10** and possible chemical interactions between analogs **2**–**4**, **6**, **8**, and **10** and mTYR amino acid residues.
